# Adaptation of Maize to Temperate Climates: Mid-Density Genome-Wide Association Genetics and Diversity Patterns Reveal Key Genomic Regions, with a Major Contribution of the *Vgt2* (*ZCN8*) Locus

**DOI:** 10.1371/journal.pone.0071377

**Published:** 2013-08-30

**Authors:** Sophie Bouchet, Bertrand Servin, Pascal Bertin, Delphine Madur, Valérie Combes, Fabrice Dumas, Dominique Brunel, Jacques Laborde, Alain Charcosset, Stéphane Nicolas

**Affiliations:** 1 UMR de Génétique Végétale, INRA – Université Paris-Sud – CNRS, Gif-sur-Yvette, France; 2 UMR444, Laboratoire de Genetique Cellulaire, INRA, Castanet-Tolosan, France; 3 UR1279, Etude du Polymorphisme des Génomes Végétaux, INRA, Commissariat à l'Energie Atomique (CEA) Institut de Génomique, Centre National de Génotypage, Evry, France; 4 INRA Stn Expt Mais, St Martin De Hinx, France; Kansas State University, United States of America

## Abstract

The migration of maize from tropical to temperate climates was accompanied by a dramatic evolution in flowering time. To gain insight into the genetic architecture of this adaptive trait, we conducted a 50K SNP-based genome-wide association and diversity investigation on a panel of tropical and temperate American and European representatives. Eighteen genomic regions were associated with flowering time. The number of early alleles cumulated along these regions was highly correlated with flowering time. Polymorphism in the vicinity of the *ZCN8* gene, which is the closest maize homologue to *Arabidopsis* major flowering time (*FT*) gene, had the strongest effect. This polymorphism is in the vicinity of the causal factor of *Vgt2* QTL. Diversity was lower, whereas differentiation and LD were higher for associated loci compared to the rest of the genome, which is consistent with selection acting on flowering time during maize migration. Selection tests also revealed supplementary loci that were highly differentiated among groups and not associated with flowering time in our panel, whereas they were in other linkage-based studies. This suggests that allele fixation led to a lack of statistical power when structure and relatedness were taken into account in a linear mixed model. Complementary designs and analysis methods are necessary to unravel the architecture of complex traits. Based on linkage disequilibrium (LD) estimates corrected for population structure, we concluded that the number of SNPs genotyped should be at least doubled to capture all QTLs contributing to the genetic architecture of polygenic traits in this panel. These results show that maize flowering time is controlled by numerous QTLs of small additive effect and that strong polygenic selection occurred under cool climatic conditions. They should contribute to more efficient genomic predictions of flowering time and facilitate the dissemination of diverse maize genetic resources under a wide range of environments.

## Introduction

Maize was domesticated in tropical conditions in the lowlands of southwest Mexico and later adapted to the broadest range of climatic conditions of all crops, from 40°S in Chile to 50°N in Canada and Russia, from sea level in the West Indies to elevations above 3400 m in the Andes [1]–[4]. Maize has high molecular diversity, with landraces pooling most of the nucleotide diversity (83%) from their wild ancestors, contrary to many other species [1]. This illustrates that a limited bottleneck occurred during domestication [2]–[4], probably due to the landrace outcrossing mating system and the continuous gene flow between cultivated and wild Zea mays L. subspecies. Modern breeding seems to have had little impact on genome-wide diversity and mostly affected genes that had already undergone selection during domestication [1], [2].

Despite this limited loss of diversity, phenotypes have been dramatically modified by domestication, large-scale migration/adaptation and selection cycles that gave rise to modern hybrids. In particular, flowering time evolved to adapt to short growing seasons, long days and low temperatures under temperate climates. Among the germplasm available, for maize landraces, the time from planting to the mature grain stage ranges from 2 to 11 months [3]. Silk emergence date (female flowering) varies by 32 days among the founder lines of thenested association mapping (NAM) population and by 28 days among the recombinant inbred lines derived from these parents [4].

Flowering time plays a key role in the acclimation of plants to different environments by integrating diverse environmental and endogenous signals that control the optimal moment for the transition from the vegetative to the reproductive phase. It allows the plant to avoid drought and thus kernel abortion, or to optimize the light interception period and yield for instance. The synchrony of male and female flowering is also an important adaptive trait in maize as high asynchrony can result in yield losses, especially in modern uniform varieties [5].

Compared to some other species like Arabidopsis [6] sorghum [7] and rice [8], [9], for which natural variations at a limited number of genes have been shown to have a large effect, flowering time architecture in maize is more complex. Several tens of small effect QTLs have been detected [4], [10]. This suggests that maize flowering involves a network of genes interacting in many signaling pathways. Among the loci that have been highlighted, the maize INDETERMINATE1 (*ID1*) gene is an important regulator of maize autonomous flowering that acts in leaves to mediate the expression of mobile signals that are hypothetical flowering hormones called florigens [11], [12], [13] which promote flowering at the shoot apical meristem. ZCN8 was found to be controlled by *ID1* and to express a florigen in leaves [14]. It is homologous to the Arabidopsis *FLOWERING LOCUS T* (*FT*), a kinase regulator [15]. FT is a key integrator because almost all flowering pathways (autonomous, gibberellins, photoperiod and vernalization) converge on it, and FT transmits the floral inductive signal to downstream floral identity genes [16]. In maize, a family of 25 FT homologues including *ZCN8* have been published [17]. They are named *Zea CENTRORADIALIS* (*ZCN*) genes. Expression analysis demonstrated that some of them are involved in developmental processes. A second gene that has been shown to have a major downstream effect is *Dfl1* (*Delayed flowering1*), a transcription factor that expresses in the shoot apical region [18]. Mutants, however, exhibit a less severe flowering time defect compared to *ID1*. Another major factor in flowering time variation, *Vgt1*, was detected in both linkage-based QTL analyses and association genetics studies [4], [10], [19], [20], [21], [22], [23], [24]. It has been cloned and described as a regulatory factor that controls the expression of an Apetala2-like gene, *ZmRap2.7* [24].

Further investigations to identify the main factors controlling maize flowering time in a panel representative of diverse migration routes would be beneficial: (i) to gain insight into the adaptation mechanisms under changing environments, (ii) to identify alleles for introgression into existing varieties in order to adapt them to different environmental conditions, and (iii) to better predict flowering time to the benefit of global crop management and local breeding programs, since flowering time is often considered as a major covariate in yield estimation. This latter objective can presently be approached through genomic selection models. The choice of suitable models should consider the complexity of trait architectures in terms of relative individual effects of loci and possible existence of non-additive effects due to interaction with the genetic background (GxQ epistasis) or gene-environment interactions (GxE) [25], [26], [27]. In maize, neither Buckler *et al.* [4] nor Steinhoff et al. [28] identified major effects for GxQ or GxE interactions for photoperiod insensitive QTLs of flowering time. They concluded that a simple additive model can accurately predict flowering time, in contrast to the genetic architecture observed in the selfing plant species like rice and *Arabidopsis*. Steinhoff *et al.* [28], however observed some regions presenting epistatic interactions between chromosomes 4 and 8, and between chromosome 9 and chromosomes 2, 7 and 8, so these questions generally remain open.

High density genotyping tools available today are expected to help in the discovery, fine mapping and allele diversity characterization of regions involved in flowering time. However, the choice of panel is very important as the level of polymorphism in each genetic group will determine the power of the analysis. In domesticated species like maize, loci that are critical to both local adaptation and yield performance, such as flowering time loci, are often targets of both natural and artificial selection, leading to complex forms of allele sharing and admixture among diverse genetic groups. Differentiation of flowering time between maize genetic groups is actually clear at the QTL level [29], [30]. Genome-wide association mapping and selection scans can provide complementary information to help decipher the architecture of such adaptive traits. For example, in the case of extreme differentiation leading to fixation of different alleles in different groups, the loci will be undetectable when association genetics approaches are used that include structure in the model, but will show significant tests of selection.

This study was thus designed to assess the potential of currently available mid-density arrays [31] in order to gain further insight into the maize flowering time architecture. We analyzed a panel representing a broad range of lines adapted to different environments (tropical lines, Corn-Belt Dents and Stiff Stalk, Northern and European Flints) that has proven efficient in previous flowering time candidate gene-based association studies [21], [30], [32]. We therefore first compared the structure of the panel as obtained with different marker sets in order to use the most appropriate one for statistical analyses. In order to determine the extent and variation of LD [33] and to estimate the number of markers required to cover the genome for future exhaustive genome scans, we looked at LD using an unbiased measure that extracts the part of the correlation of allelic frequencies that is due to the intrinsic structure of the panel [34]. We then looked at diversity, LD patterns and selection signature along the genome. We conducted association studies focused on female (FFLW) and male flowering (MFLW) dates and anthesis-to-silking date intervals (ASI). The selection test and association study results were considered to identify key genomic regions involved in adaptation. We compared diversity and recombination patterns in flowering time QTLs compared to the rest of the genome in order to identify putative selective events that may have shaped flowering time along ancient migration routes.

## Materials and Methods

### Genetic materials

A sample of 375 maize lines representing the worldwide diversity was considered [30]. A conformity check of newly extracted DNA samples compared to reference DNA samples revealed 10 illegitimate or strongly divergent samples that were removed (see Data S1 in Text S1). Among the 365 remaining lines genotyped with the 50K Illumina array, 29 were removed since they had more than 10% missing data or 5% heterozygosity. Among the remaining 336 lines that presented good quality SSR and Illumina genotyping, the panel was composed of five genetic groups according to the STRUCTURE results obtained with 55 SSRs [30]. The respective contribution to each group was calculated as the sum of quantitative assignments of all lines to this group, which led to 57 Northern Flints (NF), 62 European Flints (EF), 26 Iowa Synthetic Stiff Stalks (SS), 115 Corn Belt Dents (CBD) and 76 tropical lines (Trop). Overall, 242 lines were assigned to one group (with a major contribution of >80%) and are further referred to as “non-admixed” lines. This sub-sample of relatively non-admixed lines consisted of 30 NF, 39 EF, 9 SS, 109 CBD and 55 Trop (Table 1).

**Table 1:** Diversity statistics computed using 29911 Panzea SNP markers over the whole panel and five main genetic groups.

Group	Abb^a^	N_q_ b	N_q_>0.8^c^	Mean Sim^d^	*H_E_* e	Polym^f^
Northern Flint	NF	57	30	0.73	0.276	0.84
European Flint	EF	62	39	0.70	0.299	0.89
Iowa Stiff Stalk	SS	26	9	0.80	0.214	0.52
Corn Belt Dent	CBD	115	109	0.65	0.349	0.98
Tropical	Trop	76	55	0.67	0.339	0.98
Whole panel	S1P9	336	242	0.64	0.36	-

a Abb: abbreviation describing the group,

b N_q_ = ∑_i_ δ_iq_ with i the index of lines and q the index of groups, δ_iq_ the assignment proportion of line i to group q according to STRUCTURE software [40], [41] using 55 SSRs, Nq the number of lines in group q;

c number of lines assigned to group q with a genome proportion above 0.8;

d mean similarity within each group, calculated with IBS (identity by state);

e expected heterozygosity inside each group computed with r-Hierfstat [46];

f percentage of polymorphic loci within each group,

d,e,f statistics were computed considering lines with assignment to one group above 0.8.

### Molecular markers

The panel of maize lines was described using two different sets of SSR markers. The first one was described in [30]. It was composed of 54 tri-nucleotidic and 1 di-nucleotidic SSRs. The second one, reported here for the first time, consisted of 49 additional di-nucloetidic SSR markers. Di-nucleotidic SSRs have a higher mutation rate than tri-nucleotidic SSRs [35]. The panel was also genotyped with 57838 SNP markers synthesized for Illumina Golden Gate. Among them, 56110 (97%) markers passed the bead representation and decoding quality metrics, 49585 (88%) passed the analytical phase and could be scored with GenomeStudio v2009 software [31]. Then 45747 (92%) of these were polymorphic in the panel of 336 lines with less than 20% missing data and 15% heterozygosity and 45615 (99.7%) were not redundant according to the probe sequences. Among those, 43589 (95%) had a minor allele frequency (MAF) above 0.05. Finally 43224 (95% of polymorphic markers) non-redundant with MAF>0.05 were physically mapped on version 2 of the B73 genome sequence (called RefGen_v2) and were then used for linkage disequilibrium (LD) and association analyses. A subset of 29911 markers (65% of polymorphic markers, not always physically mapped) designed from 27 diverse founder lines called the Panzea diversity panel [36], non-redundant and potentially rare (MAF>0.01), were used for the diversity statistical calculation. Most of the additional markers were designed for mapping in B73×Mo17 populations [31]. They were considered for computation of diversity indexes in comparison with Panzea SNP information and association genetics. Additional markers not belonging to these two main sets were from diverse origins and considered only for association genetics investigations.

As a reference, we considered 535 SNPs discovered on regions that were fully sequenced in the same panel around *Vgt1* on chromosome 8 [21], ZmCCT on chromosome 10 [32] and Tb1-*D8* on chromosome 1 [30] in order to compare the MAF distributions.

### Similarity and Structure matrices

#### Similarity

We computed two different similarity matrices between lines (kinship), one considering the identity by state (IBS) with r-Emma [37] and the second considering the identity by descent (IBD), estimated following [38], taking the allele frequency into account with Cocoa software [39]. These were applied to the three different sets of markers (55 SSRs, 94 SSRs and 29911 SNP Panzea markers). We compared the correlation between the three sets of markers, while removing the diagonals of the matrices.

#### Structure

Population structure was investigated in order to define suitable covariates for association genetics models and investigate genetic diversity trends among genetic groups. We thus used two different sets of SSR markers (55 SSRs and 94 SSRs) and STRUCTURE [40], [41] software. We considered that lines were haploids and replaced heterozygous genotypes by missing data. We assumed a single domestication event and restricted our analysis to the correlated frequency model [41]. We set other parameters at their default values using the admixture model and infer ALPHA option. We used a 104 burn-in period and 10^6^ iterations. Allele frequencies in each of the K clusters (from 2 to 15) were estimated, and the percentage of genome derived from each cluster was estimated for each accession.

The structure matrix built with 29911 Panzea SNP markers was estimated by ADMIXTURE, which computes maximum likelihood estimations of individual ancestries from multilocus biallelic genotype datasets using the same statistical model as STRUCTURE with a very fast numerical optimization algorithm [42].

### Diversity levels revealed by the 50K Illumina array within and among genetic groups

Diversity parameters were estimated for the total panel and within each of the five main genetic groups determined from 55 SSRs [30], considering only 242 non-admixed lines (assigned to the different groups with a threshold of >0.8) and the set of 29911 Panzea markers. The minor allele frequency (MAF), observed (*H_O_*), expected (*H_E_*) heterozygosity, differentiation (*F_ST_*) corresponding to the ratio of inter-group diversity over the total genetic diversity according to Nei [43], [44] were calculated for each locus and overall loci at the group and panel levels. Bootstrap confidence interval (over loci) for pairwise genetic group *F_ST_* [45] were calculated with one hundred permutations. All statistics were computed with r-Hierfstat [46].

Finally, we considered the classification based on 55 SSRs as a reference to estimate SNP allelic frequencies in each genetic group, as,
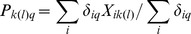
Where, 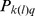 is the frequency of allele *k* at locus *l* in group *q*, 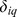 is the assignment proportion of line *i* to group *q*, 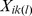 is the presence or absence of allele *k* at locus *l* for line *I*, coded as 0 and 1, respectively.

They were calculated for all lines using only lines with assignment to one group above 0.8.

### Detection of loci presenting a selection signature

When taking the structure obtained with 55 SSRs as the reference, we identified loci under selection according to differences in allele frequencies between genetic groups using BayeScan [47]. This program simulates correlations of allele frequencies among groups on the basis of the multinomial-Dirichlet likelihood [48]. The relative differentiation of a given population (genetic group in our case) at a given locus (*F_STB_*) is decomposed into a population-specific component (beta) shared by all loci and a locus-specific component (alpha) shared by all populations using a logistic regression. Departure from neutrality at a given locus is assumed when the locus-specific component is necessary to explain the observed diversity pattern (alpha significantly different from 0). A positive alpha value suggests diversifying selection, whereas negative values suggest balancing or purifying selection.

BayeScan uses posterior odds (PO) instead of Bayes factors to make decisions about the chance that each locus is under selection. The ratio of posterior probabilities is 
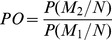
, *M_1_* and *M_2_* being the models without and with selection and *N* the number of loci tested. It copes with unequal population sizes and posterior probabilities allow control of the false discovery rate (FDR), *i.e.* the expected proportion of false positives among outlier markers. We used Jeffrey's scale of evidence [49] with posterior odds and defined selected loci as markers having log*_10_(PO)*>1. These values were highly correlated to *Q*-values, which are the minimum FDR at which the loci may be deemed significant.

### Linkage disequilibrium analysis

Linkage disequilibrium (LD) was first calculated as the squared correlation between allelic doses at two loci (*r^2^* using Plink [50]. As the presence of individuals from different genetic origins within the panel produces LD between unlinked loci (long-range LD), simply because of differences in allele frequencies all along the genome, this measure may lead to underestimation of the number of markers needed for whole genome association genetics scans. We therefore also estimated the *r^2^_s_* measure developed by [34] in r-LDcorSV that corrects LD for structure effects and can be directly linked to the power of the association tests obtained with models that include structure. We therefore used the structure matrix calculated with STRUCTURE and 55 SSRs as input.

The LD curve of both statistics (*r^2^* and *r^2^_s_*) according to the increase in physical distance was modeled with two non-linear regression models [51] according to [52] and [53] using the r-Nls package. The LD decay for each chromosome was obtained as the abscissa of the intersection between the LD decay curve and the horizontal lines y = 0.1 and 0.3. We compared these results with a sliding window approach (20 kb). This analysis was performed for all chromosomes together and each chromosome separately.

Each SNP was assigned to 20 haplotype clusters according to [54]. The rate of imputation error was minimal (3.5%) for this number of clusters. Individual chromosomes were regrouped locally using the multipoint linkage disequilibrium model of FastPHASE [55]. This model allows cluster memberships to change along the chromosome according to a hidden Markov model. For each SNP, we estimated the number of lines sharing each of the five major haplotypes and represented the probability of haplotype switch from one SNP to another along with the genetic recombination rate.

For all diversity indexes, we used a sliding window of 1 Mb and steps of 500 kb to visualize regions that underwent specific evolutionary events/selection leading to different allele frequencies in different groups and eventually higher LD.

### Phenotypic data

The whole panel was tested at three different locations (Germany_Einbeck: 52°N, 10°E, France_Gif-sur-Yvette: 49°N, 2°E, Saint-Martin-de-Hinx: 43°N, 1.3°W). The two latest groups of lines were also evaluated at France_Mauguio (44°N, 4°E). French locations were evaluated over 3 years (2002–2004) and the Einbeck location over 1 year (2005). Note that only 2002 data were considered in [30].

Lines were repeated twice at each location using a complete block design. In order to limit competition effects, each block was organized into four sub-blocks corresponding to earliness groups based on *a priori* information. Each individual plot consisted of a row of 15 plants planted at a density of approximately six plants per square meter.

Days to anthesis for male flowering (MFLW) and days to silking for female flowering (FFLW) and anthesis to silking interval (ASI) were measured in thermal time (GDD: growing degree-days) according to [56], with parameter values (Tb = 8° and *To* = 30°C) that maximized correlations between sites (MFLW8, FFLW8, ASI8).

A global ANOVA of the data was performed to test the genotype, location and genotype-by-location interaction significance. For association analyses, considering that the genotype-by-location interaction was low compared to the genotype effect, we estimated the adjusted mean of each genotype in the total trial network.

We fitted the mixed model
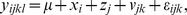
where *y_ijkl_* was the phenotype of the *l* individual of the *i* inbred line, in the *j* field trial, in the *k* subplot. *μ* indicates the mean. Inbred lines (*x_i_*) were considered as fixed effects. Field trial (*z_j_*) and subplot (*v_jk_*) were considered as random effects. *ε_ijk_* was the residual error. For each trait, LSMEANS of each inbred line *i* was calculated with SAS PROC GLM as 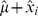.

### Whole genome association genetics

To investigate the effect of population structure, the proportion of genetic variance of each trait explained by the first four columns of the five group Q matrix obtained with 55 SSRs [30] was calculated with a linear model that did not take relatedness into account. We estimated group adjusted means as the predicted values of hypothetic pure lines that would be assigned 100% to each of the five groups.

As the structure of a population and/or the relatedness within a sample can increase the long-range LD, and consequently the rate of false positives, we corrected tests using a linear mixed model using the approach described by [57]. We considered two different four-column ancestry matrices obtained with STRUCTURE [40], [41] and 55 SSRs (called Q_55SSRs_), or ADMIXTURE [42] and 29911 SNPs (called Q_30KSNPs_). We compared the results obtained with four different kinship matrices obtained with 94 SSRs, using (1) identity by state (IBS) similarity obtained with the Rogers index [58] (called K_IBS(94SSRs)_) and (2) the normalized IBS index (called K_NORM.IBS(94SSRs)_ [59]) or (3) identity by descent (IBD) calculated with the Loiselle index [38] (called K_IBD(94SSRs)_). We also tested the kinship (4) obtained with 29911 SNPs and IBS (called K_IBS(30KSNPs)_).

We considered at each SNP the association model:
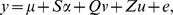
where *y* is the vector of phenotypes, *μ* is the mean, *S* the vector of individual genotypes, α the SNP fixed effect, Q the matrix of assignment of each line to each genetic group, *v* the vector of genetic groups fixed effects, *Z* the matrix of line occurrences, *u* the vector of line genetic background effects and *e* the vector of residuals. *Var(u) = 2KVg*, where *Vg* is the genetic variance and K is a matrix of similarity between lines.

We took the Q_55SSRs_+K_IBS(94SSRs)_ model as the reference to avoid having to use SNP as candidate loci and for population structure and kinship estimations. We compared *P*-values as obtained with the naive model (only the marker was included in the model) and those obtained with Q_55SSRs_, K_IBS(94SSRs)_, Q_55SSRs_+K_IBS(94SSRs)_, Q_30KSNPs_+K_IBS(94SSRs)_, Q_55SSRs_+K_IBD(94SSRs)_, Q_55SSRs_+K_IBS.NORM(94SSRs)_, K_IBS(30KSNPs)_, Q_55SSRs_+K_IBS(30KSNPs)_ models.

We then considered associations obtained within the three main groups (dents, flints, tropicals) obtained with STRUCTURE and 55 SSRs, using the corresponding K obtained with 94 SSRs in the linear mixed model.

In addition to these single locus models we used two complementary multi-locus models. First, since *Vgt1* was shown to be a major QTL involved in flowering time variation [21], [24], we used a second model that includes Vgt1 as a supplementary fixed effect in the Q_55SSRs_+K_IBS(94SSRs)_ model to further test the significance of SNPs that may be involved in flowering. Second, a forward variable selection was applied to the 96 markers with P-value<10^−5^, using the same mixed linear model used above (Q_55SSRs_+K_IBS(94SSRs)_). In order not to eliminate an increasing number of lines during the procedure, we used genotypes imputed with FastPHASE. At each step, a marker i chosen among 96-i+1 markers was added in the model. The relative quality of the statistical models was estimated for all data sets with the Akaike Information Criterion (AIC) that penalizes the likelihood by the increasing number of parameters to estimate (number of structure covariates + number of markers + polygenic variance + residual variance). The marker i added at each step was chosen to minimize this criterion. We stopped the procedure when the AIC criterion stabilized and the last marker added was not significant conditionally to the n–i markers included before in the model. All models were analyzed using r-Asreml [60]. Wald tests of fixed effects were based on variance estimates using the restricted maximum likelihood (REML) method and denominator degrees of freedom approximated by the method of Kenward and Roger [61]. We dealt with the multiple testing problem by applying both Bonferroni [62] and FDR approaches for P-values implemented in r-Fdrtool [63], [64]. The proportion of genetic variance explained by significant SNPs was computed based on the relative reduction in polygenic variance when the SNPs were added to the linear mixed model [65]. We compared these values with the proportion of genetic variance obtained with a linear model that includes structure only.

Genes located in the vicinity of QTLs or regions presenting non-neutral patterns were identified according to maize annotation version 2 (maizegenome.org).

Centromeric regions were consensually defined from maize GDB (maizegdb.org) and [66] flanking markers. Mega Blast of primers was performed on the B73 maize RefGen_v2 sequence.

## Results

### Genotyping

The SNP genotyping reproducibility was assessed with 20 DNA replicates and was above 0.999. The mean interval between successive markers was 50 kb (Table S1 in Text S1). Markers were relatively evenly distributed along chromosomes. However, one gap above 2 Mb was observed on the long arm of chromosome 1 (184908147 bp) and another one above 6.5 Mb on the small arm of chromosome 6 (9501960 bp) (Figure S1 and Figure S2 in Text S1).

### Polymorphism and MAF distribution

Among the 43224 polymorphic SNP markers or the 29911 Panzea SNPs (see Material and Methods for marker sampling details), 4% displayed rare alleles (MAF<0.05). The distribution of MAF for the 29911 Panzea SNPs showed a deficit in rare alleles (MAF<0.1) compared to other frequency classes (Figure S3 in Text S1). In order to compare polymorphism within the different genetic groups defined by Camus-Kulandaivelu *et al.* [30] without eliminating markers which may be monomorphic among non-admixed lines, we considered all 336 lines and calculated allele frequencies in each group on the basis of the quantitative assignments. The deficit in rare alleles was visible for tropicals (Trop), Corn Belt Dents (CBD), Stiff Stalk (SS) and, to a lesser extent, Northern and European Flints (NF and EF) (Figure S3 in Text S1). When considering only 242 non-admixed lines, 98% of the markers were polymorphic in Trop and CBD, only 84 and 89% were polymorphic in NF and EF, and 52% in SS (Table 1). Polymorphic rates were significantly different between groups (All pairwise Chi-squared tests with P-value<10^−16^). The observed heterozygosity was low (0.036), as expected for inbred lines. The average genetic diversity of the panel was 0.36. A lower genetic diversity value was obtained in NF (0.28) and EF (0.30) genetic groups relative to CBD (0.34) and Trop (0.33). The Wilcoxon Signed-Rank Test that does not assume normal distribution [67] showed that the pairwise within-group per-loci diversity distributions are significantly different, also when compared to the global diversity of the panel (P-values<10^−15^).

Among the 987 markers which were monomorphic in tropicals, 921 (93%) presented a different allele in dents, 610 (62%) in flints, with 589 (57%) being common to dents and flints. Considering the 3346 alleles that were rare (<0.05) in tropicals, 30% were lost in NF and EF, 57% in SS and 6% in CBD. Looking at the frequency of these alleles that were rare in tropicals (Figure S4 in Text S1), we observed that some of them increased in frequency until near fixation in flints and SS, but none reached a frequency of higher than 0.6 in CBD.

### Comparison of similarity matrices and structure with three different sets of markers

Similarities between lines obtained with Panzea and non-Panzea SNP markers were not linearly correlated (Figure S5 in Text S1). Similarities between one dent line and any other line were underestimated with non-Panzea markers relative to Panzea markers. This underestimation was particularly marked for similarities between CBD and SS dent lines. We therefore kept only Panzea SNPs for the diversity statistics calculation. The average IBS calculated with Panzea SNPs was higher in SS (0.80) and flints (NF: 0.73, EF: 0.70). Average similarities were 0.65 and 0.67 within tropicals and CBD, respectively, and 0.64 within the entire panel. When comparing IBS and IBD (Loiselle similarity [38],) obtained with different sets of SSR and SNP markers, we observed (Figure S6 in Text S1) different ranges of similarities: 0.2 to 1 with SSRs and IBS, 0.5 to 1 with SNPs and IBS, −0.4 to 1.2 for SSRs and IBD and −0.2 to 1.5 for SNPs and IBD. Correlations were high between IBS and IBD for 55 SSRs (R*^2^* = 0.77), 94 SSRs (*R^2^* = 0.77) and 30K SNPs (*R^2^* = 0.81). IBS similarities calculated with 30K SNPs were more correlated with IBS calculated with 94 SSRs (*R^2^* = 0.32) than with IBS calculated with 55 SSRs (*R^2^* = 0.29) (Figure S7 in Text S1). When tested with a Mantel test, all correlations were significant with *P*-value below 10^−16^. Among the IBS similarity indexes, variation was higher with 94 SSRs. As expected, variation was higher with the IBD index which standardizes with diversity. Differentiation between groups was also consistent between SNPs and SSRs, with the largest differentiation being observed between NF and SS (Table S2 in Text S1).

Group assignments were consistent between 55 SSRs and 29911 Panzea SNPs for structure levels ranging from 2 to 5 (Figure S8 in Text S1). Groups obtained with 94 SSRs sometimes differed. For instance, tropicals clustered with flints instead of dents at Q = 2, European and Northern Flints separated at Q = 9 instead of Q = 5 with 55 SSRs or Panzea SNPs. Admixture levels were slightly higher for Panzea SNPs (Figure S9 in Text S1), with mean assignment to the main genetic group of 0.82 on average for 55 SSRs or 0.80 for 94 SSRs (not significantly different according to Wilcoxon Signed-Rank Test considering sampling of individuals, *P*-value = 0.24), 0.70 for 29911 Panzea SNPs (distribution significantly different from SSRs, *P*-value<10^−16^).

### Identification of loci under selection

For SNPs, Hierfstat *F_ST_* (*F_ST_*) based on non-admixed lines (assignment to groups >0.8) ranged from 0 to 0.95. According to Hierfstat, 86% of F_ST_ values were significant (P-value<0.05, 1000 permutations) and 5% were above 0.42 (Figure S10 in Text S1, see Figures S11 and S12 for visualization along the genome). According to BayeScan (Figure S13 in Text S1), 91 markers had a *log_10_(PO)* above 0, including 34 substantially significant (ranging from 0.5 to 1) and 18 highly significant (ranging from 1 to 3.1). All of these markers had a positive alpha value corresponding to diversifying selection. Their *F_ST_* values ranged from 0.36 to 0.72 when calculated with Hierfstat and from 0.38 to 0.51 when calculated with BayeScan. Therefore, only *F_ST_* found within the range 0.4–0.6 when computed with BayesScan (*F_STB_*) were detected as outliers. Figure S14 in Text S1 illustrated that *F_ST_* and *F_STB_* are constrained in a different way by MAF, with only loci with MAF above 0.3 being detected as outliers (*log_10_(PO)*>1) according to BayeScan.

### Extent of LD and estimation of the number of markers needed for association genetics

**Figure 1:**
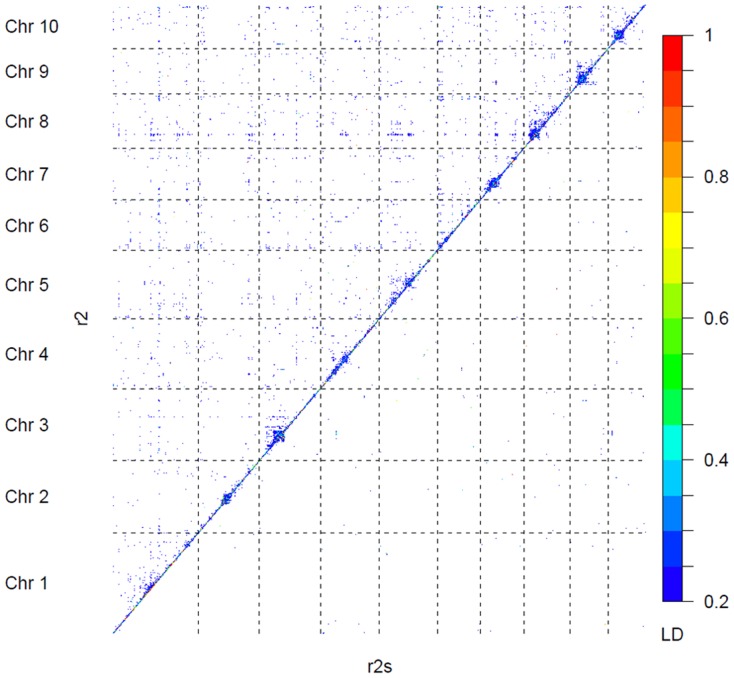
Genome-wide linkage disequilibrium between all loci within and between chromosomes. The upper triangle corresponds to the LD *r^2^* measure calculated with Plink [50], the lower triangle to the LD r*^2^_s_* measure [34] corresponding to r*^2^* corrected for structure, using the Q matrix obtained with STRUCTURE software [40], [41] and 55 SSRs. Values above 0.2 are highlighted by colored dots.

On average, over the entire genome, LD decreased below *r^2^* = 0.1 after 200 kb according to the Hill and Weir model [53] (Table 2). This estimation was 160 kb when considering average LD by class of 20 kb, and 6400 bp when using the Sved model [52] (Table S3 in Text S1). According to the Hill and Weir model, LD ranged from 100 to 300 kb depending on the chromosome (Table 2). It was generally low for markers that were on different chromosomes with a 95 quantile pairwise *r^2^* (1 billion values) of 10^−5^. Some long distance LD was nevertheless observed between centromeric regions (see Table S4 in Text S1 for positions), for chromosomes 1, 5 and 8 in particular (Figure 1).

**Table 2:** Extent of linkage disequilibrium and number of markers needed to reach average *r^2^* = 0.1 for individual chromosomes and the whole genome.

Chromosome	*r^2^* extent (kb)^a^	*r^2^_s_* extent (kb)^a^	#Markers^b^
1	111.11 (2711)	78.61 (3832)	6892
2	230.50 (1028)	183.25 (1293)	4963
3	191.37 (1213)	146.75 (1582)	4938
4	286.24 (843)	233.92 (1031)	4784
5	144.89 (1503)	112.97 (1927)	4711
6	156.89 (1078)	121.36 (1393)	3456
7	199.92 (881)	154.89 (1137)	3562
8	303.90 (578)	211.58 (831)	3719
9	228.62 (685)	176.52 (887)	3129
10	289.97 (518)	237.90 (631)	3070
Whole genome	197.75 (10402)	152.37 (13500)	43224

LD extent was computed with 43224 SNPs having MAF>0.05.

a Physical distance to reach *r^2^* or *r^2^_s_* equal to 0.1 estimated using the non-linear regression implemented in r-nls obtained by fitting Hill and Weir model [53]. r*^2^* and *r^2^_s_* obtained with Plink software [50] and r-LDcorSV [34], respectively. The number reported in brackets indicates the number of equidistant markers that would be needed to reach an average r*^2^* of 0.1 between adjacent markers;

b number of markers used to estimate LD extent for each chromosome and the whole genome.

Extracting the variation due to population structure in LD measures (see Material and Methods for details) led to a decrease in *r^2^s* relative to *r^2^* (Figure S15 and Figure S16 both in Text S1). In contrast to *r^2^*, *r^2^_s_* values above 0.2 were obtained almost exclusively for physically linked markers (Figure 1). This led to an estimate of LD decay of 150 kb, *i.e.* a correction of 23% (Table 2). Such corrections were particularly marked in some centromeric regions (e.g. chromosome 8). We nevertheless detected some regions where *r^2^_s_* corrected by structure was higher than *r^2^* (Figure S11), and some markers on different chromosomes that were in LD even after correction for structure.

### Diversity, LD and differentiation trends along chromosomes

Based on LD analysis, we plotted all diversity and LD statistics using a sliding window of 1 Mb by steps of 500 kb. Centromeric regions showed specific patterns (Figure 1, Figure S11 and S12), with a general trend towards higher LD (0.39 compared to 0.14 outside centromeres), especially on chromosomes 1, 3 and 8 (0.45, 0.44 and 0.69, respectively). The highest local LD outside centromeric regions was found for a region between *Su1* and *Bt2* on chromosome 4 (between 40 and 60 Mb).

When assigning each line to 20 estimated clusters for each SNP position using FastPHASE algorithm [55], we found that the probability of cluster switch at each SNP was lower in centromeric regions (0.02 compared to 0.04 genome wide, Figure S17 in Text S1), in accord with the recombination rate calculated with genetic maps (Figure S11). The percentage of lines carrying the major haplotype increased to 67% in centromeric regions compared to 53% genome wide.

Total diversity was higher in centromeric regions (Figure S17 in Text S1). Diversity within different genetic groups was slightly lower in centromeric regions (0.26 compared to 0.30 genome wide) and systematically for flints (0.21 for NF and 0.26 for EF) (Figure S17 in Text S1). This pattern was particularly clear on chromosomes 1, 2, 4, 6 and 8. Centromeric regions of these chromosomes also presented high relative differentiation among groups (*F_ST_*) (0.31, 0.21, 0.25 compared to 0.16 genome wide). For other chromosomes, centromeres did not display a specific differentiation pattern.

### Phenotypic variation for male (MFLW), female flowering time (FFLW), anthesis to silking interval (ASI) and association genetics

The analysis of the present design included eight additional field experiments compared to our previous studies which included only two experiments [21], [30]. Plot heritability was 0.96, 0.97 and 0.45 for FFLW8, MFLW8 and ASI8, respectively. It confirmed high phenotypic variation for FFLW8 (sd = 168 GDD or 13.6 days), MFLW8 (*sd* = 161.5 GDD or 12.9 days), ASI8 (*sd* = 26 GDD or 2.2 days). This variation appeared to be closely related to the population structure (*R^2^* = 0.51, 0.54 and 0.11 for FFLW8, MFLW8 and ASI8, respectively). For FFLW8, the group adjusted means were 762 (±41), 763 (±35), 943 (±73), 889 (±23) and 1181 (±33) for NF, EF, SS, CBD and Trop, respectively (see Table S5 in Text S1 for other traits).We tested several structure and kinship matrices for FFLW8 association studies in order to avoid an excess of false positives. Figure S18 in Text S1 illustrates the logarithm of cumulative *P*-values obtained. The stronger correction was obtained with the model involving the kinship matrix calculated with 29911 Panzea SNPs. Adding a structure matrix did not change the *P*-values in that case. The models involving structure matrix calculated with 55 SSRs and either IBS or IBD kinship calculated with 94 SSRs were equivalent. Considering the limited differences between SSR and SNP based estimates of population structure and kinship, we used the model Q_55SSRs_+K_IBS(94SSRs)_ as reference to avoid using SNPs as candidate loci and for population structure and kinship estimations. As illustrated by Figure S18 in Text S1, *P*-values obtained with model Q_55SSRs_+K_IBS(30KSNPs)_ were globally higher than with Q_55SSRs_+K_IBS(94SSRs)_. However, most associations significant with *P*-values<10^−5^ obtained with model Q_55SSRs_+K_IBS(94SSRs)_ remained highly significant (*P*-values<10^−3^) with model Q_55SSRs_+K_IBS(30KSNPs)_. *P*-values of the two models were globally strongly related, the most notable exception being the centromeric region of chromosome 8, for which markers with a *P*-value of 10^−7^ Q_55SSRs_+K_IBS(94SSRs)_ were never below 10^−3^ with Q_55SSRs_+K_IBS(30KSNPs)_. This probably relates to the large number of markers in high LD in this region, which consequently have an important contribution to the K_IBS(30KSNPs)_ kinship estimation (Rincent et al., pers. com.).

**Figure 2:**
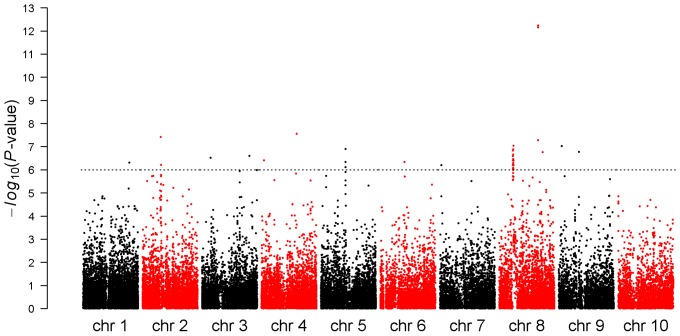
Manhattan plot for female flowering (FFLW8) associations across the whole genome. *P*-values were obtained with the mixed model including the structure matrix obtained using STRUCTURE software and 55 SSRs and the kinship matrix obtained with 94 SSRs and IBS measure. Horizontal dashed line indicates Bonferroni-corrected 5% significance threshold.

**Figure 3:**
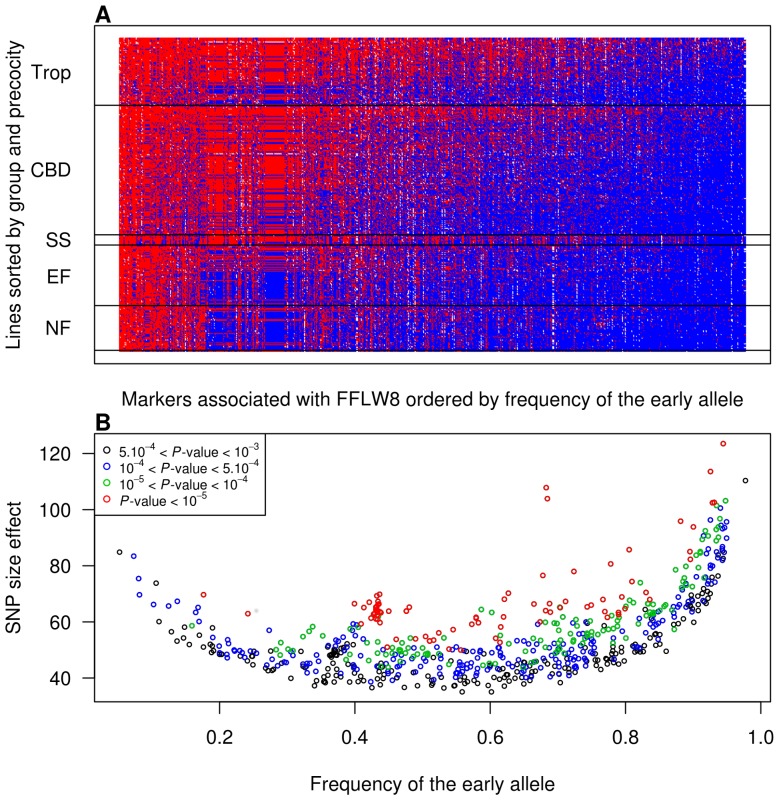
Distribution of SNP effects (GDD) according to the early allele frequency. In this figure, 673 markers with *P*-value<10^−3^ are represented. (A) For all inbred lines (rows) and SNPs (columns), red and blue colors correspond to the presence of late and early alleles, respectively. (B) The absolute SNP effect versus the frequency of the early allele for significant associations. Different colors correspond to different *P*-value thresholds.

The association statistics obtained for FFLW8 are summarized in Figure 2. The FDR 5% corresponded to *P*-value<10^−3^. Considering this threshold, 673 markers were associated with FFLW8, 843 with MFLW8 and 145 with ASI8. Among associations for FFLW8, 96 (corresponding to 18 regions) had a *P*-value below 10^−5^, 50 below 10^−6^ (corresponding to Bonferroni correction) and 7 below 10^−7^ (corresponding to a break point in the *P*-value distribution, Figure S18 in Text S1). These two latter categories corresponded to 14 and 5 regions, respectively. Considering MFLW8, 108 markers had *P*-values below 10^−5^, including 77 in common with FFLW8. For ASI8, seven markers had *P*-values below 10^−5^ and were not shared with FFLW8 or MFLW8 (Table S6 and Table S7 in Text S1). When adding *Vgt1* (presence-absence of the *Mite* allele), which has a strong effect on flowering time (7 days) according to [21], as a covariate in the model, all markers remained significant for FFLW8 (results not shown). The estimated effects of markers having P-value<10^−5^ ranged from 44 (3.5 days) to 123.5 GDD (9.9 days), with a majority of small effects and a few strong effects (Figure S19 in Text S1). The bottom envelope in Figure 3 illustrates that loci with low MAF necessitate greater absolute effects to pass the significance threshold. We also observed that for the significant tests the early allele generally had the highest frequency and that the highest absolute effects corresponded to extreme frequencies (Figure 3). Estimated genetic variances associated with loci with significant effects ranged from 0 (after discarding a limited number of low negative values) to 34%, with three-quarters of the values below 10% (Figure S20 in Text S1) according to the linear mixed model (lmm) and from 0 to 9% according to the linear model (lm). The proportion of early alleles in one line was negatively correlated (−0.84) with FFLW8 and therefore positively correlated with its precocity (Figure S21 in Text S1). This analysis was complemented by a multi-locus analysis of FFLW8 starting of the 96 markers with significant individual effects (*P*-value<10^−5^). The first marker included was *ZCN8* (#23) as expected. The nine first markers added in the model were significant with (*P*-value<10^−3^) conditionally to the previous model. The last marker entering the model with (*P*-value<0.05) was the 29^th^. AIC then continued to decrease until 71 markers were included in the model (see Table S8 for the order of inclusion of markers). The polygenic variance explained, once structure effect was removed, was 62% when considering the 29 first markers and 66% with 71 markers.

Finally, to avoid possible confusion between population structure and marker effects, we estimated marker effects with an ANOVA in three different genetic groups (flints, dents and tropicals). For the 96 markers with *P*-value<10^−5^ for FFLW8 in the entire panel and lmm, marker effects were higher in tropicals (median = 162 GDD) compared to dents (75 GDD) and flints (53 GDD). Among them, 70 were significant in tropicals, 34 in dents, 6 in flints (*P*-value<10^−3^). All significant effects had the same sign in the three genetic groups. In addition, 110 markers that were not significant in the entire panel were highly significant (*P*-value<10^−6^) in at least one group, 9 in flints, 63 in dents and 39 in tropicals, with a mean effect of 80, 120 and 230 GDD, respectively.

### Regions with the highest contribution to flowering time variation

Table 3 summarizes information on the 18 most significant loci selected among cluster of loci along the genome and showing major contributions to flowering time variation according to models Q_55SSRs_+K_IBS(94SSRs)_ and Q_55SSRs_+K_IBS(30KSNPs),_ as well as 10 additional loci strongly under selection. Regarding the first aspect, the marker that explained the highest genetic variance (24 and 9% for lmm and lm, respectively) with the highest *P*-value (10^−13^, 10^−12^, 10^−4^ for FFLW8, MFLW8 and ASI8, respectively) was located on chromosome 8 (#23 in Table 3, 123506141 bp), 5000 bp from the *Zea CENTRORADIALIS* gene *ZCN8* (GRMZM2G179264, 123501085 bp). There was no other marker in the 50K Illumina array closer to this gene. Its effect was 108 GDD (9.7 days). It was 1 Mb apart from PZB02155.1 (124657056 bp), which was found to be associated with flowering time in the NAM population [4] but not in this panel when including structure in the model. It was 8 Mb apart from Vgt1 *Mite* (131984851 bp), a major flowering time QTL that has been cloned [24]. Vgt1 *Mite* presented an association *P*-value<10^−14^ with SAS GLM [32] and P-value<10^−9^ with lmm in this study. LD (*r^2^*) between *ZCN8* (marker #25) and *Vgt1 Mite* was 0.3. This marker remained significant when adding *Vgt1 Mite* in the linear mixed model. *ZCN8* was located between two flowering time meta QTL defined in the meta analysis of [22] (green meta QTL Vgt2 and blue meta QTL *Vgt1* in Figure 3 of the above paper), between marker pdc1 (118167604 bp) and marker umc1592 (125903155 bp), at the border of the *Vgt1* QTL.

**Table 3:** Regions associated with FFLW8 (*P*-value below 10^−5)^) and/or under selection (*log_10_(PO*) above 0.5).

Nb	Marker Name^a^	Cent^b^	Chr^c^	Pos^d^	*P*-value^e^	Effect^f^	*P*-value nc^g^	*R^2^* lm^h^	*R^2^* lmm^i^	PO j	*F_ST_* k	Meta QTL^l^	Gene ID^m^	Gene Descr^n^	Gene Dist (bp)^o^
1	PZE-101106079	0	1	109 394 965	1.39E-01	−19.41	1.00E-16	0.02	0.07	0.77	0.51	1_5	GRMZM2G041613	uncharacterized	0
2	SYN13483	0	1	249 324 652	6.64E-06	−82.29	1.05E-05	0.03	0.11	−1.69	0.00	1_10	GRMZM2G103843	ribokinase activity	0
3	SYN19258	0	1	278 034 859	7.75E-01	−3.98	4.56E-02	0.00	0.02	1.49	0.59	-	GRMZM2G328224	transferase activity on hexosyl groups	0
4	PZE-102079486	0	2	62 516 828	3.43E-06	−70.49	4.42E-12	0.04	0.08	−2.09	0.13	2_4	GRMZM2G084462	tRNA isopentenyltransferase activity	0
5	PZE-102086452	1	2	78 699 684	3.87E-08	76.55	1.00E-16	0.06	0.23	−1.62	0.34	2_4	GRMZM2G474153	protein kinase activity	0
6	SYN12061	0	2	199 457 622	7.10E-06	51.01	5.64E-07	0.04	0.09	−1.45	0.00	2_5	GRMZM2G400173	transporter activity	0
7	PZE-103099066	0	3	159 526 813	1.E-06	62.64	1.E-16	0.06	0.14	−2.22	0.16	-	GRMZM5G891247	enzyme inhibitor activity	47 249
8	PZE-103098863	0	3	159 170 263	6.48E-01	−7.92	7.53E-14	0.00	0.06	1.41	0.68	-	GRMZM2G082608	uncharacterized	0
9	PZE-103145047	0	3	199 953 486	2.49E-07	−61.64	1.00E-16	0.04	0.13	−2.08	0.37	-	GRMZM5G886883	uncharacterized	0
10	PZE-104010434	0	4	7 567 350	3.03E-01	14.95	3.56E-02	0.00	0.00	1.05	0.55	-	GRMZM2G012821	uncharacterized	49 496
11	PZE-104013311	0	4	11 576 332	3.94E-07	65.35	7.35E-05	0.05	0.17	−2.02	0.22	-	GRMZM2G113241	uncharacterized	50 532
12	PZE-104080388	0	4	154 689 801	2.77E-08	−85.74	3.33E-16	0.03	0.00	−1.92	0.20	-	GRMZM2G138407	transcription factor activity	106 768
13	PZE-105049333	0	5	40 625 928	3.77E-01	12.11	5.02E-01	0.01	0.02	1.14	0.58	5_4	GRMZM2G150893	peroxidase activity	119 669
14	PZE-105082545	0	5	97 872 011	1.28E-07	−66.39	5.43E-12	0.04	0.10	−1.75	0.50	-	GRMZM2G018962	exostosin-like protein	351 398
15	PZE-105130917	0	5	187 848 427	4.93E-06	−63.67	4.10E-13	0.03	0.07	−2.18	0.15	5_6	GRMZM2G159918	transferase activity on hexosyl groups	0
16	PZE-106032161	0	6	74 959 244	2.01E-06	59.17	1.11E-16	0.03	0.08	−2.15	0.34	6_1	GRMZM2G005499	transmembrane transport	0
17	SYN38078	0	6	158 225 752	4.43E-06	−61.61	5.16E-06	0.03	0.02	−2.00	0.17	6_3	GRMZM2G074792	intracellular zinc ion binding	0
18	SYN18613	0	7	150 876 445	5.64E-01	8.61	9.32E-01	0.00	0.00	3.10	0.67	-	GRMZM2G038449	uncharacterized	11 523
19	PZE-108038931	1	8	45 270 084	9.38E-08	−69.31	8.99E-15	0.03	0.03	−1.81	0.37	-	GRMZM2G142870	ATPase activity	0
20	PZE-108035509	1	8	52 084 948	3.05E-02	35.04	9.56E-09	0.01	0.12	2.85	0.61	-	GRMZM2G006585	zinc ion binding	5783
21	PZE-108053909	0	8	95 953 461	5.50E-01	8.45	6.87E-02	0.01	0.03	1.32	0.58	8_3	GRMZM2G074645	transcription factor activity	174 238
22	SYN12978	0	8	120 970 129	6.93E-01	5.07	1.00E-16	0.01	0.00	0.63	0.36	-	GRMZM2G473111	uncharacterized	0
23	PZE-108070380	0	8	123 506 141	5.88E-13	107.83	1.00E-16	0.09	0.24	3.10	0.71	-	GRMZM2G179264	ZCN8 protein	5056
24	PZE-108081330	0	8	138 262 446	2.E-07	81.15	1.E-16	0.06	0.34	−1.58	0.34	-	GRMZM2G164341	transcription factor activity	0
25	PZE-108089589	0	8	146 555 253	2.32E-01	−18.36	1.11E-03	0.00	0.00	1.02	0.58	-	GRMZM2G094165	carbonate dehydratase activity, binding	0
26	PZE-109008014	0	9	8 944 436	9.45E-08	67.29	7.12E-12	0.03	0.02	−2.07	0.20	-	GRMZM2G142072	transcription elongation regulator activity	0
27	PZD00049.4	0	9	17 019 800	2.E-06	72.63	1.E-16	0.04	0.10	−1.70	0.32	-	AC197699.3	transcription factor activity	0
28	PZE-109038470	0	9	57 186 011	1.70E-07	94.50	1E-16	0.04	0.17	−2.10	0.19	-	GRMZM2G076272	transcription factor activity	0

a Illumina marker name;

b if marker in centromeric region according to maize GDB and. [66] flanking markers, then 1, else 0;

c physical chromosome;

d physical position in RefGen_v2;

e marker *P*-value for female flowering in growing degree days base temperature 8 (FFLW8) according to model Q_55SSRs_+K_IBS(94SSRs)_;

f marker effect on FFLW8 according to model Q_55SSRs_+K_IBS(94SSRs)_;

g marker *P*-value for FFLW8 according to naïve model;

h percentage of variance explained by the marker in a linear model Q_55SSRs_;

i percentage of variance explained by the marker in a linear mixed model Q_55SSRs_+K_IBS(94SSRs)_;

j *log_10_*(posterior odds) for selection test obtained with BayeScan [47], significant (highly) when >0 (>1);

k differentiation index calculated with r-Hierfstat [46] using non-admixed lines;

l meta QTL name (adapted from [10]): meta QTL X_Y with X the chromosome number et Y the QTL number;

m name of closest annotated gene according to RefGen_v2;

n closest annotated gene function;

o distance between marker and closest annotated gene.

Other markers with a main contribution to FFLW8 were located on chromosomes 1 (#2), 2 (#5), 3 (#7,#9), 4 (#11), 5 (#14), 8 (#24), 9 (#27, #28), with a genetic variance ranging from 10 to 23% according to lmm and 3 to 6% according to lm. Ten additional loci displayed intermediary effects. According to the projection of flowering time meta QTL [10] on the maize sequence, seven associated markers were found in meta QTLs (#2, #4, #5, #6, #15, #16, #17). Five loci were next to one NAM [4] association (#9, #11, #15, #16, *ZCN8* #23). Note that some other markers with slightly lower *P*-values (10^−7^<*P*-value<10^−5^) were close to NAM associations [4] and were included in the meta QTLs [10] for the majority (#4, #6, #17, #21, #22). Several associated markers were close to ZCN genes and loci associated with flowering time in other studies (#4 5 Mb from *ZCN4*, 400 kb from NAM PHM3457.6, in meta QTL 2_4; #17, 200 kb from NAM PHM4748.16, 1 Mb from *ZCN7*, in meta QTL 6_3; #23 5000 bp from *ZCN8*, 1 Mb from NAM PZB02155.1; #16, in meta QTL 6_1, 1 Mb from *ZCN15*, 5 Mb from NAM PZA00355.2; #5: centromere of chromosome 2, 5 Mb from *ZCN21*, in meta QTL 2_4). Note that #15 may be involved in the sugar pathway as observed in Arabidopsis [68].

### LD, diversity and differentiation at markers associated with FFLW8 and other regions under selection

Diversity and *F_ST_* were higher at markers associated with FFLW8 (*P*-value<10^−5^) as compared to other parts of the genome (*H_E_* of 0.42 compared to 0.36, *F_ST_* of 0.27 compared to 0.16). Within group diversity (*H_S_*) was also lower in these regions than for the whole genome in NF (0.17) and EF (0.22) and in contrast higher in tropicals (0.4) (Figure S17 in Text S1, Figure S12). For these loci, the mean proportion of early alleles accumulated in NF, EF, SS, CBD and Trop non-admixed lines was 0.87, 0.83, 0.41, 0.48 and 0.39, respectively (Figure S22 in Text S1), consistent with the average flowering time of these groups. More balanced allele frequencies in dent groups was noted in particular on centromere of chromosome 2 (#5), chromosome 3 (#9), 4 (#11) and 8 (#23 close to *ZCN8*, see above). *ZCN8* (*Vgt2* locus) appeared to be strongly under diversifying selection (*log_10_(PO)* = 3.1). Allelic frequencies at *Vgt2* were slightly more differentiated than those observed for *Vgt1* (for *Vgt1* and *Vgt2*, the early allele was fixed in NF, frequencies were 0.94 and 0.97 in EF, 0.5 and 0.7 in CBD, 0.17 and 0.13 in tropicals, respectively). As shown in Figure S11, the region between *Vgt2* and *Vgt1* was characterized by low diversity in flints and a low recombination rate. Differentiation was high only around *Vgt2*.

Besides these diversity trends, regions associated with flowering time also displayed higher LD (*r^2^* = 0.2 compared to 0.14 for the entire genome) and lower recombination (0.02 compared to 0.04). The major haplotype in these regions was shared by 65% of lines (compared to 50% for the whole genome) (Figure S11).

In addition, some markers were found to be under selection although not related to flowering time in this panel. They nevertheless were related to flowering time according to other studies (#1, in meta QTL 1_5, 4 Mb from an association with *P*-value 10^−5^; #3 1 Mb from *Phyc1*; #18, 200 kb from NAM PZA02722.1. This suggests that the association mapping power at these loci may have been hindered by fixation in some of the groups.

## Discussion

### Suitability of the 50K Illumina array for investigating line similarities, panel structures and local diversity trends along the genome

Ascertainment bias due to the marker type and selection can be a concern in diversity analyses. We confirmed that considering an overrepresented set of B73 (SS)×Mo17 (CBD) SNPs lead to overestimated genetic distances for dent (CBD and SS) lines (Figure S5 in Text S1), as observed in other studies [31], [69]. When excluding these markers and considering only Panzea SNPs, we did not observe this trend and obtained population structure results that were highly consistent with those obtained with 55 SSRs, including mostly trinucleotides and more (Figure S8 in Text S1). The differentiation between groups was also consistent between SNPs and SSRs, with the greatest differentiation noted between NF and SS (Table S2 in Text S1). This illustrates that when based on a relevant marker sample, although not leading to the discovery of new polymorphisms as compared to other approaches like GBS [70] or sequencing [71], SNP arrays can be a powerful tool for investigating population structure.

The average genetic diversity of the panel calculated with Panzea SNPs was 0.36. This value was in the upper range of those reported by other diversity analyses based on SNP genotyping, ranging from 0.27 to 0.39 [72], [73], [74], [75], [76]. Only eight percent of markers appeared to be monomorphic, showing that some rare alleles discovered with the Panzea panel used to build the 50K Illumina array were not represented in our panel. Considering the MAF distribution, we observed a lower proportion of rare alleles (<0.1) compared to other frequency classes and a slight excess of intermediate alleles (Figure S3 in Text S1). This frequency profile clearly deviates from the L-shaped pattern (excess of rare alleles) expected at mutation drift equilibrium in the absence of bias in polymorphism discovery [77]. This could be the result of specific evolutionary processes and/or the experimental strategies used during marker assembly. The deficit in rare alleles may be due to some extent to SNP discovery, which was mostly based on 27 lines [36], [78] and followed by quality and polymorphism criteria in favor of balanced polymorphisms [31]. The slight excess in intermediate frequency alleles was consistent with the high enrichment in intermediate frequency alleles observed for three genomic regions that were sequenced (Figure S3 in Text S1). This could be related to allelic differentiation between groups due to drift and diversifying selection for some regions.

Beyond these trends for the global panel, we also noticed contrasted frequency patterns across genetic groups. The high deficit in rare alleles in tropicals was probably due to their under-representation (relative to their very high diversity) at the SNP discovery step. Other groups passed comparable sampling procedures, so the differences that were observed suggest the involvement of different evolutionary processes.

Fifteen percent of loci were monomorphic in flints, compared to 2% only in CBD and tropicals (Table 1). When looking at alleles that were rare in tropicals (<0.05), the difference was even more striking, with 30% of alleles lost in flints compared to only 6% in dents. In contrast, some alleles that were originally rare in tropicals reached near fixation in flints (NF and EF) (Figure S4 in Text S1). This suggests that the NF group passed severe bottleneck(s) when diverging from tropical germplasm approx. 1900 years ago [79], [80]. The relative (compared to other groups) overabundance of rare alleles observed for Northern Flints suggests that the population expansion that followed the differentiation of this group was accompanied by limited introgression by external genetic groups.

As stated above, the level of polymorphism observed in CBD presented only limited loss compared to tropical materials. Alleles that were observed in flints while being rare or absent in tropicals were also generally noted in dents (CBD), whereas the reverse was not true. This is consistent with CBD originating in part from flints (NF) and in part from a group relatively close to tropicals, approx. 400 ago [81]. The relative deficiency in rare alleles observed for CBD (Figure S3 in Text S1) confirmed that they emerged recently from a restricted number of founders from these two origins [82].

When comparing European flint diversity to that of Northern flints, we found that 93% of alleles were common to both groups, which was higher than in previous studies with multiallelic markers (75%) [83], [84]. Some alleles (5410) observed in EF were absent in NF. These alleles were generally also observed in non-admixed CBD (89%) or tropicals (94%). This confirms that EF is a recent group mainly derived from NF after their introduction into Europe and subsequent hybridization with materials of tropical origin, probably different from those used to form CBD [84]. Consistent with these trends, we observed a lower genetic diversity for NF (0.28) and EF (0.30) relative to CBD (0.35) and tropicals (0.34).

### Linkage disequilibrium and estimation of marker density required for association studies

The genotyping method we used did not allow us to estimate LD at the gene level as obtained by [85] but was adequate to reveal the LD structure at the genome level when considering a wide diversity of lines (tropicals, Corn Belt Dents, Northern and European Flints).

LD between unlinked or distant polymorphisms was globally low in the panel (quantile 95% of 10^−5^ between different chromosomes) with, however, notable exceptions between centromeric regions of chromosomes 1, 5, 8 and a few additional regions, like the extremity of chromosome 10 (Figure 1). Correction for the population structure (*r^2^_s_*) clearly removed long distance LD (Figure 1, Figure S15 in Text S1), especially LD among centromeres of different chromosomes. Some pairs of markers located on different chromosomes still presented *r^2^_s_* above 0.2. This could suggest that we did not perfectly correct the measure for the structure effect and that alternative approaches such as calculating *r^2^_vs_* [34] that corrects for relatedness should be considered. Alternatively, LD between unlinked loci may be due to loci that are involved in common functions and subjected to joint selection pressure.

For closely linked polymorphisms, we observed a rapid decrease in *r^2^* values as the physical distance between SNPs increased. The fitted LD values obtained with the non-linear model [53] and the average obtained by the distance class approach were close. This suggests that the Hill and Weir model [53], taking into account the sample size and mutation rate, was more realistic than the Sved model [52] which hypothesised that the size of the panel was equal to the effective population size, the panel had undergone no selection or mutations and the recombination rate was constant along the genome. According to the Hill and Weir model, LD dropped below 0.1 after 200 kb at the genome level and dropped to almost the background level at around 500 kb-1 Mb. This value was in the range of those reported in the literature for different types of panels [85], [86], [87], [88] and illustrates that linkage disequilibrium in maize has markedly increased as a result of domestication [1] and genetic improvement [2], [74], [89]. We observed that the extent of LD varied among chromosomes, *i.e.* 100 kb on average along chromosome 1, between 140 and 200 kb on average along chromosomes 5, 6, 3 and 7, between 200 and 300 kb along chromosomes 9, 2, 4, 10 and 8, which suggests different selection pressures were involved. Note that this ranking apparently varies among populations [74], [89]. Extracting the variation due to population structure did not much change these trends, with an average decrease of around 150 kb instead of 198 kb. We noticed that correction of r*^2^* by the *r^2^_s_* estimator was generally less marked than for *Vitis vinifera* [34]. This suggests that the maize genetic groups considered in our study were less differentiated than groups of grape varieties corresponding to different uses (table vs. wine production).

Visualization of LD as a sliding window (1 Mb) approach revealed high variation along chromosomes (Figure S11). It confirmed a general trend of higher LD in centromeres, as expected given the low recombination rate of these regions [31], [36]. This was especially noteworthy on chromosomes 7, 8 and 10. We noted that, according to LD and the diversity profiles, the position of the centromere of chromosome 5 obtained by [66] was probably more realistic than that recorded in MaizeGDB (maizegdb.org) (Figure S11, S12). With the FastPHASE algorithm, we observed one predominant ancestral haplotype shared by 67% of lines in centromeric regions, in contrast to the rest of the genome, which presented more balanced frequencies of ancestral haplotypes, with the predominant one being shared by 53% of lines. The probability of haplotype switch from one SNP to another computed with FastPHASE was also lower in these regions. Interpreting these probabilities as recombination rates could be questioned [55], but, overall, these results are consistent with the hypothesis that centromeres have undergone less recombination since domestication as compared to the rest of the genome. This could be due to mechanical obstacles to recombination and gene flow in the vicinity of centromeres and/or selection forces that preferentially occur in these regions [78], [90], [91], [92]. High differentiation rates, indicating selection forces, were indeed observed for centromeres of chromosomes 3, 5, 6 and 8 (F*_ST_* of 0.22, 0.21, 0.25, 0.31, respectively, compared to 0.16 on average on the genome). Differentiation at some centromeric regions was also observed by [1] in a panel that included wild relatives.

We detected peaks of LD outside centromeres that suggested different local histories of recombination [78] and highlighted some putative selective events involved in flowering time or other adaptive traits. The highest local LD was observed between *Su1* [93], [94], [95], [96], Tga1 [97] and Bt2 on chromosome 4, a region involved in carbohydrate metabolism [98], [99] known to have played a key role in maize domestication. Generally, a r*^2^* of 0.10 is considered to be the minimum LD value needed to detect associations with complex traits. This reasoning is based on a large effect QTL that explains 10% of phenotypic variation (*h^2^_q_* = 0.1), which is a rather high expectation according to present knowledge, as also discussed by Van Inghelandt *et al.* [89]. Detection of markers that explain 10% of polymorphism at such QTLs (r*^2^* = 0.1) and thus 1% (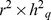) of the phenotypic variation could be considered as a minimum to have acceptable power with panel sizes classically used in plants (generally several hundred individuals) [100]. QTLs which explain less than 10% of the phenotypic variation would then require higher LD and/or higher panel size to achieve the same power [101]. Given LD decrease values obtained in this study and considering that the size of the maize genome is around 2.5 10^9^ bp [102], an average *r^2^* of 0.1 or 0.3 between adjacent markers would be expected when using 10450 (Table 2) or 75000 markers evenly distributed across the genome. The 50K Illumina array should be sufficient to obtain a global *r^2^_s_*, which better reflects the power of association genetics considering the population structure [34], of 0.1 but not 0.3. We would therefore require 100000 markers with higher density on chromosomes 1, 5 and 6 (Table 2). A slightly lower density would be required to work on specific genetic groups (dents and flints, for exemple, see [89]). However, given the LD variation between and along the chromosomes, and the fact that r*^2^_s_* does not account for the bias due to relatedness, a higher marker density would be preferable.

### Association genetics and colocalisation with QTLs and genes involved in flowering time

It is generally difficult to determine suitable significance threshold in GWAS studies. Approaches such as Bonferroni or FDR do not account for LD between markers and therefore are generally too stringent. Moreover, the high genetic variance estimates obtained with lmm (up to 34%) were difficult to interpret biologically and may have resulted from confusion between estimations of fixed effects and variance in random effects, *i.e.* with random effects being putatively deflated by LD between the marker tested as fixed effect and distant markers associated with the variation of the trait. On the other hand, genetic variances estimated with lm may be underestimated because of confounding effects between structure and marker effects, as confirmed by the high differentiation rate observed at these loci. The real genetic variance is probably intermediate. The colinearity of markers with genetic groups and families of related materials probably also explains why highest absolute effects corresponded to markers with extreme frequencies (Figure 3). Individual association signals should therefore be examined in the light of associations in independent experiments and/or further biological or functional information.

Here we report information on markers associated to flowering traits with *P*-value<10^−5^ (Table S8), while highlighting markers with *P*-value<10^−7^ (Table 3), which represents a break point in the logarithm of cumulative *P*-value curves. The multi-locus analysis of FFLW8 showed that the nine first markers added in the model were significant with (*P*-value<10^−3^) conditionally to the previous model and the last one to enter the model with (*P*-value<0.05) was the 29^th^. The augmentation in *P*-values relative to those observed in individual tests suggests that some colinearity exists between flowering time QTLs beyond that expected when considering relatedness and population structure only. This is possibly due to differential selection. However, the main QTLs detected in the single marker analysis remained significant in the multi-locus analysis, which confirmed their effect. The fact that AIC continued to decrease until 71 markers were included in the model confirms that numerous regions are involved. Interestingly, the multi-locus analysis also highlighted several cases where linked markers entered the model. This suggests that several SNP markers are necessary in these regions to tag allelic series with a gradient of effects (see [4], [32]) and that haplotype based models should be considered in further investigations. This was the case in particular for the ZCN8 region of chromosome 8 (see below), the centromeric and an additional region of chromosome 8, two regions of chromosome 2 and one of chromosome 5 (see Table S8).

The most significant association in our study was found 5000 bp from *ZCN8* for the closest marker to this gene in the 50K Illumina array. This position is 8 Mb from *Vgt1* and 1 Mb from a marker found to be associated in the NAM population [4]. The association was higher than that reported for Vgt1 *Mite* with the same panel [21]. A large family of 25 homologues to the Arabidopsis *FT* locus, *i.e. ZCN* genes, was recently described in maize, suggesting that maize like *Arabidopsis* contains FT-related proteins that act as a florigen [17]. The question remains as to which maize ZCN genes have a role similar to that of *Arabidopsis FT*. We confirmed above that *ZCN8*, the most similar homologue to *Arabidopsis FT*, had a major effect on flowering time variation. This is to our knowledge the clearest evidence reported so on the involvement of this gene in natural variation of flowering time in maize. Its expression and function were already examined by [14]. In teosinte, which requires short-day photoperiods to induce flowering, they showed that ZCN8 was highly upregulated in leaves under inductive photoperiods. A less prominent increase was detected in temperate maize. QTLs in this region of chromosome 8 have been highlighted in numerous studies and their meta analysis concluded on the presence of two meta QTLs (green and blue zones in [22], Figure 3). For the “blue zone”, Vgt1 has been cloned by a map based approach and confirmed in numerous association mapping panels, including the present panel, and therefore has an unambiguous position. For the “green zone”, our results positioned *Vgt2* on *ZCN8*, outside but close to the zone proposed by [22]. This suggests that meta QTL analyses are useful to describe the number of QTLs underlying the variation of flowering time, but caution is necessary with respect to the exact position estimation. Association studies such as the whole genome scan described here are of considerable interest for refining these positions.

We found additional associations and/or traces of selection in the vicinity of *ZCN4*, *ZCN7* for which expression analysis demonstrated involvement in developmental processes [17], ZCN15 which is homologue to rice *Hd3* according to [103] and ZCN21, thus suggesting that they could be involved in flowering time variation. We also confirmed, with an association study approach, that *Dfl1* or nearby polymorphism was involved in flowering time variation. *Dfl1* is known to regulate flowering time in the shoot apical region and mutants exhibit a less severe flowering time defect compared to *ID1* [18]. We found that many associations were close to regulation factors, binding sites, kinase proteins (Table 3), which is consistent with the fact that trait variation may be controlled by non-genic regions regulating gene expression [104]. The broader integration of biological information and annotation such as regulatory elements or gene pathways would be of great value in gaining further insight into the control of flowering time.

### Divergent patterns of diversity and traces of selection colocalise with flowering time QTLs

The progressive migration of maize from the tropics to temperate environments has shaped adaptive traits like flowering time and led to allelic differentiation among groups due to selection and demographic events [105]. The fact that the phenotype distribution matches the genetic structure makes it difficult to distinguish between neutral loci versus loci that control adaptive traits like flowering time. Association genetics leads to many false positives when structure is not included in the model, and differential allelic fixation at key causal polymorphisms sites reduces the capacity to detect associations when population structure is included as a covariate. We therefore used selection tests as a complement to association genetics to detect loci that presented significant levels of differentiation among groups. Standard selection test approaches consist of finding putative advantageous mutations that spread rapidly within the population, eliminating variation at linked sites. These loci are detected by comparing locus specific differentiation with a distribution of differentiation rates simulated under demographic hypotheses/scenarios such as infinite island or hierarchical models. An alternative proposed by Foll and Gaggiotti [47] is to directly estimate the probability that each locus is subject to selection using a Bayesian method. According to this model, we found 52 markers (11 regions) presenting significant traces of selection (log*_10_(PO)*>0.5) and 18 markers or 9 regions that were highly significant (*log_10_(PO)*>1). Most markers associated with flowering time (34/35) showed high differentiation among groups (Figure S17 in Text S1), as observed previously for the *Vgt1* locus [21]. In particular, both ZCN8 and the centromere of chromosome 8 presented signatures of diversifying selection with a tropical allele and a temperate allele. Considering all associations detected with a *P*-value below 10^−5^, we observed a general pattern of early allele propagation in temperate lines that we could describe as being a blue wave during maize expansion (Figure 3). The average proportion of early alleles accumulated in NF, EF, CBD and tropical genomes was 0.87, 0.83, 0.41, 0.48 and 0.39, respectively (Figure S22 in Text S1), congruent with the precocity of these groups (adjusted female flowering times in GDD were 762, 763, 943, 889 and 1181, respectively). The number of early alleles gathered in one genome was closely correlated to the precocity of the line (Figure S21 in Text S1), indicating that the loci were putatively numerous and the effects were small and mainly additive. The fact that early alleles were rare in the tropical materials suggests that they are generally not beneficial in most tropical conditions since they excessively shorten the plant cycle. Conversely, selection rapidly drove these early alleles to near fixation in early flowering flints. This is in line with the hypothesis of a northward expansion through northern USA and Europe for flints, accompanied by directional selection for early flowering that gradually led to the accumulation of an increasing number of early alleles. Corn Belt Dents, which are intermediate between flints and tropicals with respect to flowering date, presented higher levels of diversity at these QTLs. This is consistent with the development of this material from an hybridization between Southern Dent (derivated from tropicals) and Northern Flints (see [105] for a review). This higher level of polymorphism allows many different putative combinations of early-late alleles and adjustment of flowering date to local environmental conditions. This also explains why more associations could be detected within the dent group.

We also observed that some additional markers which showed a significant signature of diversifying selection were not associated with flowering time in our panel. Markers #3, #8, #10 and #25 are located in regions that have never been reported as associated to flowering time to our knowledge and illustrate the differentiation of other adaptive traits. Some other outlier markers were associated with flowering time in other studies, suggesting that they were false negatives in our study. The best false negative example was noted on chromosome 7 (#18), which was clearly associated with flowering time in a NAM design [4]. We also observed some markers on the centromere of chromosome 8 (50 and 95 Mb) that could be closer to causative loci than surrounding associated markers. This suggests that no single GWAS design or analysis method is sufficient to unravel the complex genetics underlying natural variation in complex traits like flowering time. The mixed model has the greatest power to find associations when alleles segregate within several genetic groups. When different alleles have high frequencies in different groups, the naive approach yields many false signals, while the mixed model approach misses them entirely due to colinearity between causal polymorphism and structure covariates. This highlights the utility of multiparental linkage based approaches like NAM designs to break up the observed structure in founder lines and reach more balanced allele frequencies, thus increasing the statistical power [4].

From an evolutionary standpoint, geographical distance along with flowering time divergence ultimately results in reproductive barriers between lines. Even if admixture is still possible between compatible lines, genomic regions involved in adaptive traits will tolerate only restricted gene flow, resulting in lower efficient recombination, lower diversity and higher LD compared to the rest of the genome. This pattern can be dramatic when it occurs in centromeric regions (see chromosome 8 in this study) that have mechanical barriers against crossover. Recent empirical and theoretical studies suggest that restricted recombination regions play an important role in the formation of new genetic groups and species. They could contain clusters of genes involved in reproduction isolation and centromeres may be privileged regions from this standpoint [106], [107], [108], [109].

### Conclusion

Our data generally show that major differences in flowering time among inbred maize lines are caused by a few genes with relatively marked effects (*Vgt1-Vgt2/ZCN8*, centromere of chromosome 8), and the cumulative effects of many loci with small to intermediate effects. In view of selection, this suggests that predictive methods offering flexibility in effect magnitude such as Bayes B [110], Bayes Cpi [111] or Lasso [112] should *a priori* be more adapted than RA-blups [113] or ridge regression RR-blups [110] methods which constrain the range of variation of effects to fit the same distribution. The complex network of genes that may be involved in signaling pathways that control levels of florigen expression, accompanied by a wide range of QTL effects and an open pollinated reproductive system, may have facilitated flowering time adaptation to local environments. Alleles that increase precocity were collectively found at higher frequencies in European and Northern Flints than in dents and tropicals and their number was correlated with precocity, consistent with the assumption that this phenotypic shift is selectively driven by many small effect loci. However, genes with larger effects remained detectable via association genetics. This shows that polygenic selection for flowering time does not necessarily lead to fixation, which allows flexibility in flowering time and secure rapid adaptation in case of environmental changes. This is of great interest for conservation management and more efficient breeding use of diversity available in maize germplasm repositories. It is now essential to strive to gain greater insight into genetic-genetic background (epistasis) and genetic-environment interactions and their phenotypic consequences in order to enhance breeding efficiency in the future under changing climatic conditions [114].

## Supporting Information

Text S1 **Supporting Text including Data S1, Figures S1–S10, Figures S13–S22, and Tables S1–S7.**
(DOC)

Figure S11 **Supporting Figure including one page per chromosome (10).**
(PDF)

Figure S12 **Supporting Figure including one page per chromosome (10).**
(PDF)

Table S8 **Supporting Table including information about markers.**
(XLS)
