# Supplementary material for: Adaptation of Maize to Temperate Climates: Mid-Density Genome-Wide Association Genetics and Diversity Patterns Reveal Key Genomic Regions, with a Major Contribution of the Vgt2 (ZCN8) Locus
Source: PLoS One. 2013 Aug 30;8(8):e71377. doi: 10.1371/journal.pone.0071377 (PMC3758321; doi:10.1371/journal.pone.0071377)
Supplement: Text S1 — Supporting Text including Data S1, Figures S1–S10, Figures S13–S22, and Tables S1–S7. (DOC) [file pone.0071377.s001.doc]

**Supporting Information Text S1**

**Adaptation of maize to temperate climates: mid-density genome-wide association genetics and diversity patterns reveal key genomic regions, with a major contribution of the *Vgt2* (*ZCN8*) locus**

Authors: Authors: Sophie Bouchet^15^, Bertrand Servin^2^, Pascal Bertin^1^, Delphine Madur^1^, Valérie Combes^1^, Fabrice Dumas^1^, Dominique Brunel^3^, Jacques Laborde^4^, Alain Charcosset^1^*, Stéphane Nicolas^1^

^1^UMR de Génétique Végétale, INRA – Université Paris-Sud – CNRS, 91190 Gif-sur-Yvette, France,

^2^UMR444, Laboratoire de Genetique Cellulaire, INRA, 31326 Castanet-Tolosan, France,

^3^UR1279, Etude du Polymorphisme des Génomes Végétaux, INRA, Commissariat à l’Energie Atomique (CEA) Institut de Génomique, Centre National de Génotypage, 91057 Evry, France,

^4^INRA Stn Expt Mais, 40590 St Martin De Hinx, France

*Corresponding author: Alain Charcosset, E-mail: alain.charcosset@moulon.inra.fr

**Contents**

**List of Supplementary Data**

**Data S1.** Lines DNA label check.

**List of Supplementary Figures**

**Figure S1.** Variation in the physical size of intervals between two consecutive SNPs of the 50K Illumina array along each chromosome.

**Figure S2.** Distribution of the physical size of all intervals between adjacent SNPs originating from the 50K Illumina array.

**Figure S3.** Distribution of the minimum allele frequency (MAF) within four genetic groups (NF, EF, Trop, CBD) and in the whole panel (Panel).

**Figure S4.** Distribution of frequencies of alleles that were rare in Tropicals (< 0.05) in the four other genetic groups (NF, EF, SS, CBD).

**Figure S5**. Correlations between identity by state (IBS) similarities estimated using Panzea and non-Panzea SNPs.

**Figure S6.** Boxplot of two similarity indexes (IBS and IBD) of inbred lines of the whole panel.

**Figure S7.** Correlations between different similarity indexes (IBS and IBD) computed for different sets of markers.

**Figure S8.** Comparison of inbred line assignments to groups inferred from different marker sets for a number of groups ranging from 2 to 10 (Q2 to Q10).

**Figure S9.** Boxplot of assignment to main genetic groups depending on the marker set (55 SSRs, 94 SSRs, 29911 Panzea SNPs).

**Figure S10.** Distribution of *F_ST_*  (*F_ST_*) for 43224 SNPs.

.

**Figure S11**. Supporting Figure including one page per chromosome (10). (PDF file)

**Figure S12.** Supporting Figure including one page per chromosome (10). (PDF file)

**Figure S13.** Distribution of *F_ST_* and *F_STB_* according to selection tests obtained with BayeScan.

**Figure S14.** Relation between MAF and Nei *F_ST_* (*F_ST_*) or Bayesian *F_ST_* (*F_STB_*)

**Figure S15**. Differences between *r^2^* and *r^2^_s_* (*r^2^*- *r^2^_s_*) as a function of the physical distance (bp) between loci.

**Figure S16.** Proportion of LD corrections (*r^2^-r^2^_s_*) with absolute values above 0.01 for increasing distance classes.

**Figure S17.** Boxplot of different diversity indexes for SNPs within or outside of centromeres, and for those associated with FFLW8 (*P*-value < 10^-5^).

**Figure S18**. Observed (Y axis) vs. expected (X axis) cumulative *P*-value, expressed as –*log10*(*P*-value).

**Figure S19.** Distribution of absolute effects for FFLW8 (GDD) association tests (*P*-value < 10^-5^) with the mixed model Q_55SSRs_+K_IBS(94SSRs)_.

**Figure S20.** Distribution of the proportion of genetic variances explained by SNP associated with FFLW8 (*P*-value < 10^-5^) with the Q_55SSRs_+K_IBS(94SSRs)_.model.

**Figure S21.** Relationship between female flowering time (FFLW8) of inbred lines and the proportion of early alleles accumulated in their genome.

**Figure S22.** Boxplot of the frequency of early alleles within each group.

**List of Supplementary Tables**

**Table S1.** Distribution of SNP markers along the 10 maize chromosomes.

**Table S2.** Pairwise differentiation between five main maize genetic groups.

**Table S3.** LD decrease as obtained with two different non-linear models and average LD by intervals of 20 kb among markers.

**Table S4.** Physical position of centromeres.

**Table S5.** Adjusted means computed for three flowering traits.

**Table S6.** Number of markers associated with flowering time according to different *P*-value thresholds.

**Table S7.** Number of regions associated with flowering time according to different *P*-value thresholds.

**Table S8.** Supporting Table including information about markers (EXCEL file).

**Data S1. Line DNA label check.**

DNA sample check with 8 SSR revealed illegitimate or strong divergence between successive seed lots (line F748 illumina code WG0065680-DNAH10, C103_usa WG0065680-DNAB04, F544 WG0065680-DNAH07, KUI43 WG0128141-DNAC10, CO109_inra WG0008561-DNAA01). Phenotypic and genotypic correlation check revealed illegitimate seed lots (A1P_inra_illeg WG0065680-DNAE01, M162W_usa WG0128141-DNAH10, W64A_inra WG0128140-DNAA07).

F874_inra was not genotyped with 55 SSRs.

**Table S1.** Distribution of SNP markers along the 10 maize chromosomes.

| Chromosome | Length (Mb) | # Markers | Marker interval (kb) |
| --- | --- | --- | --- |
| 1 | 301.24 | 6892 | 43.71 |
| 2 | 236.97 | 4963 | 47.75 |
| 3 | 232.09 | 4938 | 47.00 |
| 4 | 241.24 | 4784 | 50.43 |
| 5 | 217.71 | 4711 | 46.21 |
| 6 | 169.10 | 3456 | 48.93 |
| 7 | 176.15 | 3562 | 49.45 |
| 8 | 175.73 | 3719 | 47.25 |
| 9 | 156.59 | 3129 | 50.04 |
| 10 | 150.18 | 3070 | 48.92 |
| Whole genome | 2057.01 | 43224 | 47.59 |


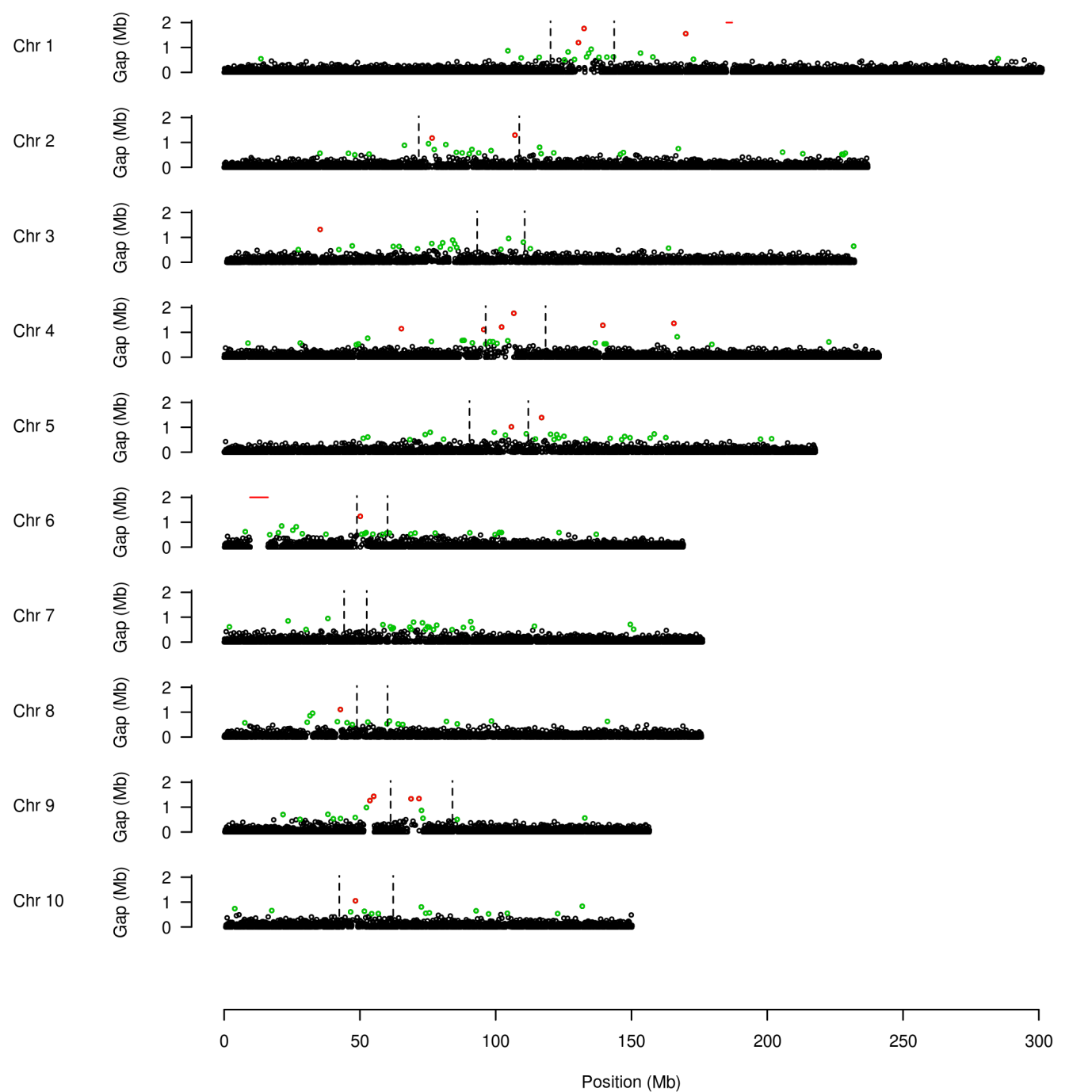


**Figure S1. Variation in the physical size of intervals between two consecutive SNPs of the 50K Illumina array along each chromosome.** Red, green, and black dots indicate that the interval sizes were between 1 Mb and 2 Mb, between 500 kb and 1 Mb or below 500 kb, respectively. Lines in red highlight intervals around 2 Mb starting at position 184908147 (bp) on chromosome 1 and 6.5 Mb at position 9501960 (bp) on chromosome 6. Dashed lines indicate centromere positions of flanking markers obtained from MaizeGDB and [66].

**
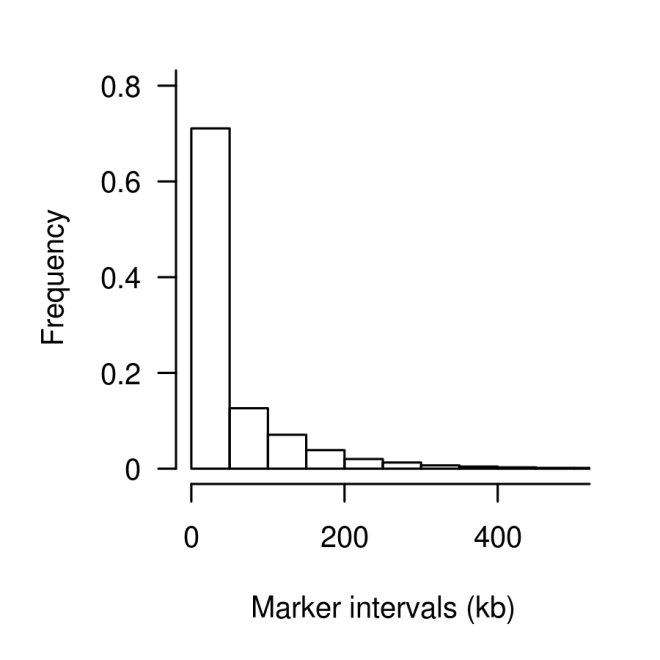
**

**Figure S2. Distribution of the physical size of all intervals between adjacent SNPs originating from the 50K Illumina array.**

**
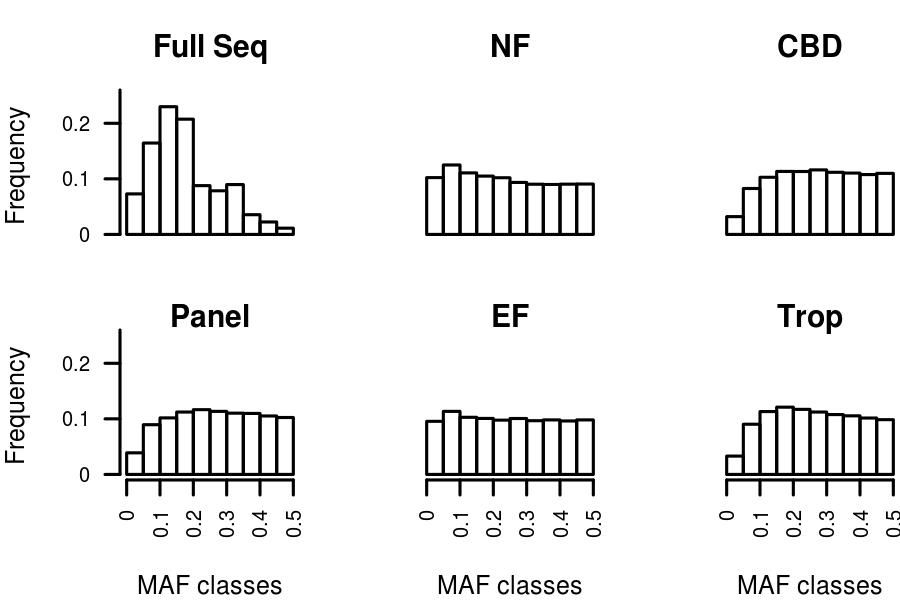
**

**Figure S3. Distribution of minimum allele frequency (MAF) within four genetic groups (NF, EF, Trop, CBD) and in the whole panel (Panel).** MAF were compared for the 29911 Panzea SNPs genotyped on the 50K Illumina array and for 535 SNP loci discovered within three sequenced regions (Full Seq) for the whole panel**,** next to *Vgt1* on chromosome 8 [21], *ZmCCT* on chromosome 10 [32] and *Tb1*-*Dwarf 8* on chromosome 1 [30]. Allele frequencies were estimated within four genetic groups (NF: Northern Flint, EF: European Flint, CBD: Corn Belt Dent, Trop: Tropical) by weighting allele sharing by assignment of each line to each of the five main genetic groups (see P_k(l)q_ calculation in Material and Methods for more details). The distribution of MAF for the Stiff Stalk group was removed because of the limited number of individuals within this group.

**
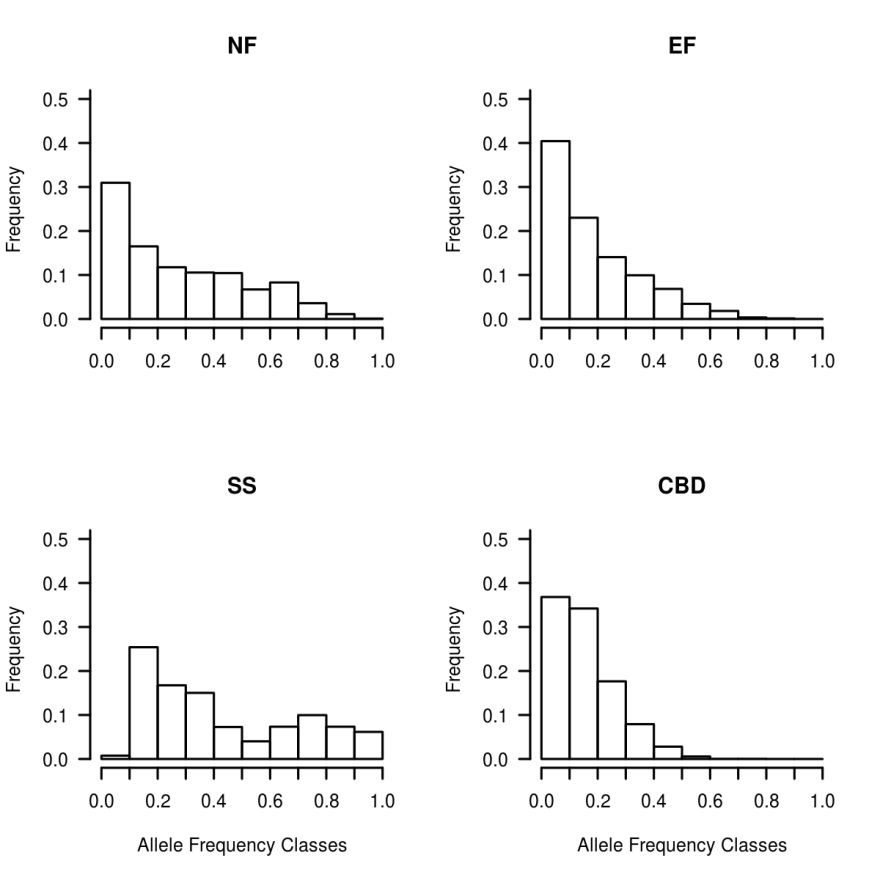
**

**Figure S4. Distribution of frequencies of alleles that were rare in Tropicals (< 0.05) in the four other genetic groups (NF, EF, SS, CBD).**

**
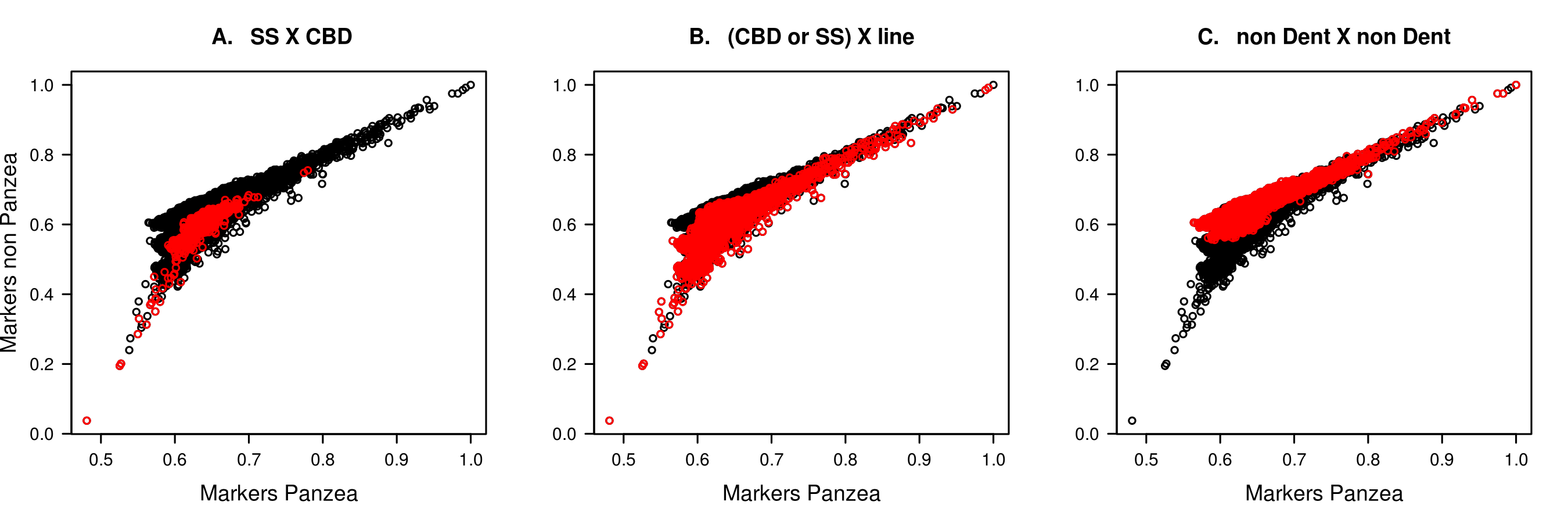
**

**Figure S5. Correlations between identity by state (**IBS**) similarities estimated using Panzea and non-Panzea SNPs.** Panzea markers were designed from 27 diverse founder lines, called the Panzea diversity panel [36]. Non-Panzea SNPs correspond to markers that were defined for mapping purpose and were polymorphic between B73 and Mo17 [31]. Red dots indicate similarities between (A) one SS line and one CBD line, (B) one dent line (SS or CBD) and any other line, (C) non-dent lines (neither SS nor CBD), respectively.


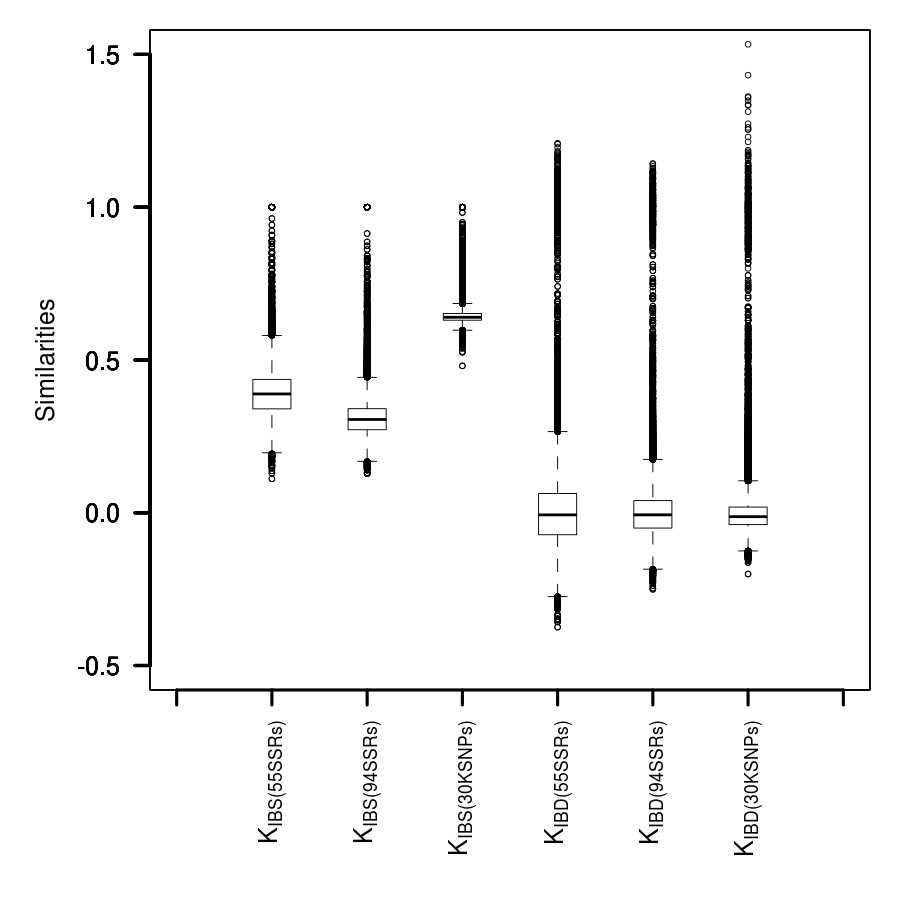


**Figure S6. Boxplot of two similarity indexes (IBS and IBD) among inbred lines of the whole panel.** Similarities were estimated from a set of 55 SSR markers (K_IBS(55SSRs)_ and K_IBD(55SSRs)_), a set of 94 SSR markers (K_IBS(94SSRs)_ and K_IBD(94SSRs)_) and a set of 29911 Panzea markers (K_IBS(30KSNPs)_ and K_IBD(30KSNPs)_) (see Material et Methods for more details).


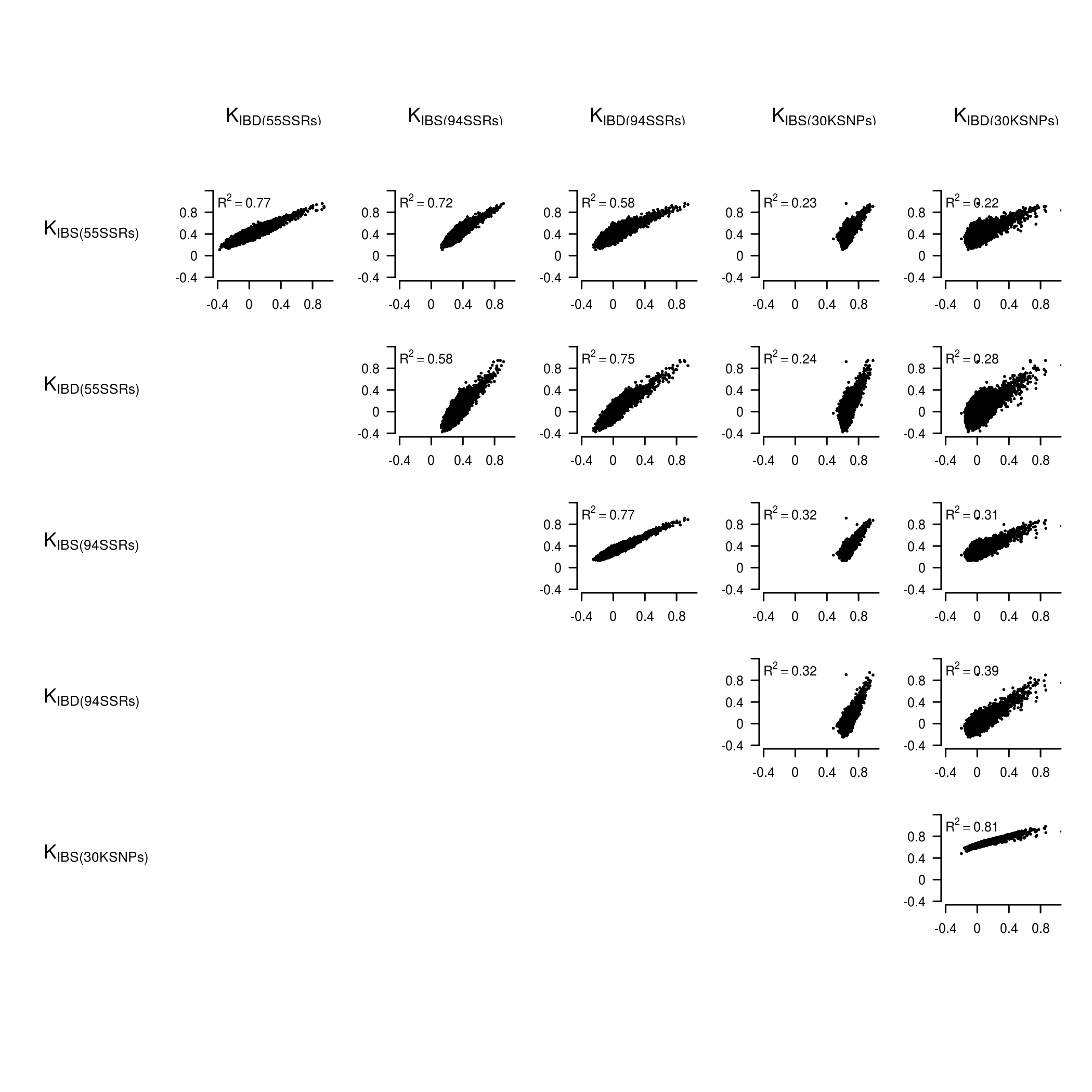


**Figure S7. Correlations between different similarity indexes (IBS and IBD) computed with different sets of markers.** Sets of 55 SSR markers (K_IBS(55SSRs)_ and K_IBD(55SSRs)_), 94 SSR markers (K_IBS(94SSRs)_ and K_IBD(94SSRs)_) and 29911 Panzea markers (K_IBS(30KSNPs)_ and K_IBD(30KSNPs)_ ) were represented. Each dot indicates one pair of inbred lines. Coefficients of determination (*R²*) were indicated on each graph.

**Table S2.** Pairwise differentiation between the five main maize genetic groups.

|  | NF | EF | SS | CBD | Trop |
| --- | --- | --- | --- | --- | --- |
| NF | - | 0.09 | 0.34 | 0.14 | 0.21 |
| EF | 0.09 | - | 0.30 | 0.11 | 0.13 |
| SS | 0.34 | 0.30 | - | 0.18 | 0.24 |
| CBD | 0.14 | 0.11 | 0.18 | - | 0.07 |
| Trop | 0.20 | 0.12 | 0.23 | 0.07 | - |

Upper and lower parts of the matrix correspond to upper and lower limits of confidence interval (97.5% and 2.5%) for pairwise *F_ST_* among the 5 main genetic groups, considering only those lines with assignment to one genetic group > 0.8, using 29911 Panzea markers and obtained with 100 bootstraps over loci (r-Hierfstat boot.ppfst()) [46].


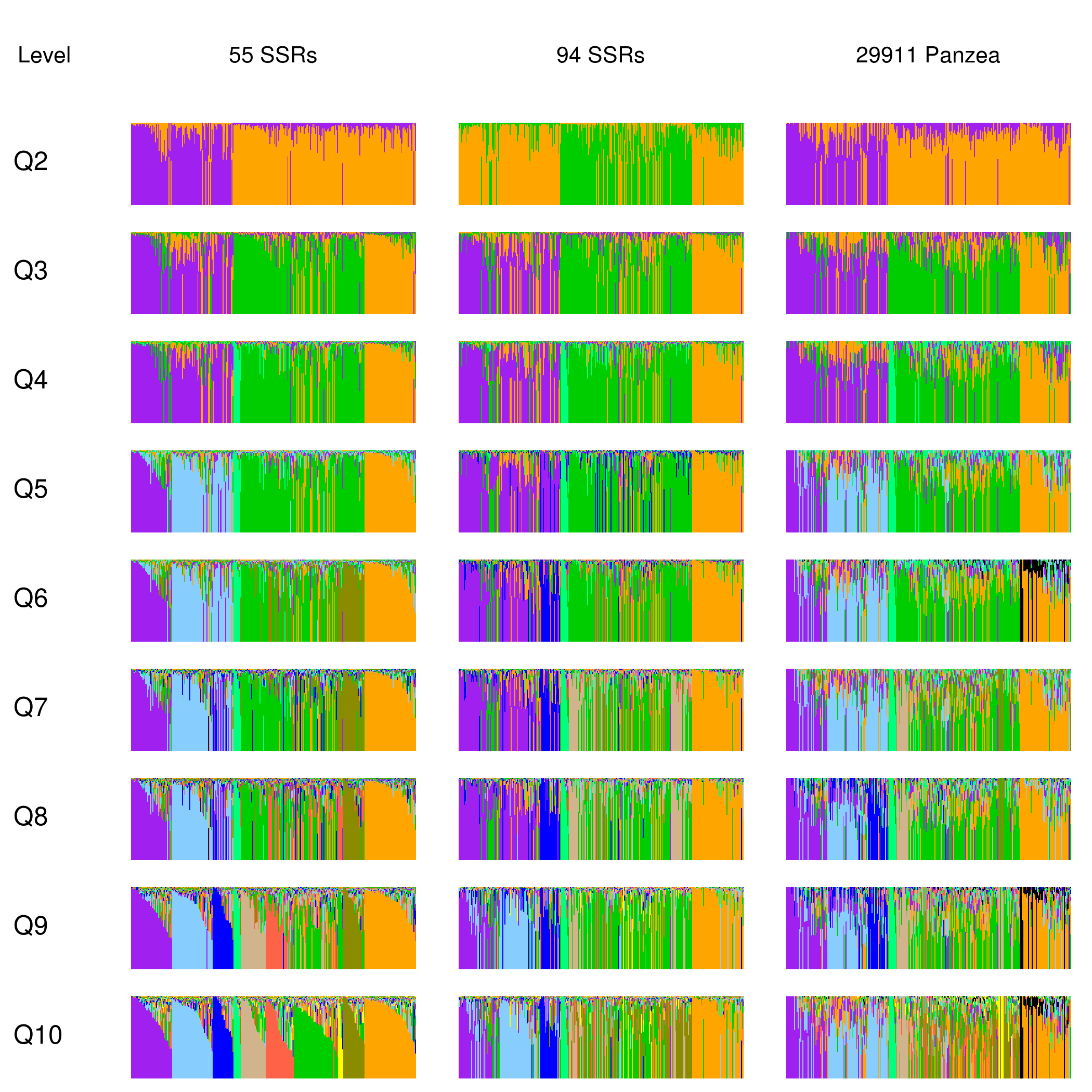


**Figure S8. Comparison of inbred line assignments to groups inferred from different marker sets for a number of groups ranging from 2 to 10 (Q2 to Q10).** Different sets of markers include 55 SSRs, 94 SSRs and 29911 Panzea SNPs, respectively. Lines are represented on x-axes for each marker system. Assignment proportions to each group at each level are represented on y-axes with different colors. Reference for ranking lines is Q10 for 55 SSRs. For 55 or 94 SSRs, assignments were obtained using STRUCTURE software [40,41]. For 29911 Panzea markers, assignments were obtained using ADMIXTURE software [42].


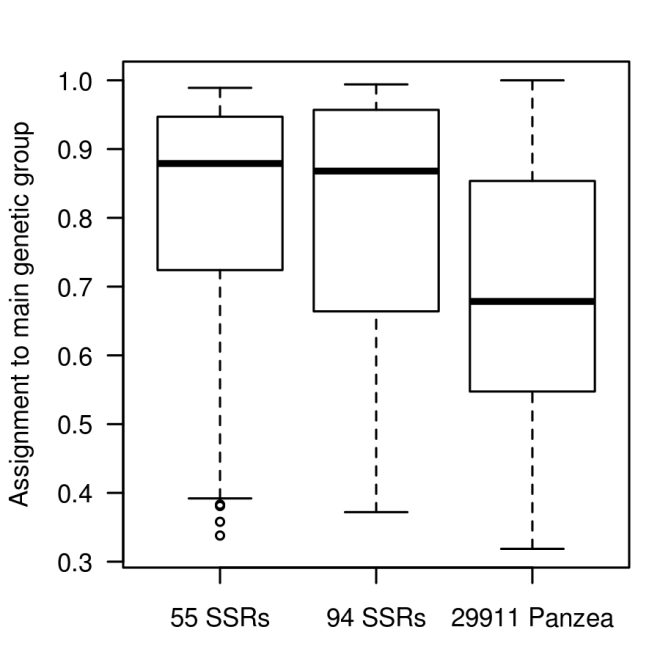


**Figure S9.** **Boxplot of assignments to main genetic groups depending on the marker set (55 SSRs, 94 SSRs, 29911 Panzea SNPs).** Assignments were obtained according to Figure S9 at level Q5.


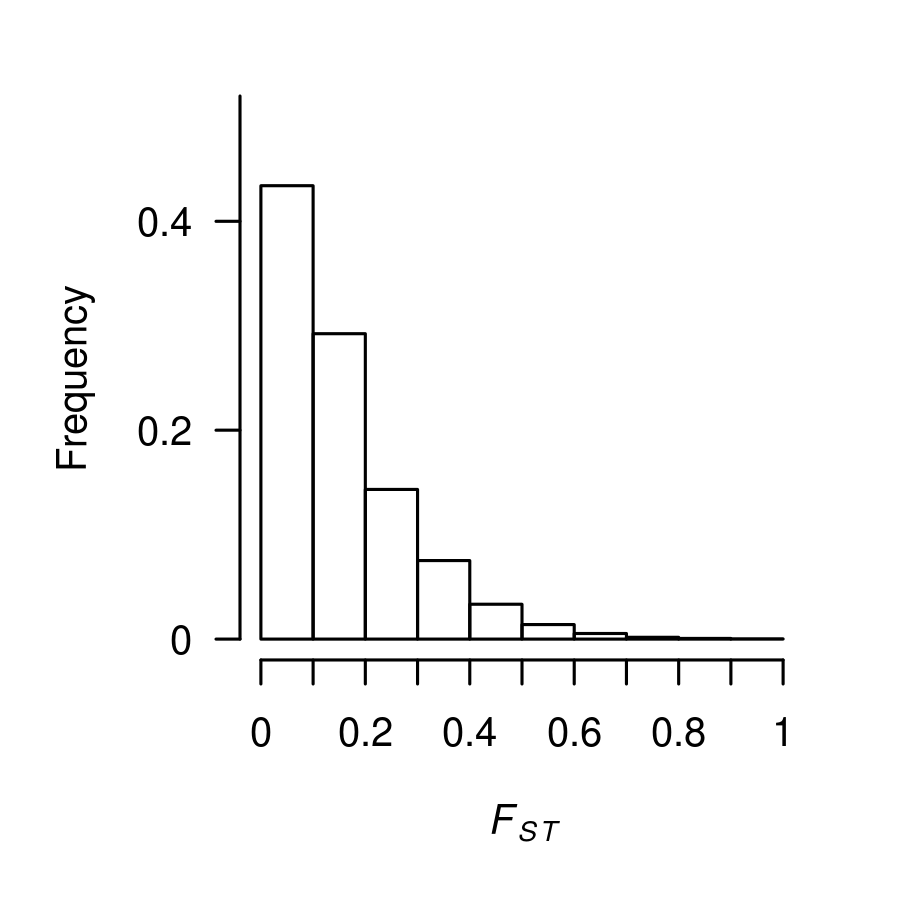


**Figure S10. Distribution of *F_ST_* (*F_ST_*) for 43224 SNPs.** *F_ST_* were estimated using r-Hierfstat [46] considering a qualitative assignment for non-admixed lines with assignment to one genetic group higher than 0.8.


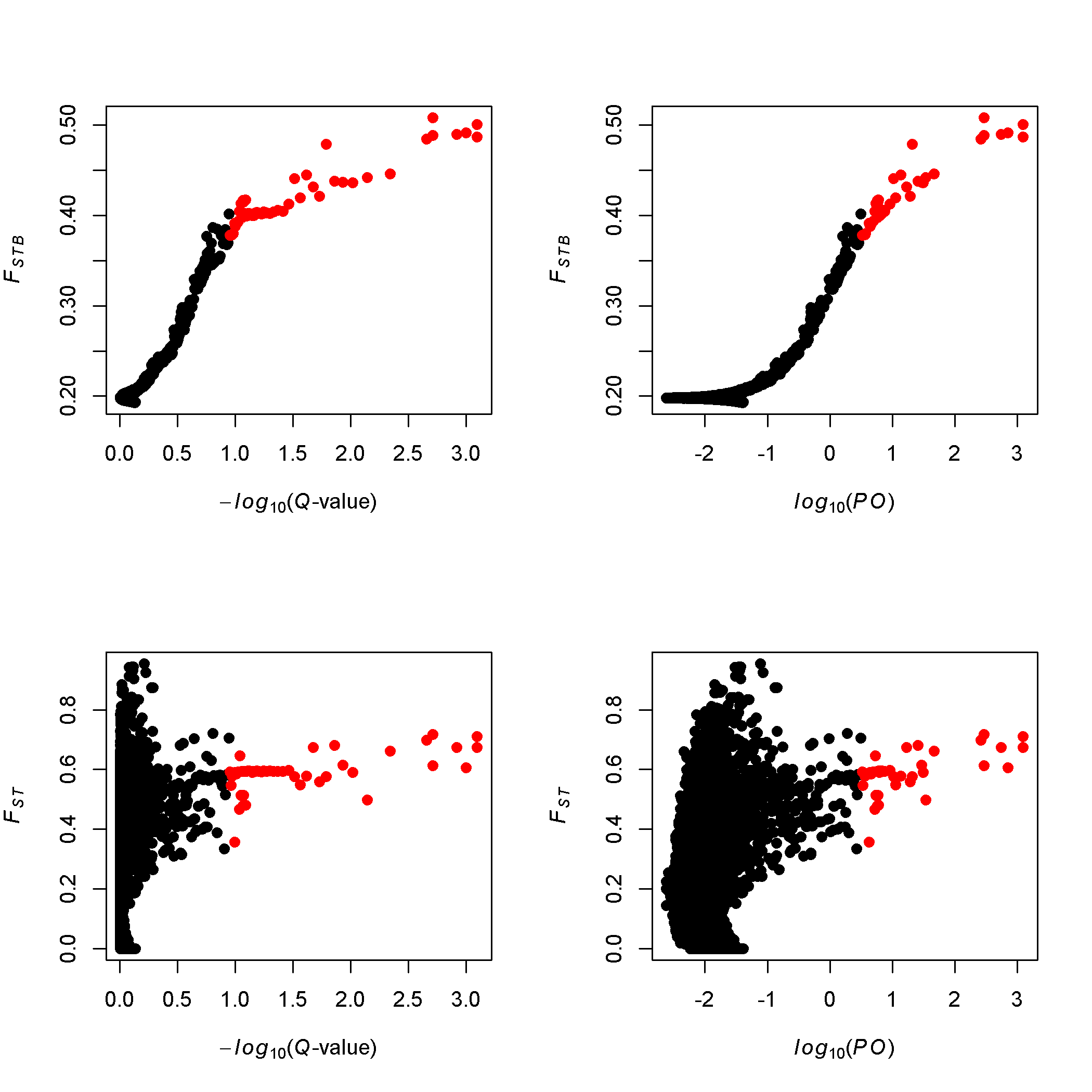


**Figure S13. Distribution of** *F_ST_* **and *F_STB_* according to selection tests obtained with Bayescan.** *Log_10_* of posterior odds (*PO*) is the ratio of posterior probabilities of the models with and without selection. According to Jeffrey’s scale, selection is significant when *log_10_(PO)* > 0.5. The *Q*-value of given locus is the minimum FDR at which this locus may become significant. Red dots correspond to markers with *log_10_(PO)* > 0.5. It corresponds approximately to *Q*-value > 0.1. *F_STB_* corresponds to differentiation calculated with BayeScan [47] and *F_ST_* corresponds to differentiation calculated with r-Hierfstat [48] considering non-admixed lines with assignments to the main genetic group above 0.8.


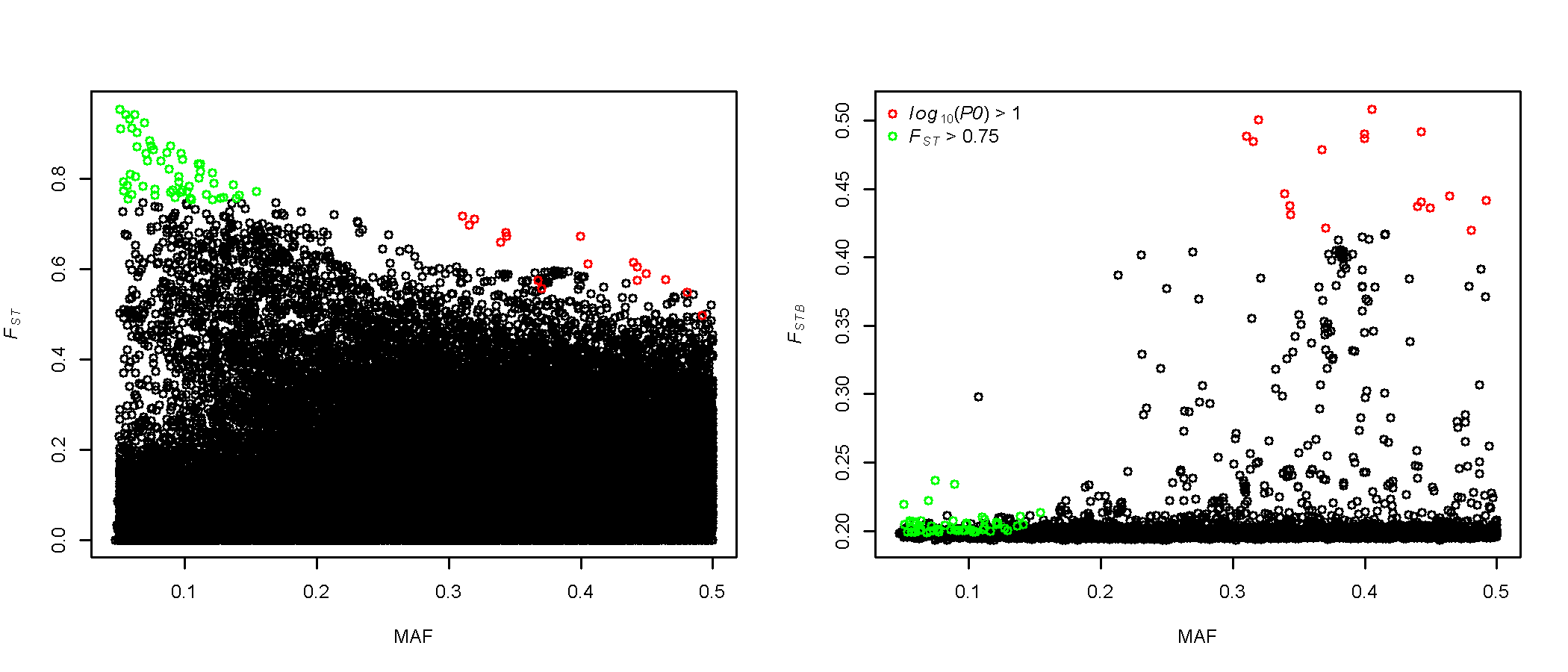


**Figure S14 Relation between MAF and Nei *F_ST_* (*F_ST_*) or Bayesian *F_ST_* (*F_STB_*) based on 43224 SNPs. *F_ST_*** were calculated using r-Hierfstat [46], ***F_STB_*** were estimated using a Bayesian algorithm implemented in BayeScan [47], both using only lines with assignment > 0.8 according to STRUCTURE [40,41] results based on 55 SSRs and five genetic groups.

**Table S3.** LD decrease as obtained with two different non-linear models and LD average by intervals of 20 kb among markers.

|  | Hill and Weir (1988) ^a^ | | Sved (1971) ^b^ | | Average LD by intervals of 20 kb ^c^ | |
| --- | --- | --- | --- | --- | --- | --- |
| Chromosome | *r^2^* extent (kb) ^e^ | *r^2^_s_* extent (kb) ^f^ | *r^2^* extent (kb) ^e^ | *r^2^_s_* extent (kb) ^f^ | *r^2^* extent (kb) ^e^ | *r^2^_s_* extent (kb) ^f^ |
| 1 | 111.11 (2711) | 78.61 (3832) | 5.52 (54 562) | 5.11 (58 905) | 80 (3765) | 100 (3012) |
| 2 | 230.50 (1028) | 183.25 (1293) | 5.69 (41 633) | 5.32 (44 536) | 160 (1481) | 160 (1481) |
| 3 | 191.37 (1213) | 146.75 (1582) | 7.55 (30 729) | 6.87 (33 764) | 140 (1658) | 160 (1451) |
| 4 | 286.24 (843) | 233.92 (1031) | 7.65 (31 531) | 6.86 (35 177) | 260 (928) | 260 (928) |
| 5 | 144.89 (1503) | 112.97 (1927) | 7.73 (28 164) | 7.29 (29 852) | 140 (1555) | 140 (1555) |
| 6 | 156.89 (1078) | 121.36 (1393) | 6.05 (27 932) | 5.59 (30 246) | 100 (1691) | 100 (1691) |
| 7 | 199.92 (881) | 154.89 (1137) | 5.21 (33 829) | 4.80 (36 705) | 160 (1101) | 160 (1101) |
| 8 | 303.90 (578) | 211.58 (831) | 8.79 (20 002) | 7.50 (23 422) | 180 (976) | 180 (976) |
| 9 | 228.62 (685) | 176.52 (887) | 5.45 (28 722) | 4.99 (31 356) | 160 (979) | 180 (870) |
| 10 | 289.97 (518) | 237.90 (631) | 6.36 (23 602) | 5.86 (25 649) | 200 (751) | 200 (751) |
| Whole genome | 197.75 (10 402) | 152.37 (13 500) | 6.40 (321 458) | 5.90 (348 645) | 158 (13019) | 164 (12543) |

Linkage disequilibrium (LD) extent was estimated for *r^2^*=0.1 with non-linear regression using r-Nls and ^a^ Hill and Weir [53], ^b^ Sved [52] formula, ^c^ average LD by intervals of 20kb. The number reported into brackets indicates the number of equidistant markers that would be needed to reach an average *r^2^* of 0.1 between adjacent markers. LD was calculated as ^e^ *r^2^* in Plink [50] or ^f^ *r^2^_s_* in r-LDcorSV [34].

**Table S4.** Physical position of centromeres.

| Chr | Start (bp) | Stop (bp) |
| --- | --- | --- |
| 1 | 120194443 | 143631570 |
| 2 | 71679833 | 108681500 |
| 3 | 93184118 | 110670137 |
| 4 | 96312688 | 118370806 |
| 5 | 90346209 | 111997810 |
| 6 | 48891592 | 60173353 |
| 7 | 44179516 | 52511881 |
| 8 | 48891592 | 60173353 |
| 9 | 61300955 | 84072027 |
| 10 | 42409158 | 62250776 |

Markers flanking centromeric regions were obtained from Maize GDB (maizegdb.org) and [66]. Blast of primers were done on B73 RefGen_v2 sequence.


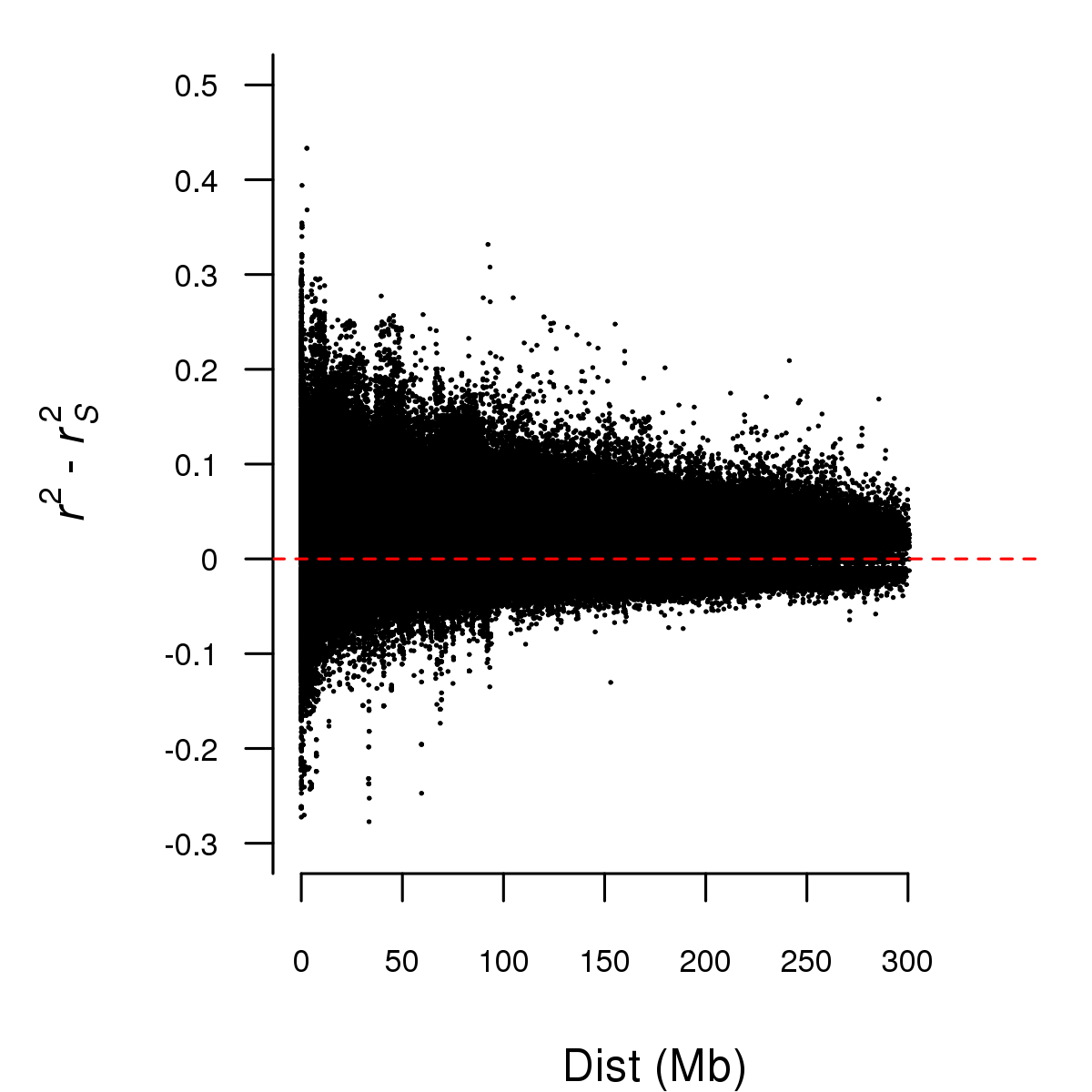


**Figure S15. Differences between *r^2^* and *r^2^_s_* (*r^2^*- *r^2^_s_*) as a function of the physical distance (bp) between loci.** *r^2^* was calculated with Plink [50]. *r^2^s* was calculated with r-LDcorSV [34]. Values ranged from -0.277 to 0.447. 40% of values were negative and 60% positive. Most values (85%) ranged from -0.01 and 0.01. Only 13% of the values were above 0.01 and 2% below -0.01. Less than 0.1% had an absolute value above 0.1. Considering only pairwise values for markers distant by less than 1 Mb, correction was greater. In that case, only 65% of values ranged from -0.01 to 0.01, 25% were above 0.01and 10% of values were below -0.01. Still only 1% of values were above 0.1 and 0.15% below -0.1.


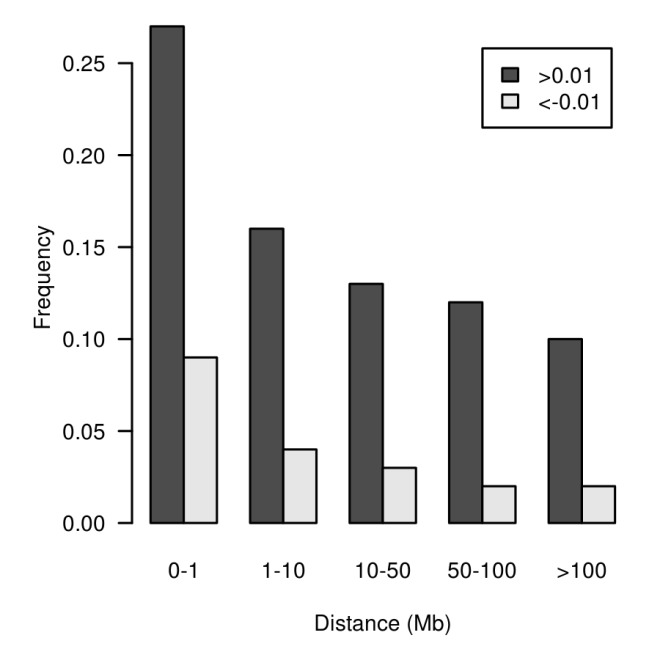


**Figure S16. Proportion of LD corrections (*r^2^- r^2^_s_*) with an absolute value above 0.01 for increasing distance classes.**


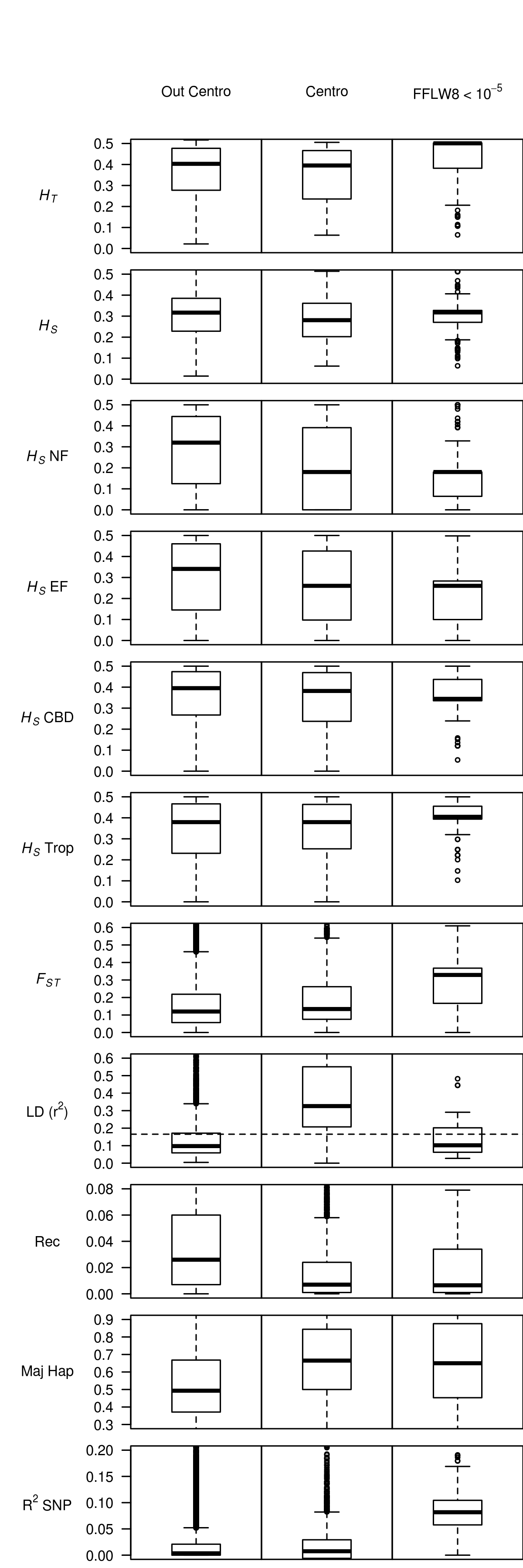


**Figure S17. Boxplot of different diversity indexes for SNPs within or outside of centromeres, and for those associated with FFLW8 (*P*-value<10^-5^).** Dashed lines correspond to the average along the genome. **Out centro** corresponds to regions outside centromeres according to maizeGDB and [66], **Centro** to centromeric regions, **FFLW8<10-5** to markers associated with *P*-value<10-5 with model Q_55SSRs_+K_IBS(94SSRs)_, ***H_T_*** to overall diversity for 242 non-admixed lines, ***H_S_* X** to diversity within genetic group X, **NF, EF, SS, CBD, Trop** to Northern Flint, European Flint, Stiff Stalk, Corn Belt Dent and Tropical, respectively, ***F_ST_*** to differentiation index. *H_T_*, *H_S_* and *F_ST_* were calculated with 242 non-admixed lines (assignment to one group > 0.8) in r-Hierfstat [46]. **LD** corresponds to linkage disequilibrium (*r²*) calculated with Plink [50] within a window of 1 Mb around associated markers. **Rec** corresponds to the probability of haplotype switch from one SNP to another calculated with fastPHASE [55] using 20 haplotype clusters. It is linked to the ancestral recombination rate. **MajHap** corresponds to the number of lines assigned the main haplotype cluster among 20.

**Table S5.** Adjusted means computed for three flowering traits.

| Group | FFLW8 | FFLW | MFLW8 | MFLW | ASI8 | ASI |
| --- | --- | --- | --- | --- | --- | --- |
| NF | 762.14 (±41) | 200.96 (±3.3) | 721.69 (±38) | 197.63 (±3.0) | 40.01 (±8.7) | 3.23 (±0.75) |
| EF | 762.85 (±35) | 201.06 (±2.8) | 734.21 (±33) | 198.73 (±2.6) | 27.64 (±7.5) | 2.21 (±0.65) |
| SS | 942.54 (±73) | 216.22 (±5.9) | 910.49 (±68) | 213.67 (±5.4) | 31.56 (±15.5) | 2.52 (±1.33) |
| CBD | 888.84 (±23) | 211.56 (±1.9) | 861.21 (±22) | 209.36 (±1.7) | 27.17 (±4.9) | 2.17 (±0.42) |
| Trop | 1180.85 (±33) | 235.14 (±2.6) | 1140.45 (±30) | 231.25 (±2.4) | 55.49 (±6.9) | 4.66 (±0.60) |

FFLW8 stands for female flowering date, MFLW8 for male flowering and ASI8 for anthesis to silking interval in GDD (following [56] with parameter values Tb = 8° and To = 30°C). FFLW, MFLW and ASI are expressed in days.


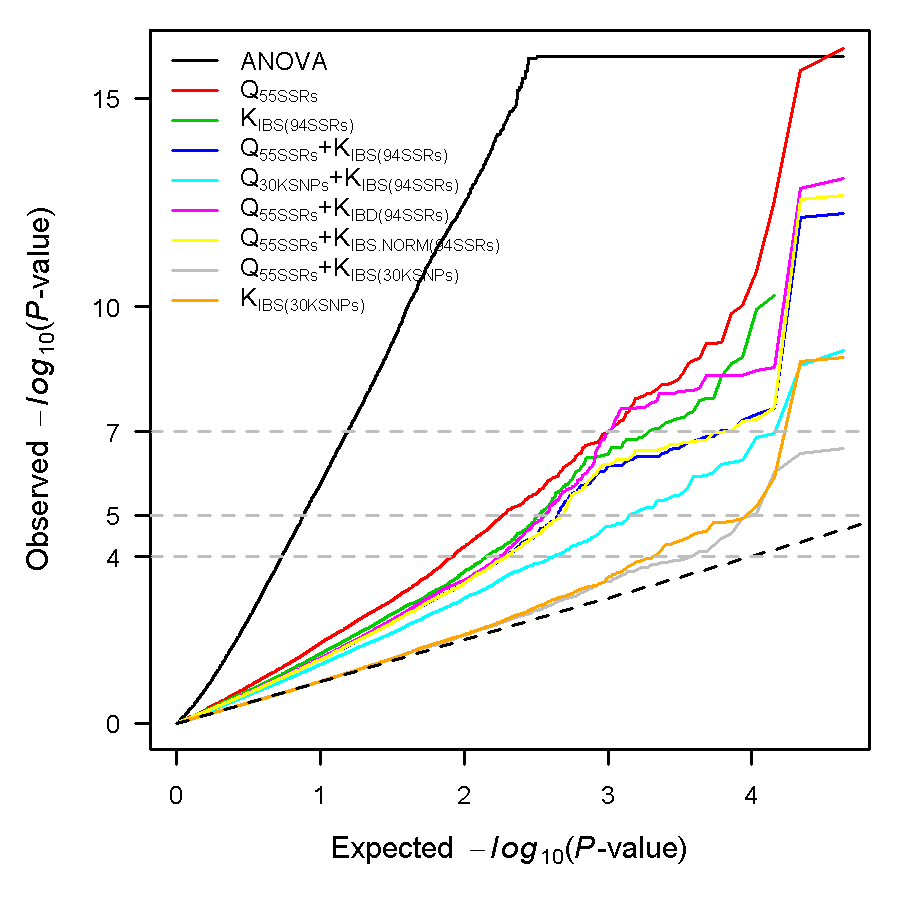


**Figure S18. Observed (y axis) vs. expected (x axis) cumulative *P*-value, expressed as –*log_10_*(*P*-value).** Associations were computed for 43224 SNPs. The dashed line corresponds to a uniform distribution of *P*-values. Q_55SSRs_ is a structure matrix computed with STRUCTURE [40,41] using 55 SSRs [30]. Q_30KSNPs_ is a structure matrix computed with ADMIXTURE [42] using 29911 Panzea SNPs. K_IBS(94SSRs)_ is a kinship matrix computed with the IBS index using 94 SSRs. K_IBD(94SSRs)_ is a kinship matrix computed with the IBD index [38] using 94 SSRs. K_IBS.NORM(94SSRs)_ is a kinship matrix computed with the IBS index using 94 SSRs and normalized according to [59]. K_IBS(30KSNPs)_ is a kinship matrix computed with the IBS index using 29911 Panzea SNPs.

**Table S6.** Number of markers associated with flowering time according to different *P*-value thresholds.

| # markers | FFLW8 | MFLW8 | ASI8 | FFLW8-MFLW8 |
| --- | --- | --- | --- | --- |
| *P*-value < 10^-5^ | 95 | 108 | 7 | 77 |
| *P*-value < 10^-6^ | 50 | 63 | 0 | 44 |
| *P*-value < 10^-7^ | 7 | 36 | 0 | 5 |

FFLW8 stands for female flowering date, MFLW8 for male flowering and ASI8 for anthesis to silking interval in GDD (following [56] with parameter values Tb = 8° and To = 30°C). FFLW, MFLW and ASI are expressed in days. FFLW8-MFLW8 indicates number of markers associated with both traits.

**Table S7.** Number of regions associated with flowering time according to different *P*-value thresholds.

| #Regions | FFLW8 | MFLW8 | ASI8 | FFLW8-MFLW8 |
| --- | --- | --- | --- | --- |
| *P*-value < 10^-5^ | 32 | 43 | 7 | 26 |
| *P*-value < 10^-6^ | 14 | 17 | 0 | 9 |
| *P*-value < 10^-7^ | 5 | 10 | 0 | 3 |

FFLW8 stands for female flowering date, MFLW8 for male flowering and ASI8 for anthesis to silking interval in GDD (following [56] with parameter values Tb = 8° and To = 30°C). FFLW, MFLW and ASI are expressed in days. FFLW8-MFLW8 indicates the number of regions associated with both traits.


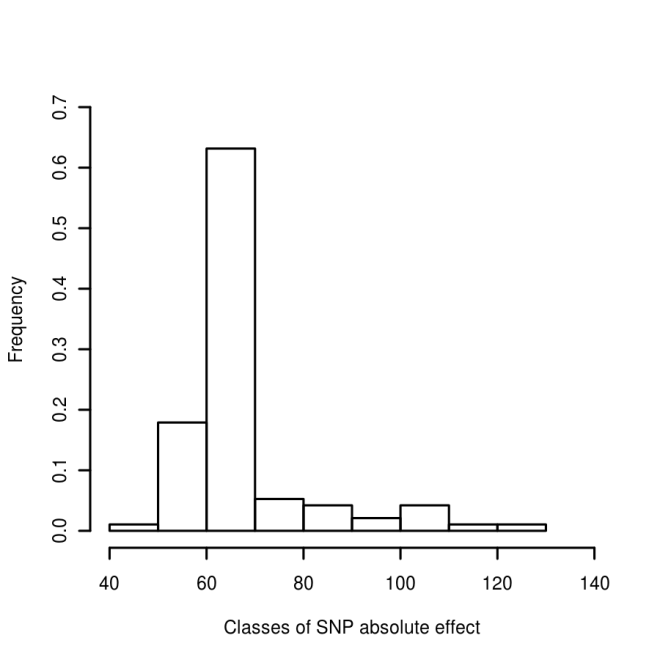


**Figure S19. Distribution of absolute effects for FFLW8 (GDD) association tests (*P*-value < 10^-5^) with the mixed model Q_55SSRs_+K_IBS(94SSRs)_.** Q is a structure matrix computed with STRUCTURE [40,41] using 55 SSRs [30]. K is a kinship matrix computed with IBS index using 94 SSRs.


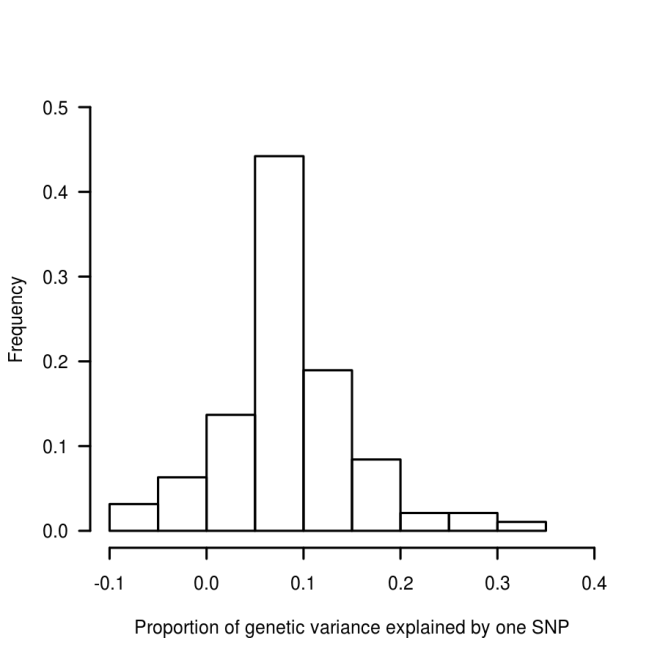


**Figure S20. Distribution of the proportion of genetic variances explained by SNP associated with FFLW8 (*P*-value<10^-5^) with the** Q_55SSRs_+K_IBS(94SSRs)_ **model.** Q_55SSRs_ is a structure matrix computed with STRUCTURE [40,41] using 55 SSRs [30]. K_IBS(94SSRs)_ is a kinship matrix computed with the IBS index using 94 SSRs. The proportion of genetic variance explained by significant SNPs was computed based on the relative reduction in polygenic variance when SNPs were added to the linear mixed model [65].

**
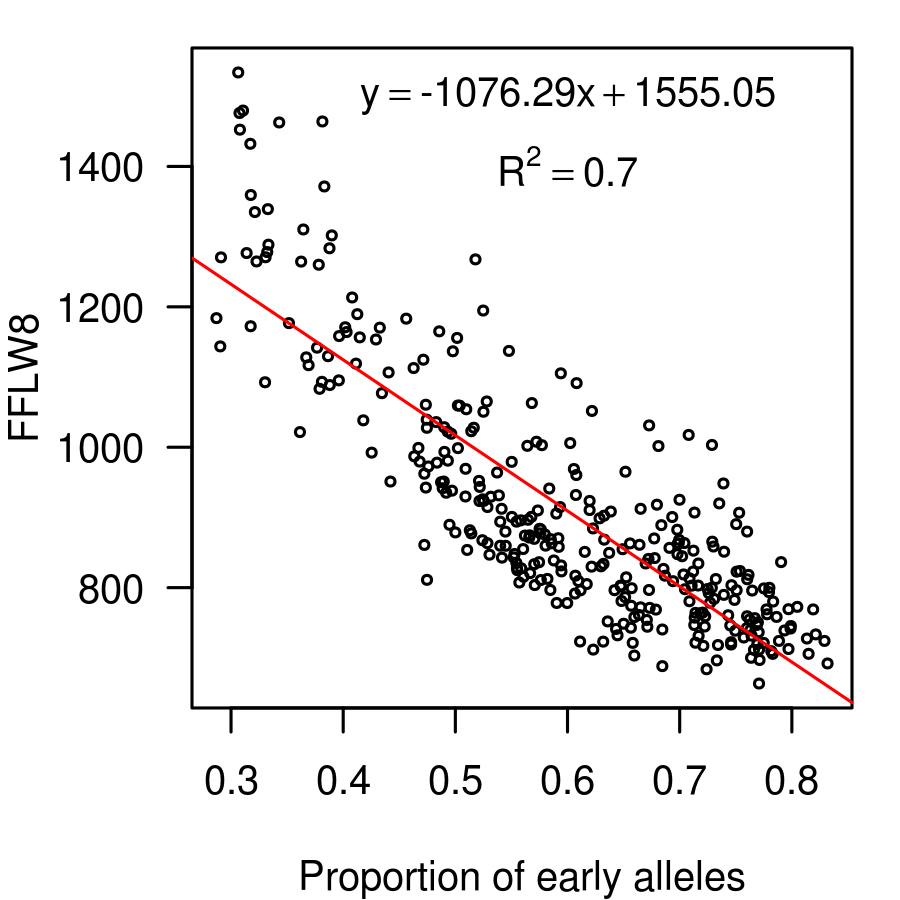
**

**Figure S21. Relationship between female flowering time (FFLW8) of inbred lines and the proportion of early alleles accumulated in their genome.** Only markers associated with flowering time with *P*-value <10^-5^ according to model Q_55SSRs_+K_IBS(94SSRs)_ were considered. Correlation coefficient between FFLW8 and proportion of early alleles in one genome is -0.84.

**
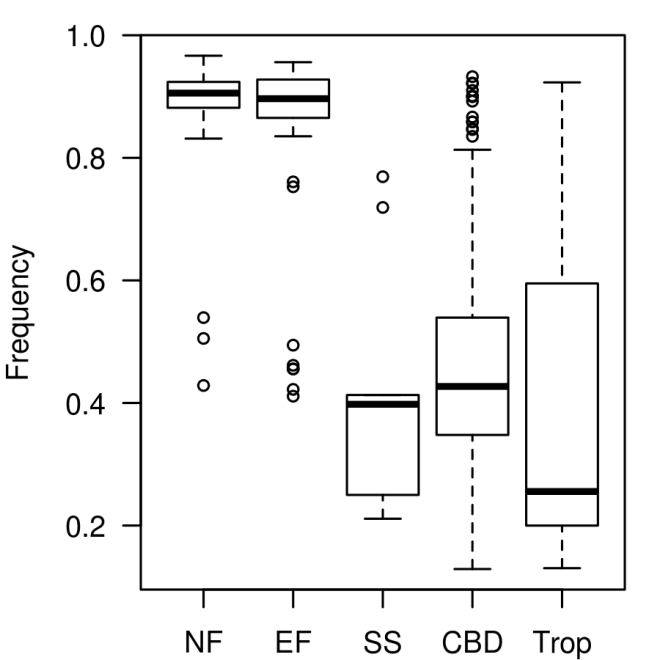
**

**Figure S22. Boxplot of the frequency of early alleles within each group.** Only non-admixed lines were considered, i.e. inbred lines with assignments above 0.8 considering five genetic groups obtained with STRUCTURE [40,41] using 55 SSRs [30].
