# Supplementary material for: Adaptation of Maize to Temperate Climates: Mid-Density Genome-Wide Association Genetics and Diversity Patterns Reveal Key Genomic Regions, with a Major Contribution of the Vgt2 (ZCN8) Locus
Source: PLoS One. 2013 Aug 30;8(8):e71377. doi: 10.1371/journal.pone.0071377 (PMC3758321; doi:10.1371/journal.pone.0071377)
Supplement: Figure S11 — Supporting Figure including one page per chromosome (10). (PDF) [file pone.0071377.s002.pdf]

Figure S11. Variation in diversity, differentiation, recombination, ancestral haplotype assignation and pairwise linkage disequilibrium measures along 10 chromosomes.

The 10 chromosomes were ordered and depicted on 10 different pages. Variations in different diversity, recombination and linkage disequilibrium measures were obtained using a sliding window approach that averages measures in a window of 1 Mb moving along the chromosomes by steps of 500 kb. Red vertical dashed lines with marker numbers starting by # indicate the position of SNPs significantly associated with female flowering time (FFLW8) and/or under selection, as described in Table 3. *Vgt1 Mite* [21] is indicated by a green vertical dashed line on chromosome 8. It is not represented on the 50K Illumina array. The closest marker to *Vgt1 Mit*e on the array is 3022 bp apart and is associated with FFLW8 with a *P*-value of 10^{-4}. Two other markers that are 16 kb from *Vgt1* are not associated with FFLW8. Centromere boundaries are indicated by two vertical black dashed lines. The horizontal dashed line for each index indicates the global mean of this measure on all chromosomes.

All diversity measures were estimated on 242 non-admixed lines for which assignments to one genetic group were higher than 0.8 based on the assignment results obtained with STRUCTURE software [40,41] using 55 SSR markers for five groups. The linkage disequilibrium measures and haplotype assignments were estimated on all inbred lines within the panel.

From the top to the bottom on each chromosome, different diversity measures are displayed. $\boldsymbol{F_{ST}}$ represents the differentiation index at SNPs estimated using the r-Hierfstat package [46]. $\boldsymbol{F_{STB}}$ represents the differentiation index of each SNP using BayeScan [47]. H_T represents overall diversity. $\boldsymbol{H_S}$ X represents diversity indexes within genetic group X, where X stands for NF, EF, CBD, Trop, i.e. Northern Flint, European Flint, Corn Belt Dent and Tropical, respectively [30]. $\boldsymbol{H_O}$ represents the observed heterozygosity. **Rec** corresponds to the probability of haplotype switch from one SNP to another calculated with fastPHASE algorithm, [55] using 20 haplotype clusters. It is linked to the ancestral recombination rate.. **Rec (Mb/cM)** represents the inverse of recombination in Mb/cM according to the IBM reference map (B73 x Mo17) obtained from [31], LHRF produced from the cross of European lines F2 x F252 giving similar results. $\boldsymbol{r^2}$ represents the linkage disequilibrium index estimated using Plink [50]. r^2_S is a linkage disequilibrium index that extracts the part of allelic frequency correlation (r^2) due to population structure [34]. $\boldsymbol{r^2}$**-** $\boldsymbol{r^2_S}$ represents the difference between linkage disequilibrium measures to estimate the strength of correction due to structure. **#Hap** represents (in black) the proportion of lines assigned locally to the main and the second haplotype clusters. Proportions of third to fifth haplotype clusters are represented in grey.

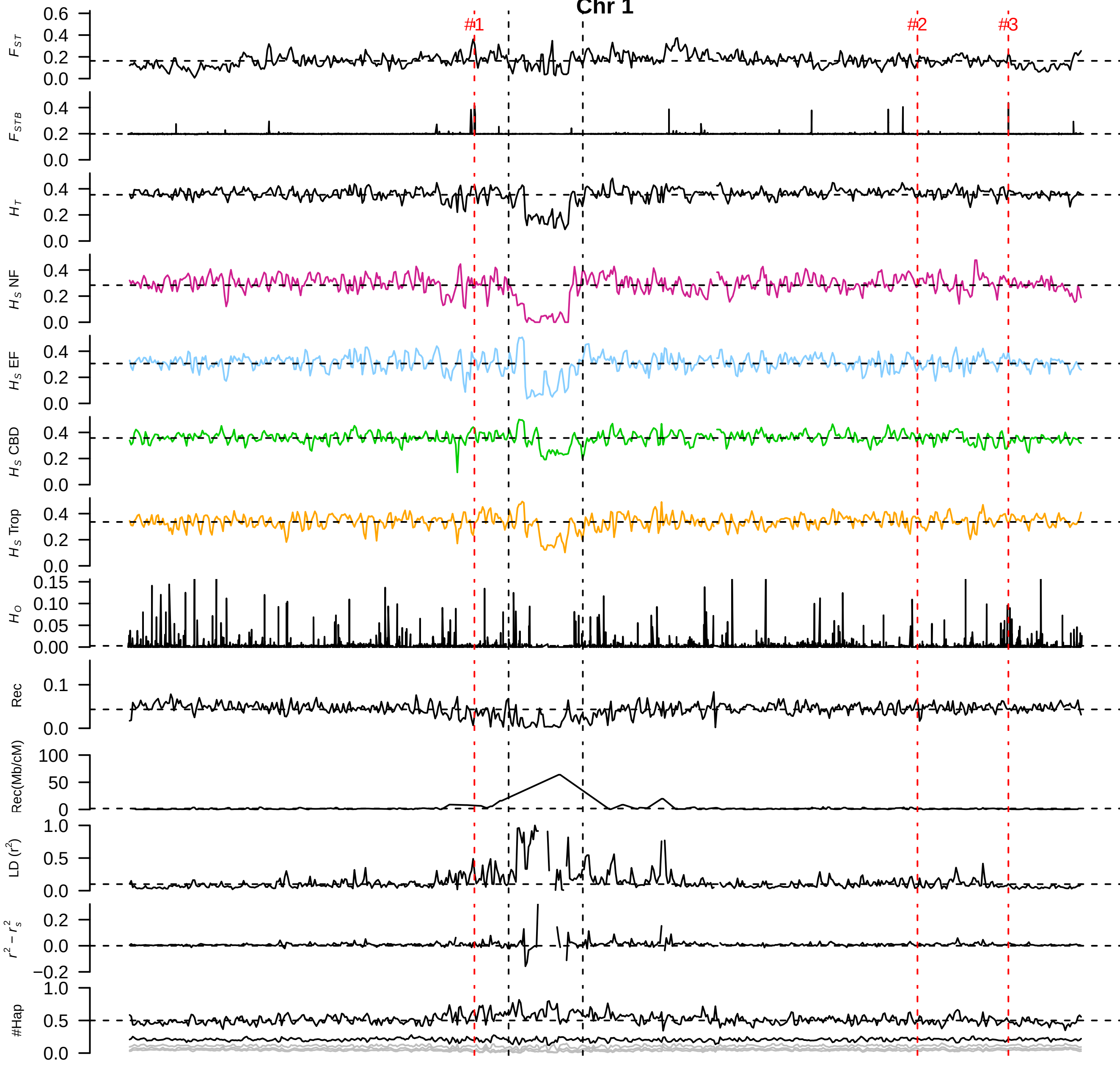

Chr 1
#1
#2
#3
F_{ST}
F_{STB}
H_T
H_S NF
H_S EF
H_S CBD
H_S Trop
H_O
Rec
Rec(Mb/cM)
LD (r^2)
$r^2 - r_s^2$
#Hap
0.6
0.4
0.2
0.0
0.15
0.10
0.05
0.00
0.1
100
50
0
1.0
0.5
−0.2

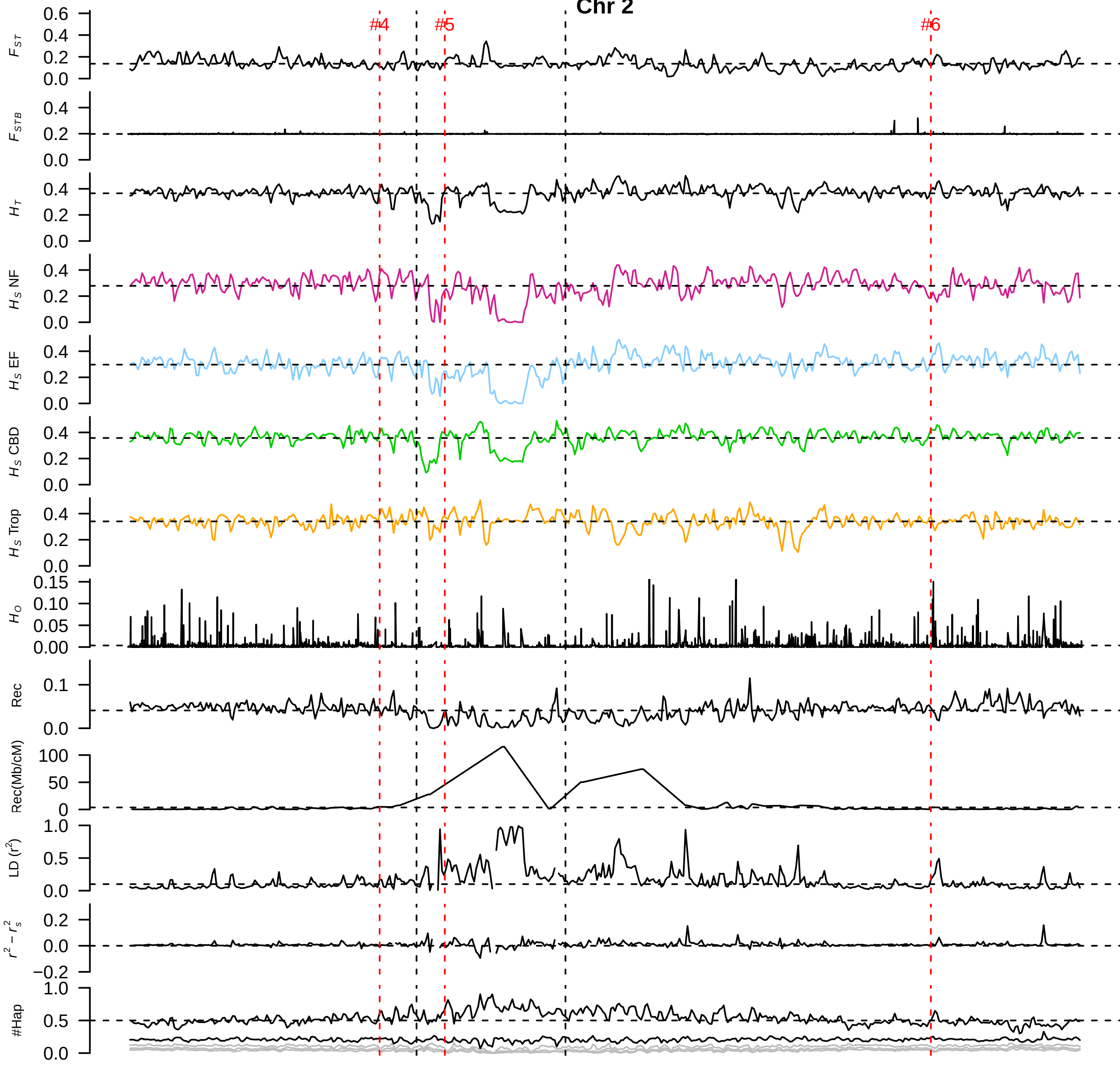
Chr 2
#4
#5
#6
F_{ST}
F_{STB}
H_T
H_S NF
H_S EF
H_S CBD
H_S Trop
H_O
Rec
Rec(Mb/cM)
LD (r^2)
$r^2 - r_s^2$
#Hap

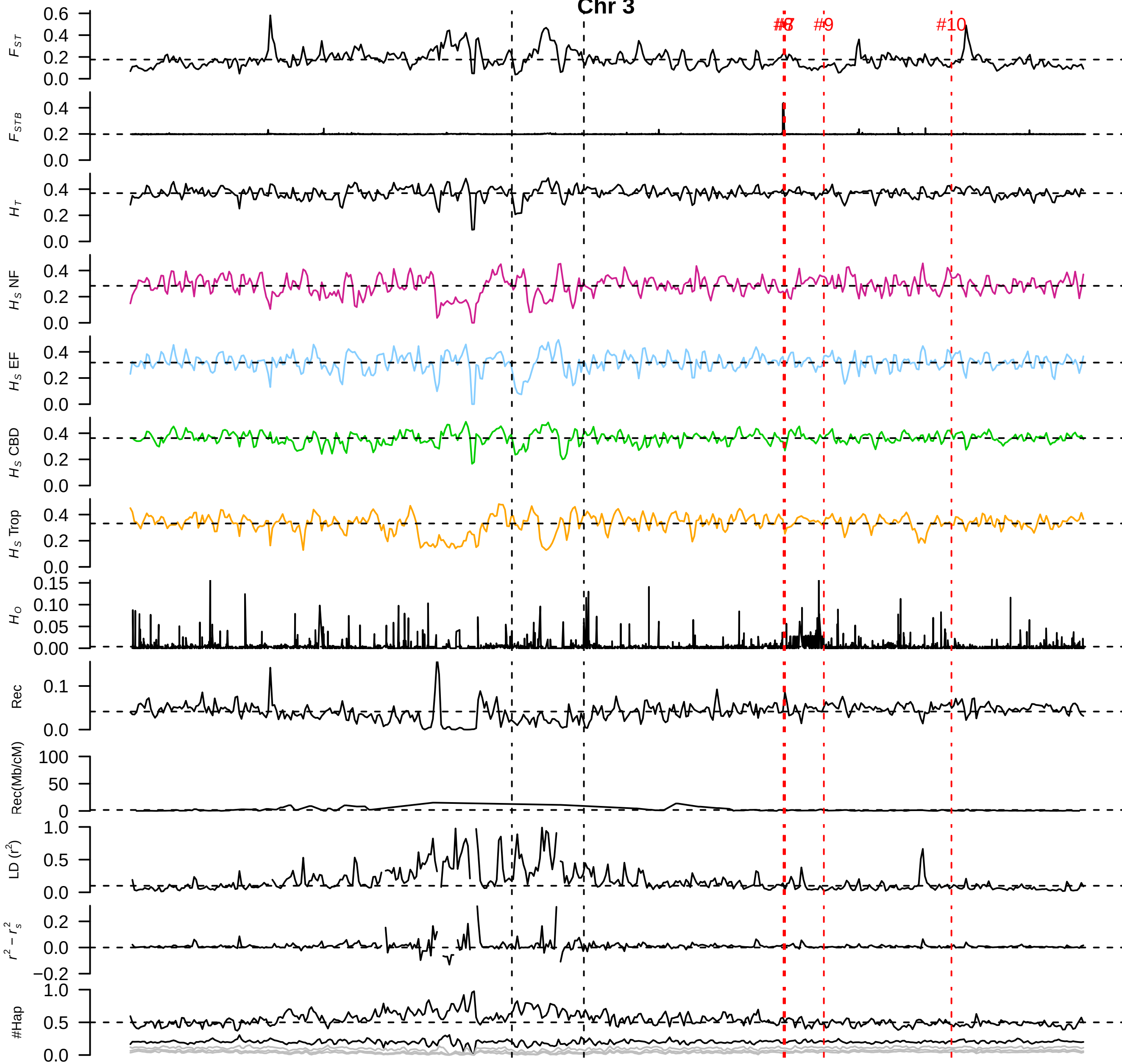
Chr 3
#7
#8
#9
#10
F_ST
F_STB
H_T
H_S NF
H_S EF
H_S CBD
H_S Trop
H_O
Rec
Rec(Mb/cM)
LD (r²)
r² − r_s²
#Hap
0.6
0.4
0.2
0.0
0.15
0.10
0.05
0.00
0.1
100
50
0
1.0
0.5
−0.2

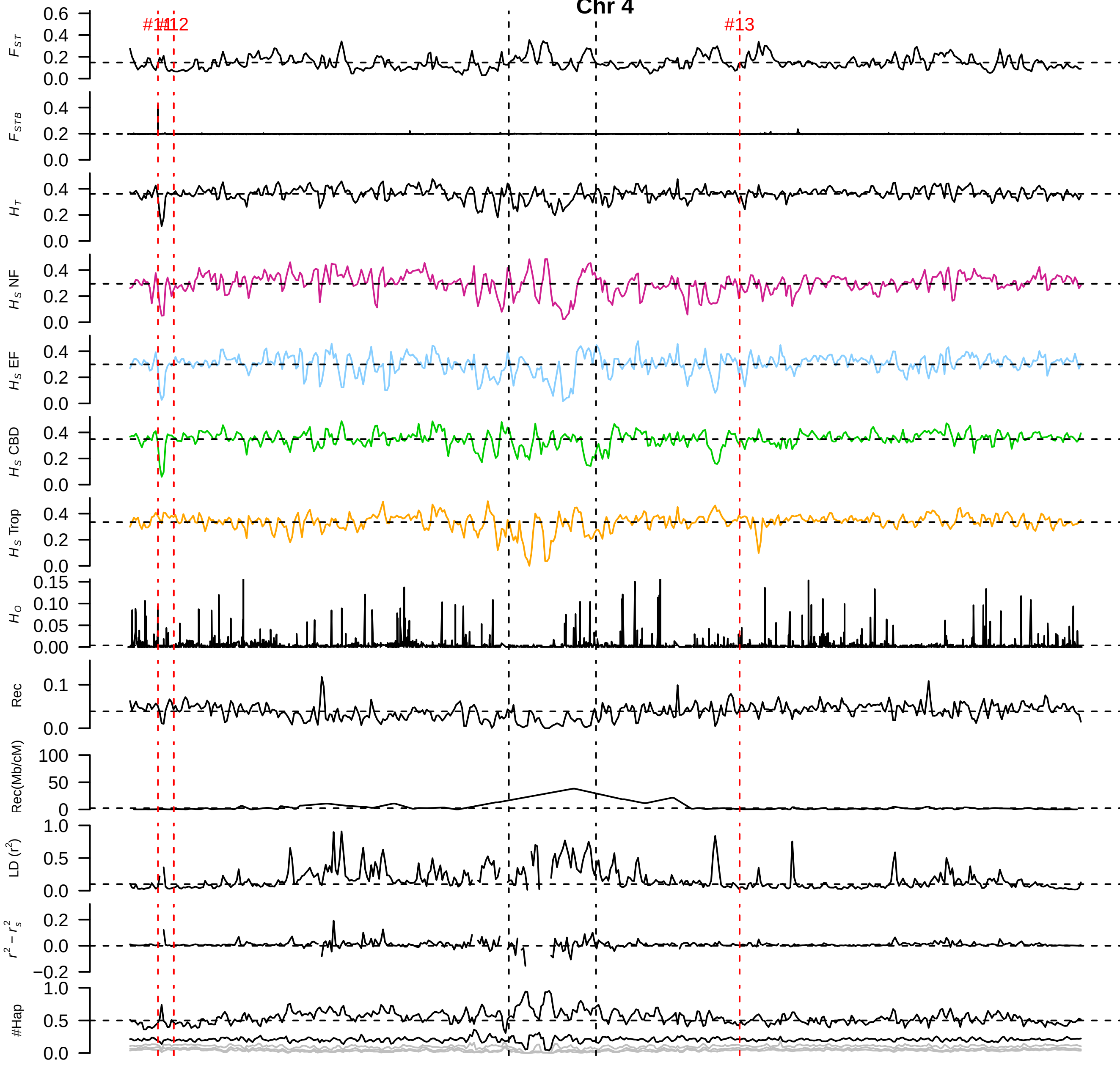

Chr 4
#11
#12
#13
F_{ST}
F_{STB}
H_T
H_S NF
H_S EF
H_S CBD
H_S Trop
H_O
Rec
Rec(Mb/cM)
LD (r^2)
$r^2 - r_s^2$
#Hap

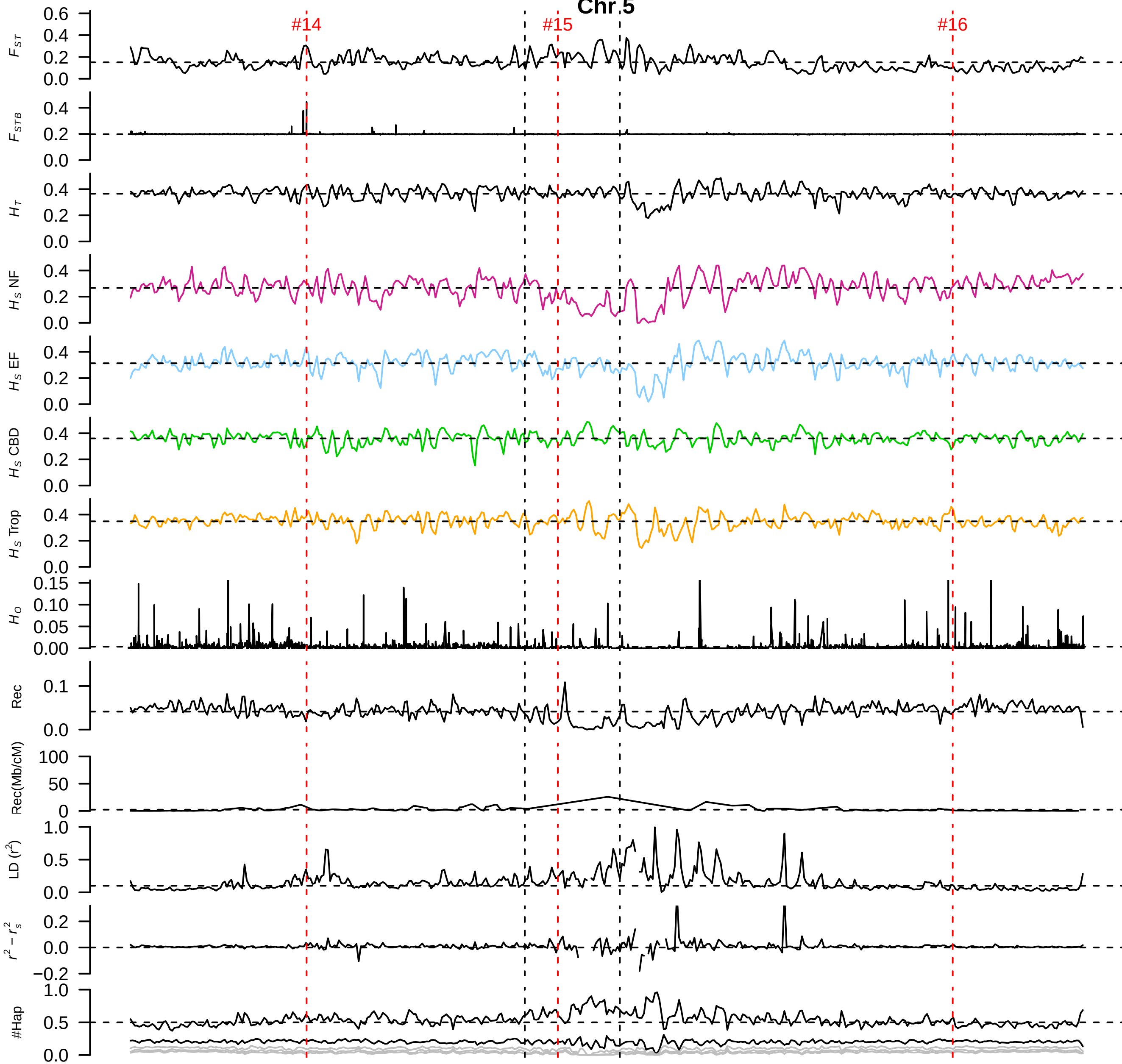

Chr.5
#14
#15
#16
F_{ST}
F_{STB}
H_T
H_S NF
H_S EF
H_S CBD
H_S Trop
H_O
Rec
Rec(Mb/cM)
LD (r^2)
$r^2 - r_s^2$
#Hap

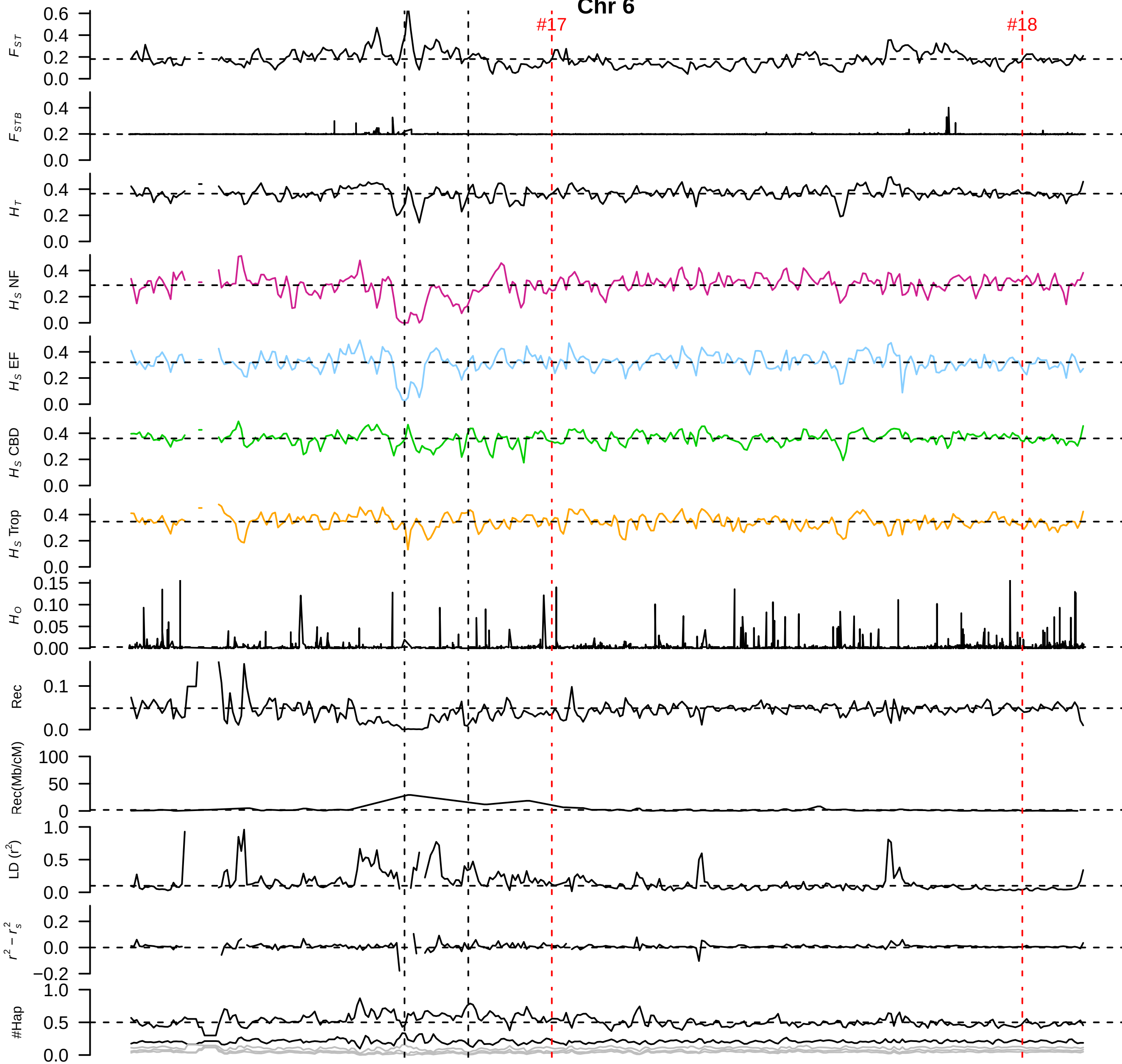
Chr 6
#17
#18
F_{ST}
F_{STB}
H_T
H_S NF
H_S EF
H_S CBD
H_S Trop
H_O
Rec
Rec(Mb/cM)
LD (r^2)
$r^2 - r_s^2$
#Hap

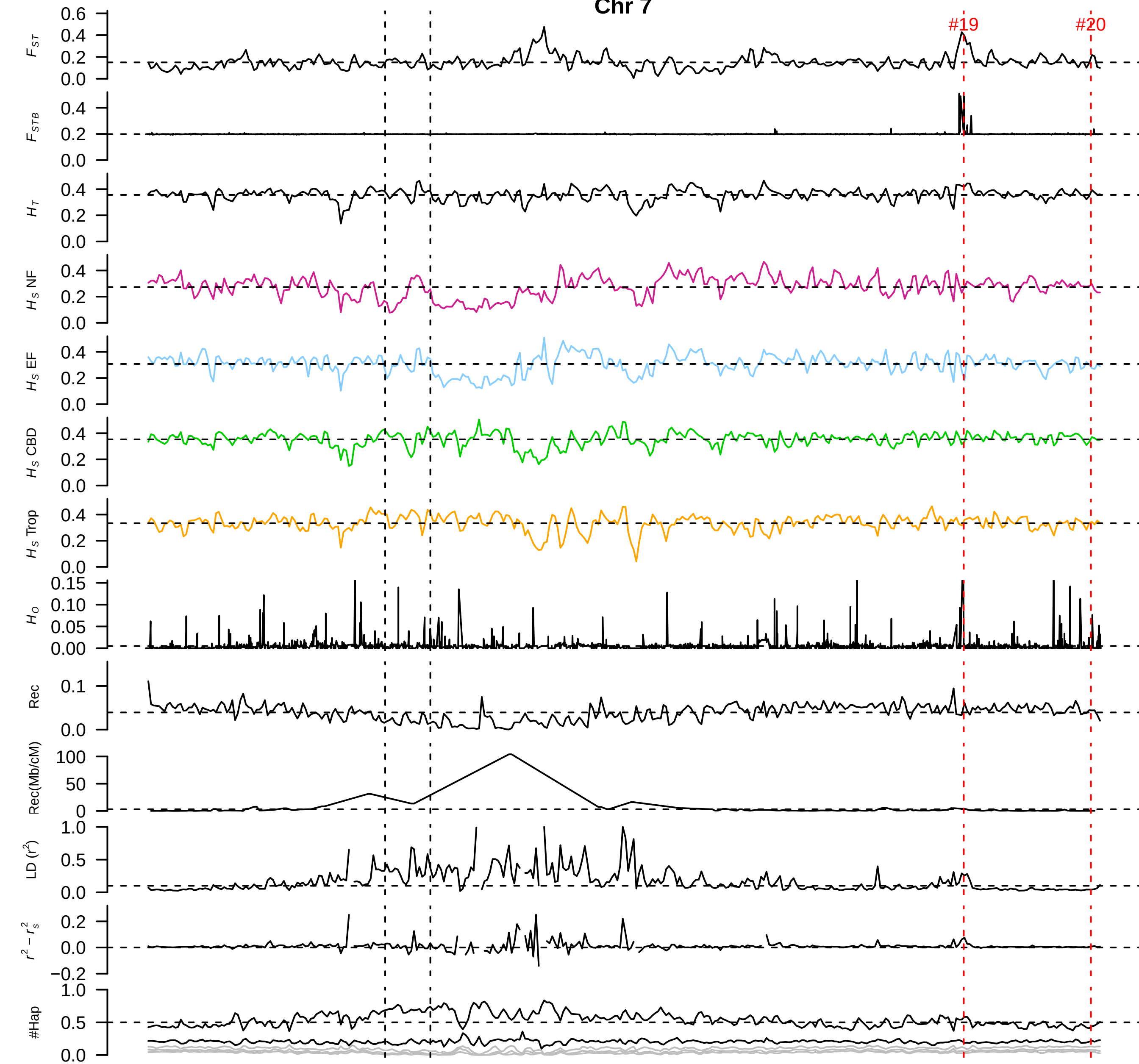

Chr 7
#19
#20
F_{ST}
F_{STB}
H_T
H_S NF
H_S EF
H_S CBD
H_S Trop
H_O
Rec
Rec(Mb/cM)
LD (r^2)
$r^2 - r_s^2$
#Hap

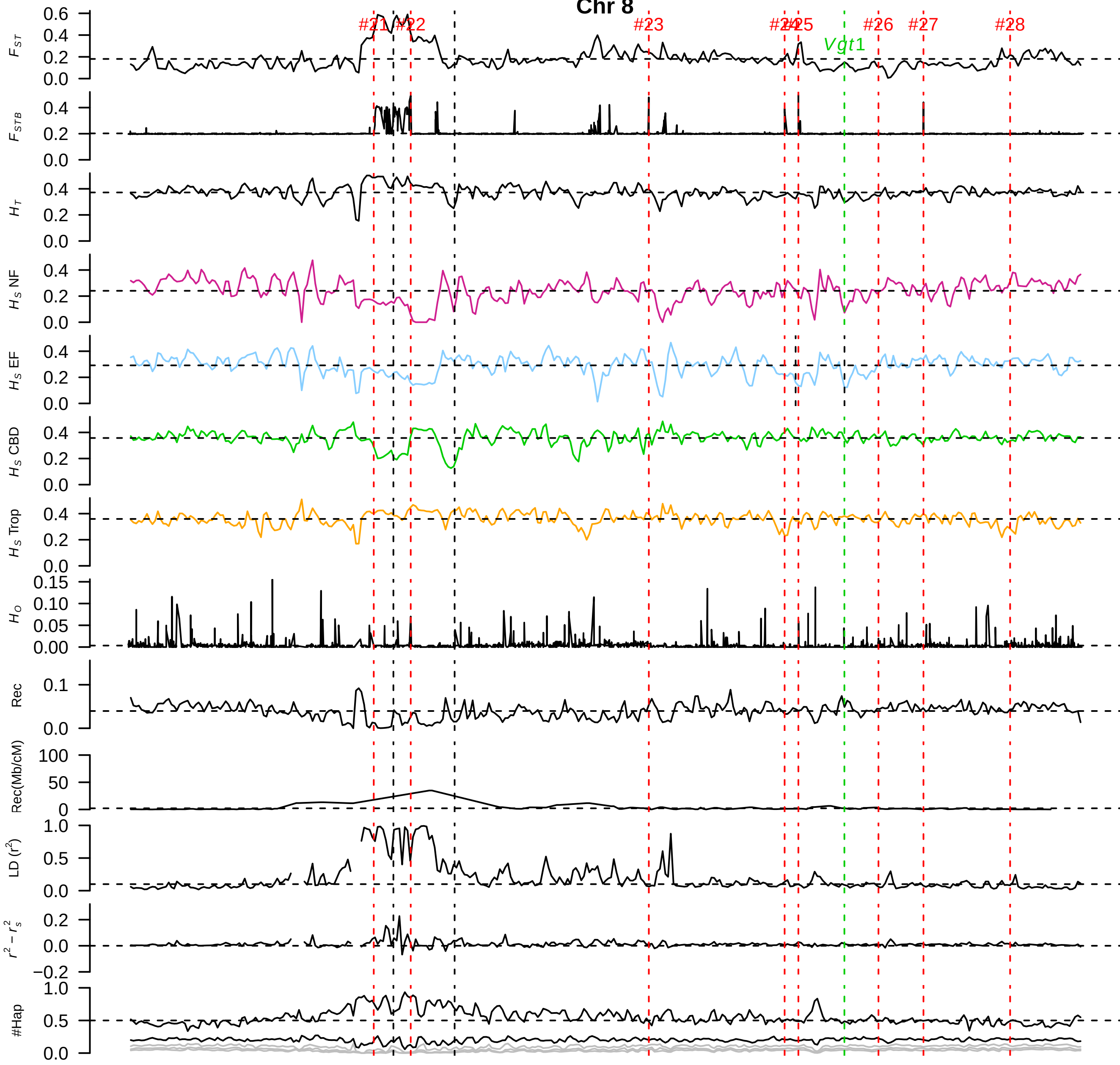

Chr 8
#21
#22
#23
#24
#25
Vgt1
#26
#27
#28
F_{ST}
F_{STB}
H_T
H_S NF
H_S EF
H_S CBD
H_S Trop
H_O
Rec
Rec(Mb/cM)
LD (r^2)
$r^2 - r_s^2$
#Hap

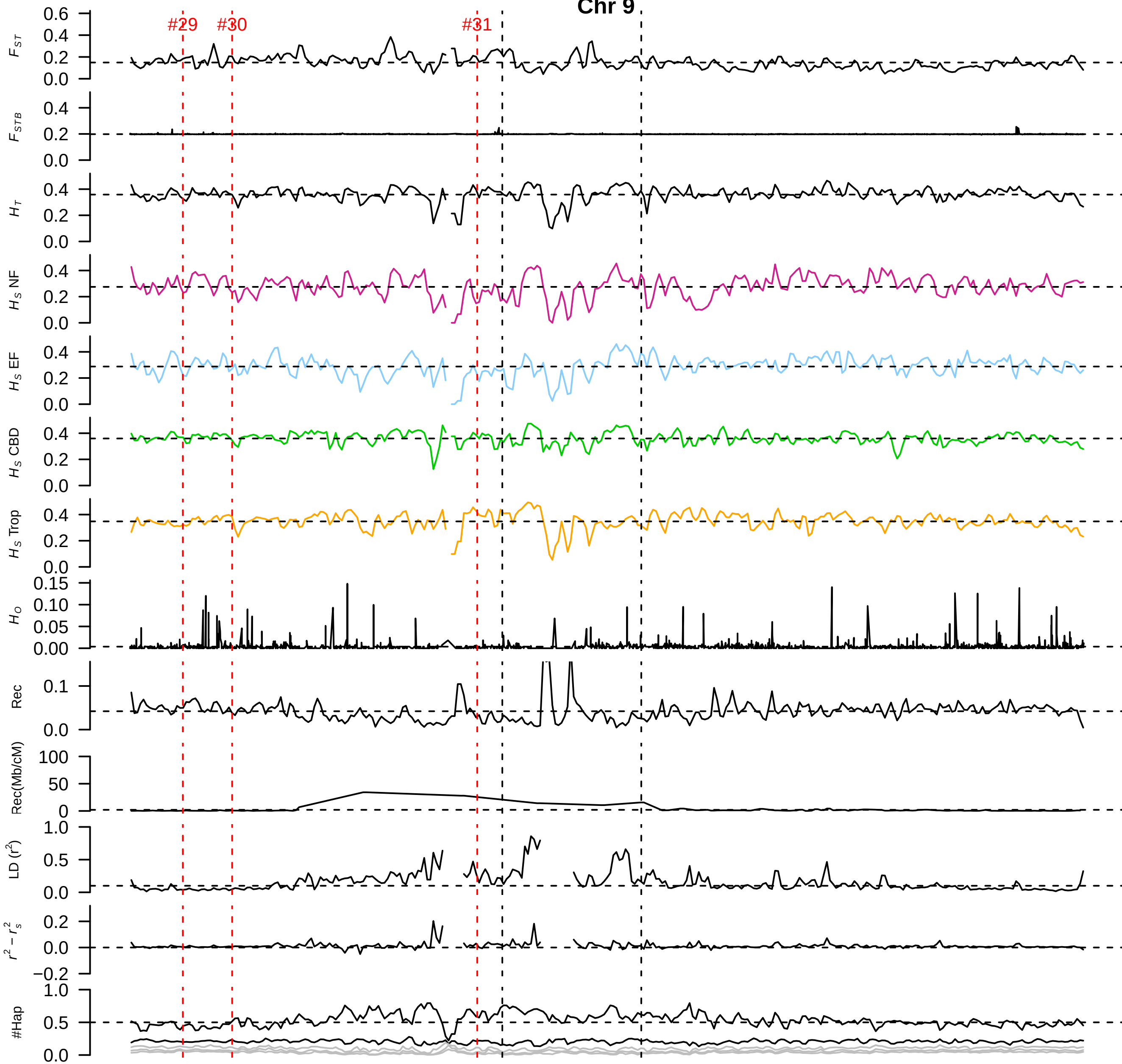

Chr 9
#29
#30
#31
F_{ST}
F_{STB}
H_T
H_S NF
H_S EF
H_S CBD
H_S Trop
H_O
Rec
Rec(Mb/cM)
LD (r^2)
$r^2 - r_s^2$
#Hap

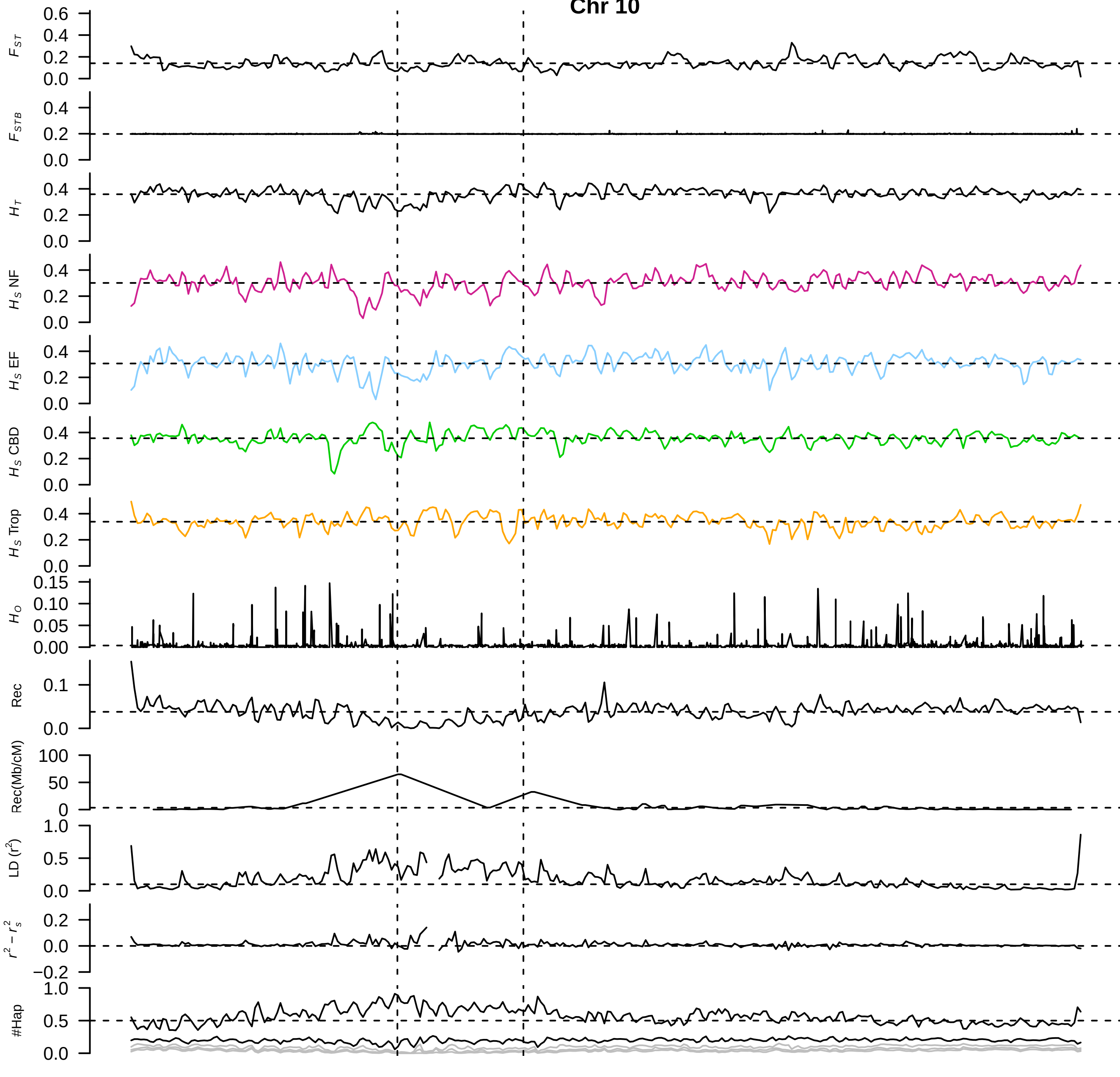

Chr 10
F_{ST}
0.6
0.4
0.2
0.0
F_{STB}
0.4
0.2
0.0
H_T
0.4
0.2
0.0
H_S NF
0.4
0.2
0.0
H_S EF
0.4
0.2
0.0
H_S CBD
0.4
0.2
0.0
H_S Trop
0.4
0.2
0.0
H_O
0.15
0.10
0.05
0.00
Rec
0.1
0.0
Rec(Mb/cM)
100
50
0
LD (r^2)
1.0
0.5
0.0
$r^2 - r_s^2$
0.2
0.0
−0.2
#Hap
1.0
0.5
0.0
