# Supplementary material for: Adaptation of Maize to Temperate Climates: Mid-Density Genome-Wide Association Genetics and Diversity Patterns Reveal Key Genomic Regions, with a Major Contribution of the Vgt2 (ZCN8) Locus
Source: PLoS One. 2013 Aug 30;8(8):e71377. doi: 10.1371/journal.pone.0071377 (PMC3758321; doi:10.1371/journal.pone.0071377)
Supplement: Figure S12 — Supporting Figure including one page per chromosome (10). (PDF) [file pone.0071377.s003.pdf]

Figure S12. Global representation along chromosomes of the most significant SNP-flowering time associations, meta QTL obtained by Chardon *et al.* [10], differentiation indexes F_{ST} and F_{STB}, overall diversity H_T and linkage disequilibrium pairwise measures. The 10 chromosomes were ordered and depicted on 10 different pages. Centromere boundaries are indicated by two vertical grey dashed lines. Red vertical dashed lines with marker numbers starting by # indicate the positions of SNPs significantly associated with female flowering time (FFLW8) and/or under selection (described in Table 3). From the top to the bottom: the first Manhattan plot shows the position of SNPs displaying the most significant associations (*P*-value < 10^{-5}) with female flowering time (FFLW8), male flowering time (MFLW8) and anthesis to silking intervals (ASI8) with the $Q_{55SSRs}+K_{IBS(94SSRs)}$ model. Blue rectangles correspond to meta QTL of flowering time, as described in [10]. The second Manhattan plot recalls additional associations for female flowering time (*P*-value < 10^{-3}). The next three plots show the variation in differentiation indexes F_{ST} and F_{STB} and the overall diversity H_T calculated for 242 non-admixed lines (assignment to one group > 0.8). F_{ST} corresponds to differentiation using r-Hierfstat [46]. F_{ST} and H_T were averaged over a sliding window of 1 Mb moving along the chromosomes by steps of 500 kb. F_{STB} corresponds to differentiation calculated with BayeScan [47]. Finally, the last graph displays pairwise LD (r^2 > 0.2) estimated with Plink [50], presented as a triangle plot.

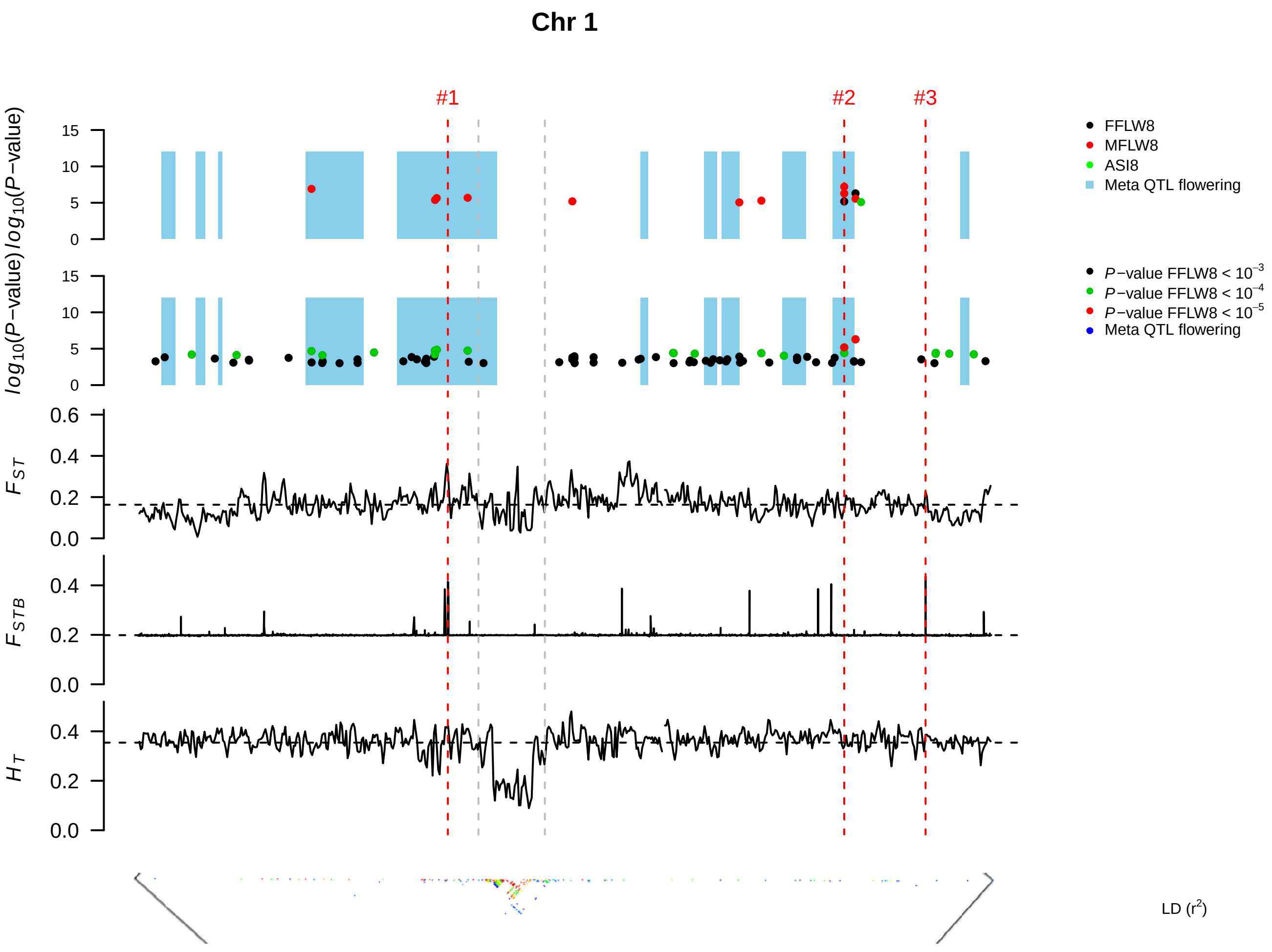

Chr 1
#1
#2
#3
log10(P−value)
log10(P−value)
FST
FSTB
HT
FFLW8
MFLW8
ASI8
Meta QTL flowering
P−value FFLW8 < 10−3
P−value FFLW8 < 10−4
P−value FFLW8 < 10−5
Meta QTL flowering
LD (r2)

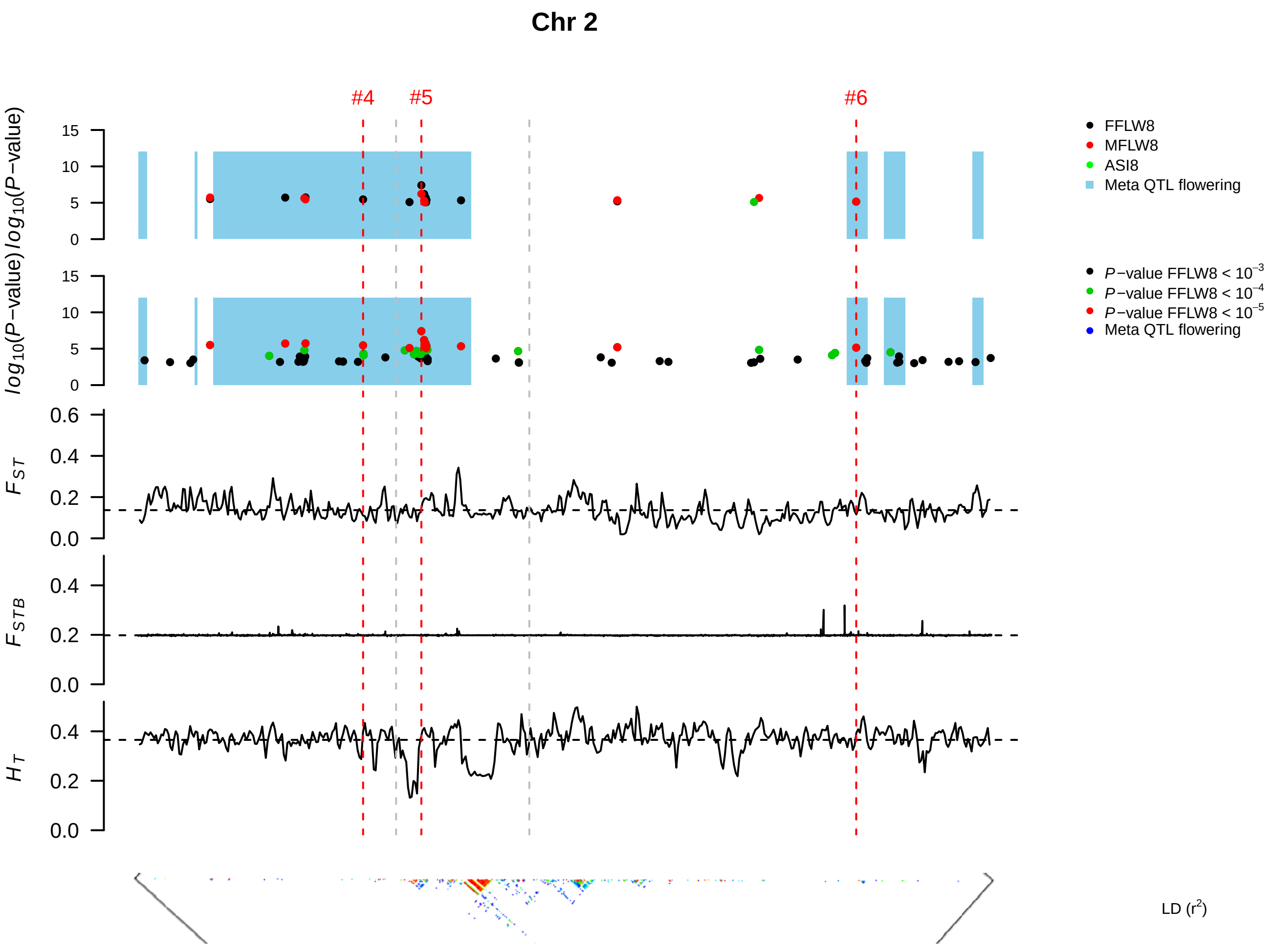

Chr 2
#4
#5
#6
FFLW8
MFLW8
ASI8
Meta QTL flowering
P−value FFLW8 < 10−3
P−value FFLW8 < 10−4
P−value FFLW8 < 10−5
Meta QTL flowering
log10(P−value)
log10(P−value)
FST
FSTB
HT
LD (r2)

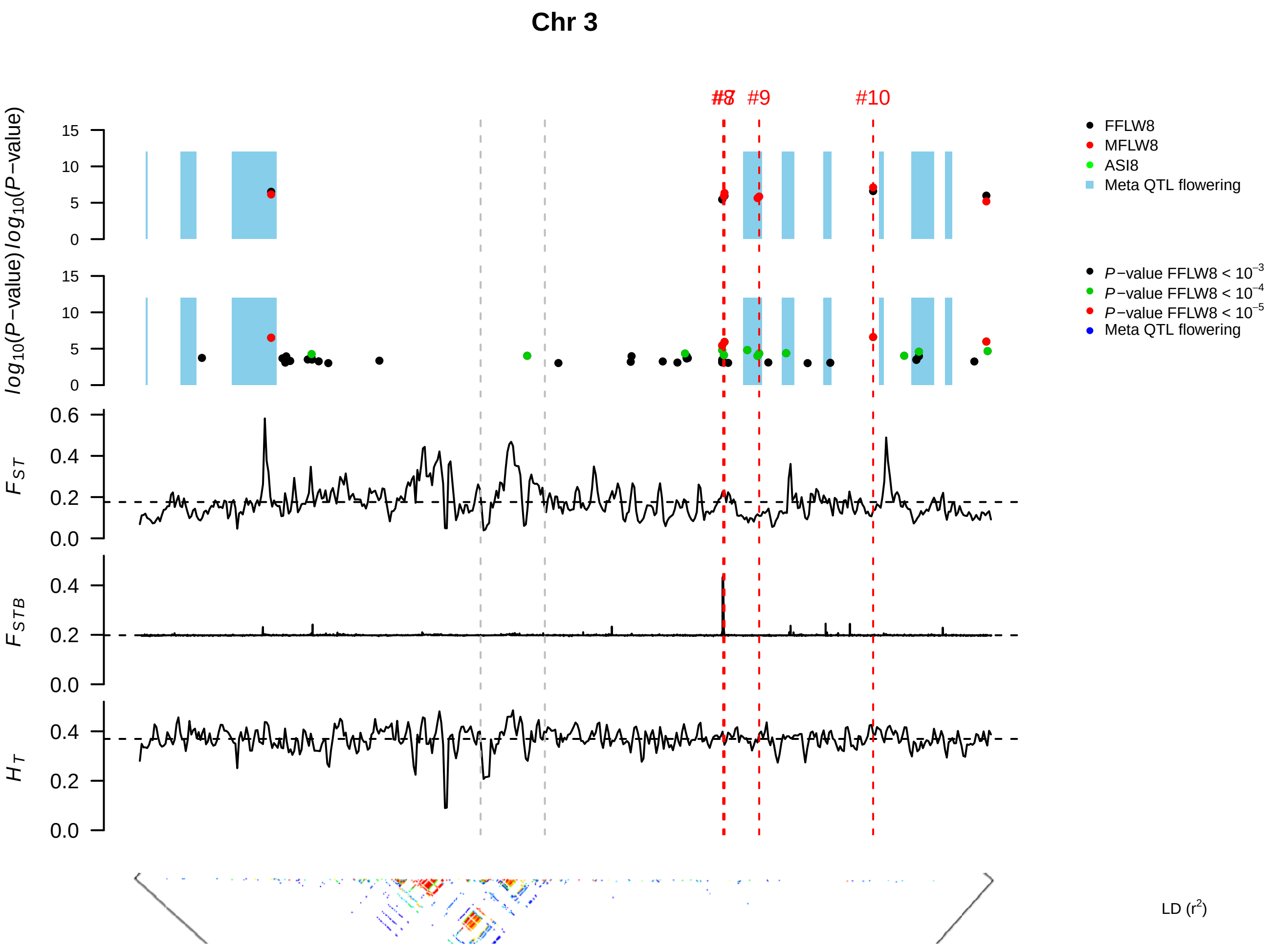
Chr 3
#7
#8
#9
#10
FFLW8
MFLW8
ASI8
Meta QTL flowering
P−value FFLW8 < 10−3
P−value FFLW8 < 10−4
P−value FFLW8 < 10−5
Meta QTL flowering
log10(P−value)
log10(P−value)
FST
FSTB
HT
15
10
5
0
0.6
0.4
0.2
0.0
LD (r2)

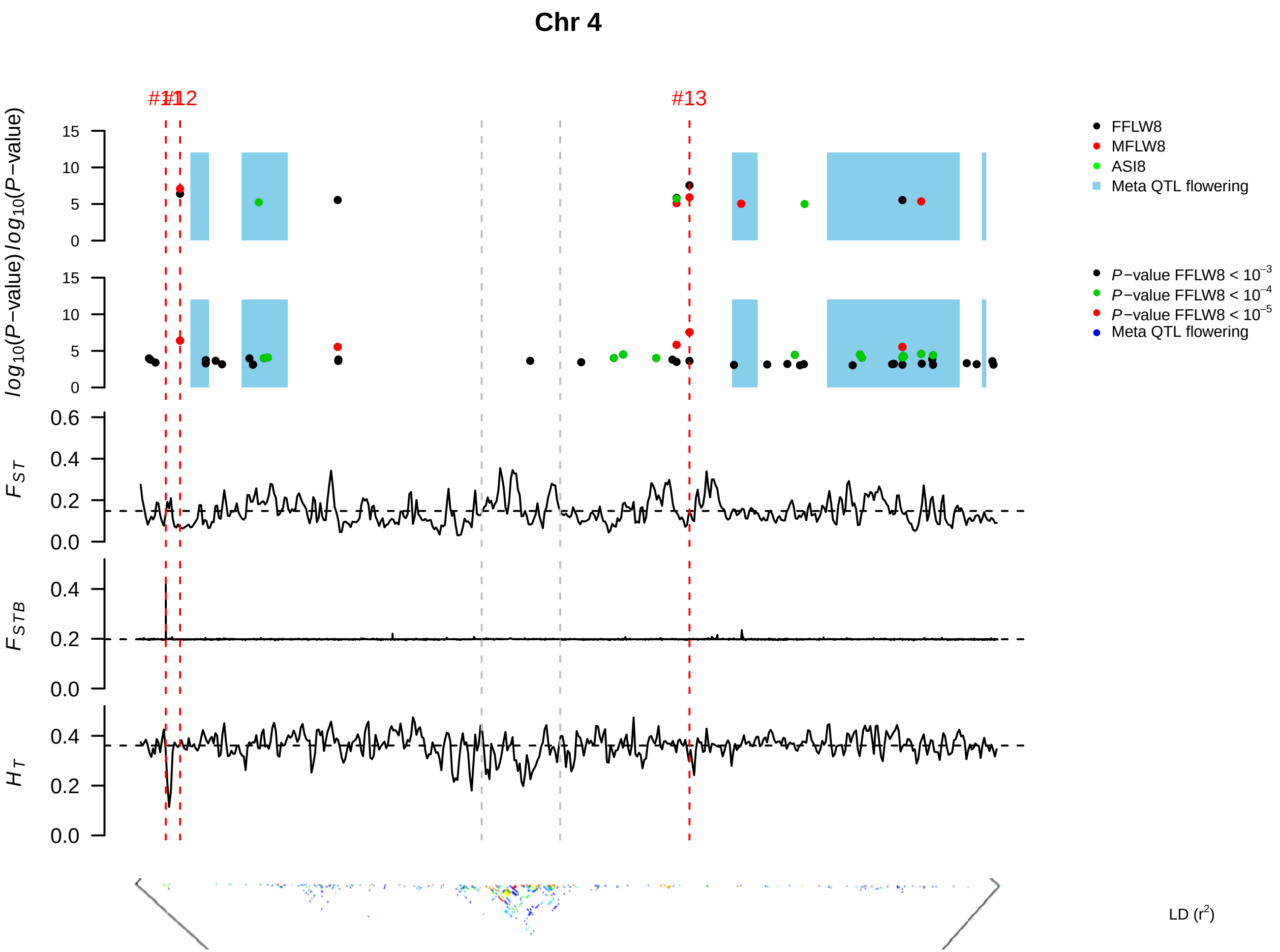

Chr 4
#11
#12
#13
FFLW8
MFLW8
ASI8
Meta QTL flowering
P−value FFLW8 < 10−3
P−value FFLW8 < 10−4
P−value FFLW8 < 10−5
Meta QTL flowering
log10(P−value)
log10(P−value)
FST
FSTB
HT
LD (r2)

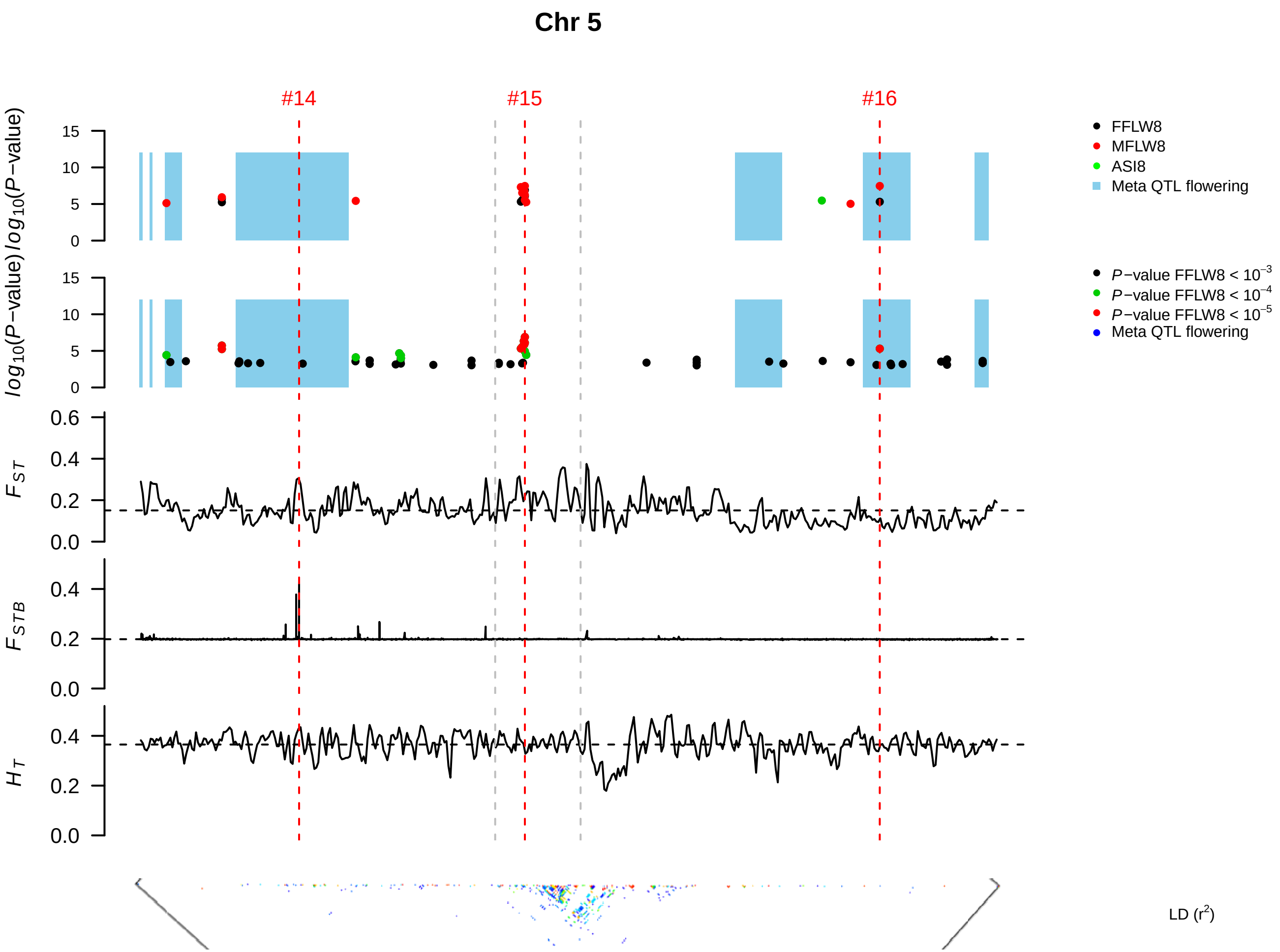

Chr 5
#14
#15
#16
FFLW8
MFLW8
ASI8
Meta QTL flowering
P−value FFLW8 < 10−3
P−value FFLW8 < 10−4
P−value FFLW8 < 10−5
Meta QTL flowering
log10(P−value)
log10(P−value)
FST
FSTB
HT
LD (r2)

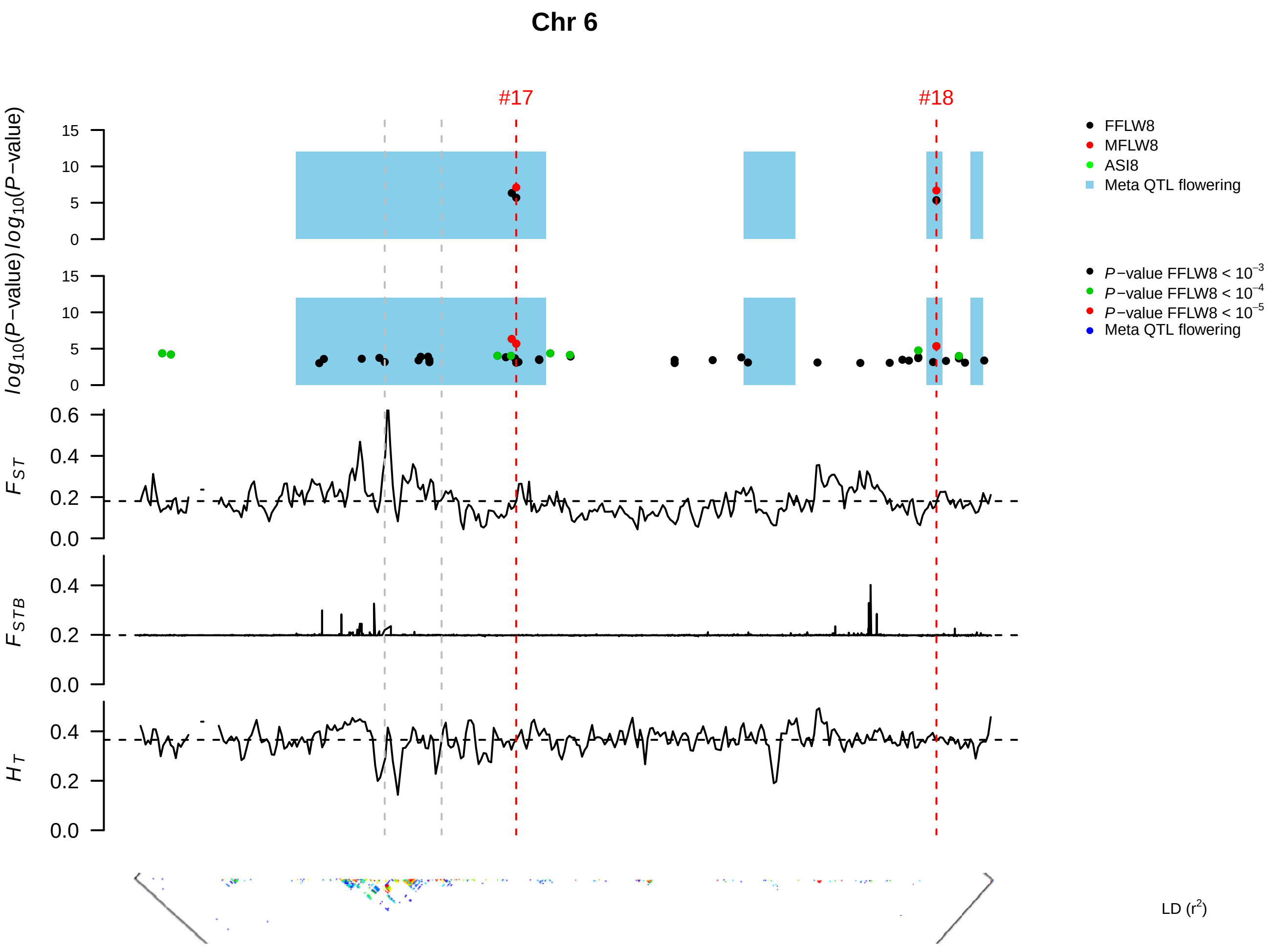

Chr 6
#17
#18
FFLW8
MFLW8
ASI8
Meta QTL flowering
P−value FFLW8 < 10−3
P−value FFLW8 < 10−4
P−value FFLW8 < 10−5
Meta QTL flowering
log10(P−value)
log10(P−value)
FST
FSTB
HT
LD (r2)

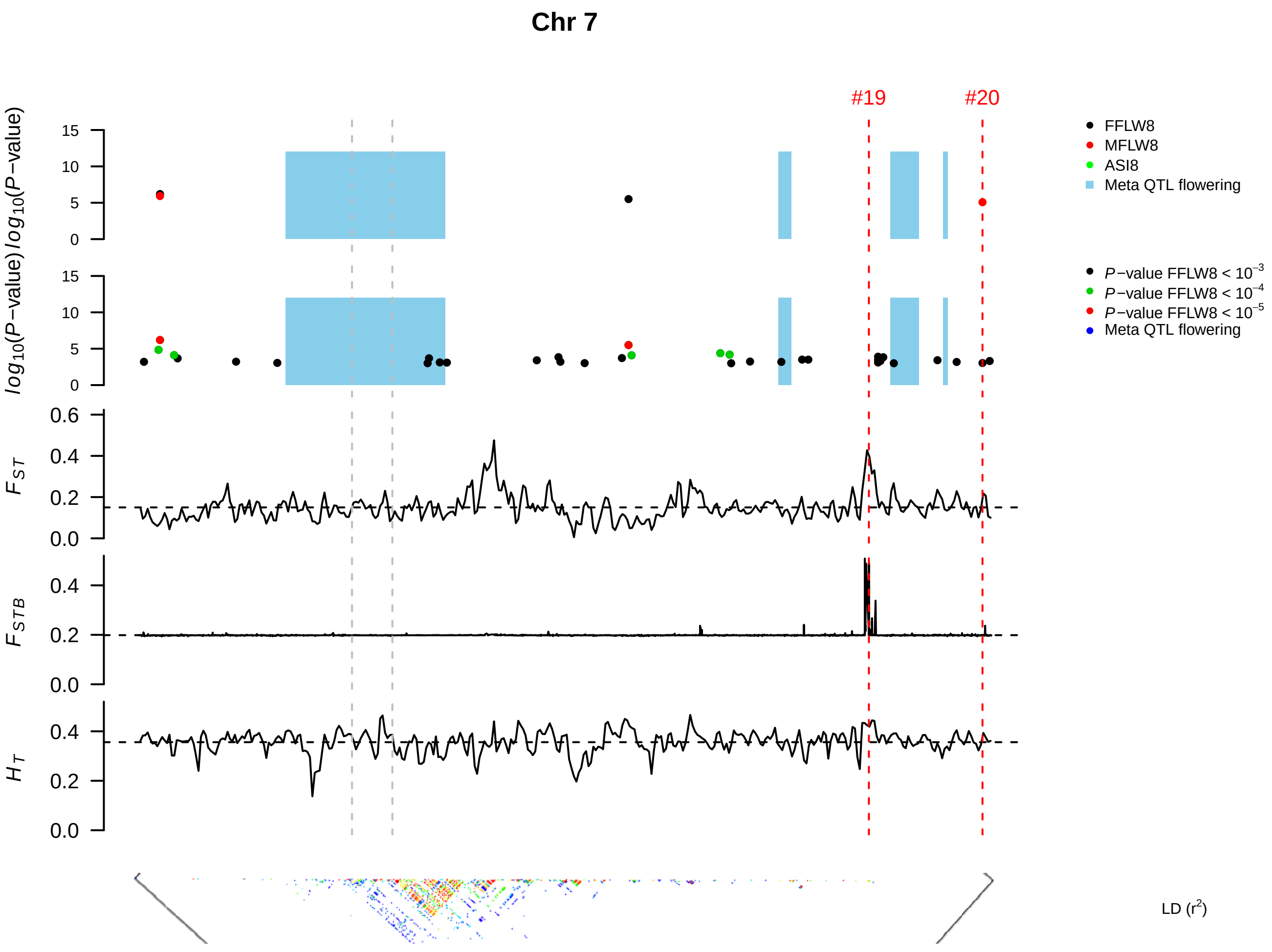

Chr 7
#19
#20
FFLW8
MFLW8
ASI8
Meta QTL flowering
P−value FFLW8 < 10−3
P−value FFLW8 < 10−4
P−value FFLW8 < 10−5
Meta QTL flowering
log10(P−value)
log10(P−value)
FST
FSTB
HT
LD (r2)

Chr 8

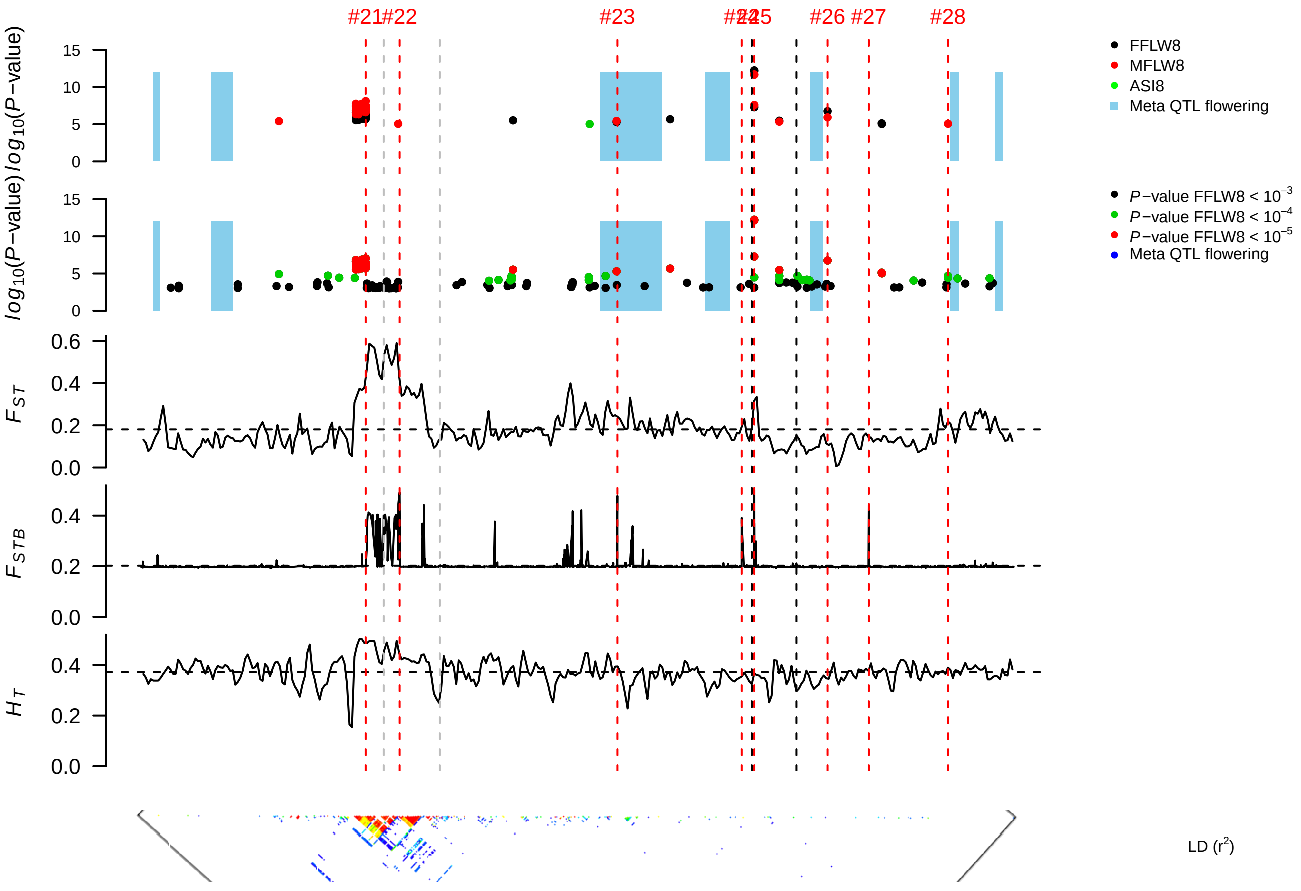

#21 #22
#23
#24 #25
#26 #27
#28
FFLW8
MFLW8
ASI8
Meta QTL flowering
P−value FFLW8 < 10^−3
P−value FFLW8 < 10^−4
P−value FFLW8 < 10^−5
Meta QTL flowering
log10(P−value)
FST
FSTB
HT
LD (r2)

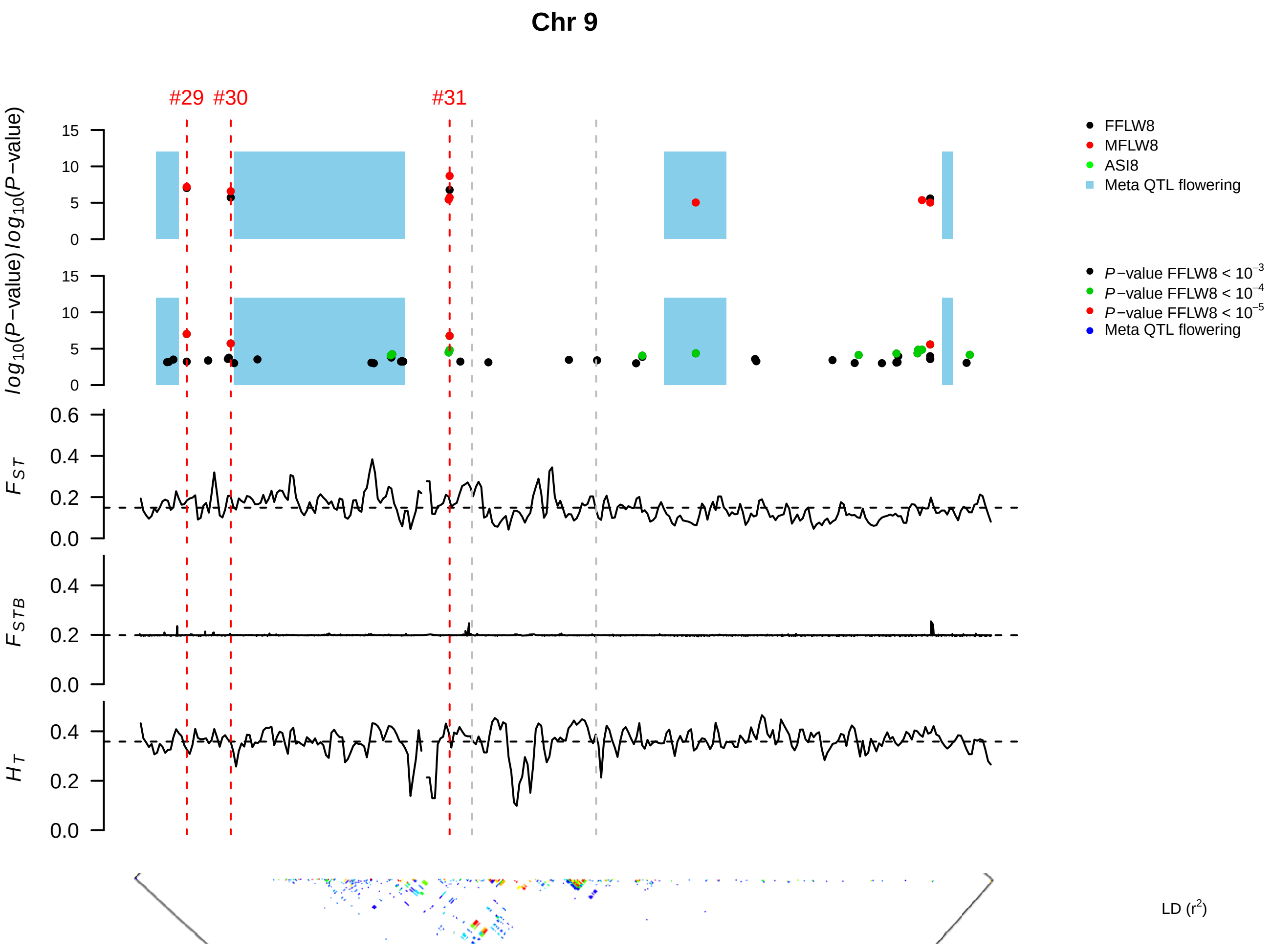

Chr 9
#29
#30
#31
$log_{10}(P\text{-value})$
$log_{10}(P\text{-value})$
15
10
5
0
FFLW8
MFLW8
ASI8
Meta QTL flowering
P-value FFLW8 < 10^{-3}
P-value FFLW8 < 10^{-4}
P-value FFLW8 < 10^{-5}
Meta QTL flowering
F_{ST}
0.6
0.4
0.2
0.0
F_{STB}
H_T
LD (r^2)

Chr 10

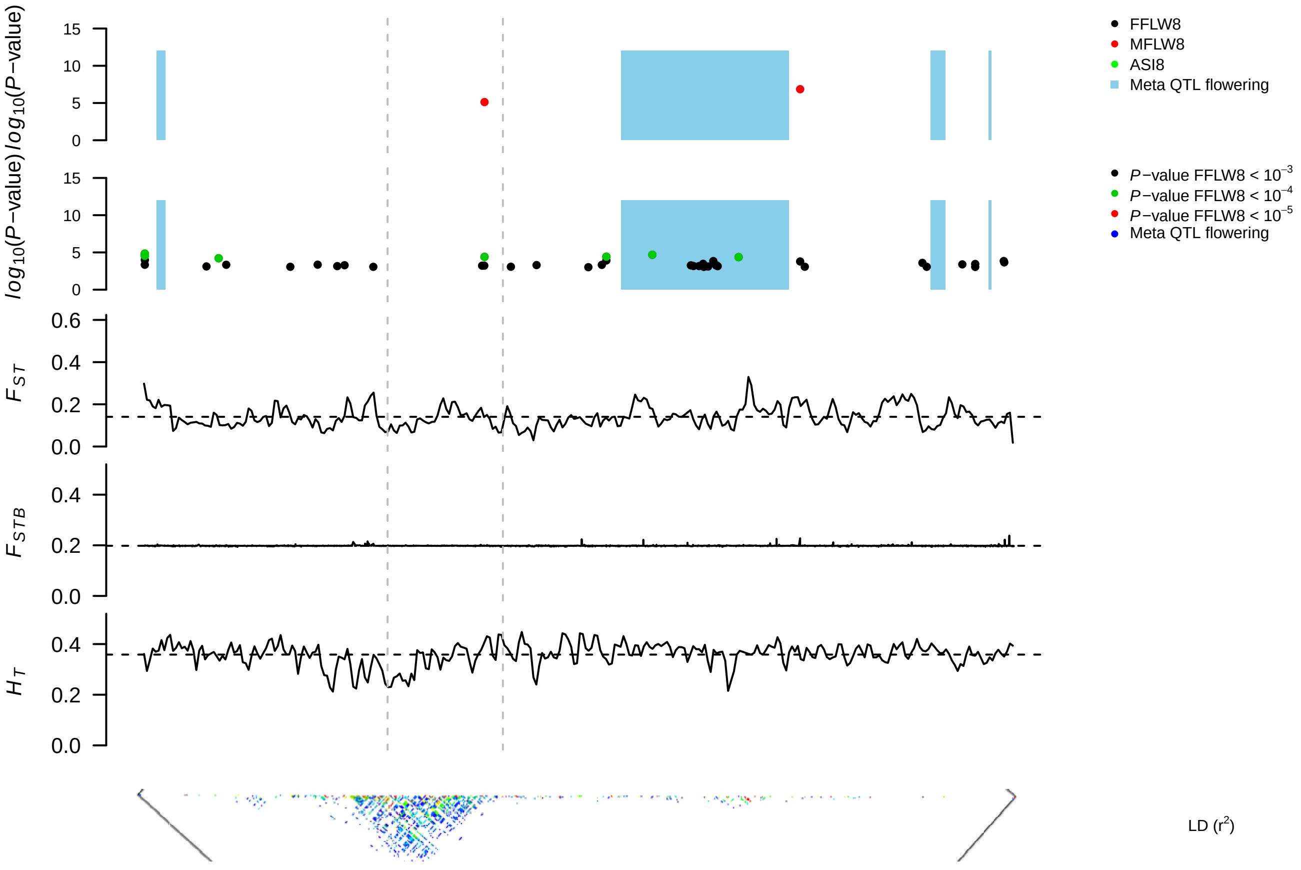

FFLW8
MFLW8
ASI8
Meta QTL flowering
P−value FFLW8 < 10−3
P−value FFLW8 < 10−4
P−value FFLW8 < 10−5
Meta QTL flowering
log10(P−value)
log10(P−value)
FST
FSTB
HT
LD (r2)
